# Evolution Equations on Co-evolving Graphs: Long-Time Behaviour and the Graph-Continuity Equation

**DOI:** 10.1007/s00332-026-10248-w

**Published:** 2026-03-05

**Authors:** José Antonio Carrillo, Antonio Esposito, László Mikolás

**Affiliations:** 1 Mathematical Institute, University of Oxford, Woodstock Road, Oxford, OX2 6GG UK; 2 Department of Information Engineering, Computer Science and Mathematics, Università degli Studi dell’Aquila, Via Vetoio 1, Coppito, 67100 L’Aquila, Italy

**Keywords:** Co-evolving graphs, Evolution on graphs, Long-time behaviour, Nonlocal equations, 35R02, 35A01, 35A02, 35A24, 35B40

## Abstract

We focus on evolution equations on co-evolving infinite graphs and establish a rigorous link with a class of nonlinear continuity equations, whose vector fields depend on the graphs considered. More precisely, weak solutions of the so-called graph-continuity equation are shown to be the push-forward of their initial datum through the flow map solving the associated characteristics’ equation, which depends on the co-evolving graph considered. This connection can be used to prove contractions in a suitable distance, although the flow on the graphs requires a too limiting assumption on the overall flux. Therefore, we consider upwinding dynamics on graphs with pointwise and monotonic velocity and prove long-time convergence of the solutions towards the uniform mass distribution.

## Introduction

In this manuscript, we continue the study of evolutionary partial differential equations (PDEs) on *co-evolving graphs* initiated in Esposito and Mikolás (2024). There, we analysed a model describing the evolution of some quantity, e.g. mass, distributed on the vertices of a graph and flowing through its weighted edges. In this model, the dynamics of the mass are coupled with the dynamics of the edge weights, meaning that the dynamics on the graph depends on those determining the graph itself. In many cases, the adaptive nature of *co-evolving* graphs is better suited for modelling many real-life phenomena than their static or simply evolving counterparts—when the edge weights also evolve in time, but their dynamics are not coupled with those on the graph. To mention a few examples, *co-evolving* graphs have been useful in biology, modelling neuronal systems (Clopath et al. 2010) or physiological networks (Sawicki et al. 2022), in machine learning, for modelling the training process of deep neural networks (Triesch 2005), and in epidemiological models, where individuals susceptible to some disease (the nodes) might want to break off the links (the edges) to other individuals because they are afraid of becoming infected (Demirel et al. 2017). We refer the reader to the review (Berner et al. 2023) for more applications of *co-evolving* graphs.

In this article, we build on the setting of Esposito et al. (2021, 2023), where the authors study the evolution of a (possibly negative) quantity on the vertices, a measure $\rho$ on a *static* graph, defined as follows.

### Definition 1.1

A static graph is given by a pair $\mathcal {G}:= (\mu , \eta )$, where $\mu \in \mathcal {M}^{+}(\mathbb {R}^d)$ is a positive Radon measure standing for (a subset of) the vertices and $\eta \in C_b({\mathbb {R}^{2d}_{\!\scriptscriptstyle \diagup }})$ is the edge-weight function, continuous and bounded, on the possible set of edges ${\mathbb {R}^{2d}_{\!\scriptscriptstyle \diagup }}:= \{(x,y) \in \mathbb {R}^{2d} \ | \ x \ne y\}$.

In view of the previous definition, self-loops are not allowed, and for example, any finite set of vertices, $\{x_1,\ldots ,x_n\} \subset \mathbb {R}^{d}$, corresponds to empirical measures such as $\mu ^n=\frac{1}{n}\sum _{i=1}^n\delta _{x_i}$, where $\delta _{x_i}$ denotes a Dirac-mass at each $x_i\in \mathbb {R}^d$, for $i=1,\ldots , n$. The same setting allows to easily represent large networks by taking, e.g. $\mu ^\infty = \lim _{n\rightarrow \infty }\mu ^n$, interpreting the limit in a suitable weak-sense. In order to provide general stability results, the domain of the edge-weight function is defined outside of $\mathop {\textrm{supp}}\limits (\mu )^2$ to allow for cases where initially part of the support of $\rho$ is outside of the support of $\mu$, cf. (Esposito et al. 2021, Theorem 3.14). In many applications in machine learning, the edge-weight $\eta$ is a function of the distance, that is $\eta = f(|x-y|)$, for *f* continuous on $\{f\ne 0\}$. An important example is given by geometric graphs with connectivity $\varepsilon >0$ and weight being the characteristic function of a ball of radius $\varepsilon$. Definition 1.1 includes both finite and uncountably infinite weighted graphs.

In Esposito and Mikolás (2024), some of the previous results are extended to the *co-evolving* setting, and it is studied the well-posedness of the following *co-evolving* nonlocal conservation law

Co-NCL $$\begin{aligned} {\begin{matrix} & \partial _t \rho _t = - \overline{\nabla }\cdot F^\Phi [\mu , \eta _t ; \rho _t,V_t[\rho _t]],\\ & \partial _t \eta _t = \omega _t[\rho _t] -\eta _t, \end{matrix}} \end{aligned}$$

where, for $t\in [0,T]$, the edge-weight $\eta _t$ is no longer static and its dynamics depend on the “mass” distribution on the vertices, $\rho _t$, through a (potentially nonlocal) function $\omega$. Such a choice for the evolution of $\eta$ allows for an investigation of its time scales: slower and faster than the flow defined on the graph. Indeed, the former produces static graphs, whereas the latter gives rise to graphs whose weight functions depend on the density at the vertices, i.e. $\eta =\omega [\rho ]$, cf. (Esposito and Mikolás 2024, Section 4). As for the equation for $\rho$, it is a nonlocal continuity equation where $\overline{\nabla }\cdot$ is the graph divergence (see Definition 2.1), $F^\Phi$ is the flux, and *V* is a solution-dependent velocity. Differently from evolutions on $\mathbb {R}^d$, note that, on the graph, the mass is distributed on the vertices, whereas the velocity and the flux are defined on the edges. In particular, to define the flux we need a suitable function, $\Phi$, interpolating the mass at the vertices connected by a given edge to have edge-based quantities. For this reason, the flux depends on $\Phi$ as the notation $F^\Phi$ indicates, cf. Definition 2.2. We refer the reader to Sect. 2 for further details.

In this paper, differently from Esposito and Mikolás (2024), we shall focus on the case where the measure defining the set of vertices, $\mu \in \mathcal {P}_2(\mathbb {R}^d)$, is a probability measure with finite second moments and $\rho \ll \mu$ with $r:= \frac{\textrm{d}\rho }{\textrm{d}\mu }$. Under suitable assumptions on $\Phi$ and $\eta$, system (Co-NCL) can be rewritten as, for $\mu$-a.e. $x\in \mathbb {R}^d$,

Euler Co-NCL $$\begin{aligned} \begin{aligned} \partial _t r_t(x)&= - \int _{\mathbb {R}^d\backslash \{x\}}\Phi (r_t(x),r_t(y); V_t[r_t](x,y))\eta _t(x,y)\textrm{d}\mu (y),\\ \partial _t \eta _t&= \omega _t[r_t] -\eta _t, \end{aligned} \end{aligned}$$

where the equation for *r* is a nonlinear Euler equation, motivating the label chosen. Starting from (Euler Co-NCL), first we prove existence and uniqueness of solutions to (Euler Co-NCL) as curves in the space $C([0,T], {L^p_\mu (\mathbb {R}^d)})\times C([0,T], C_b({\mathbb {R}^{2d}_{\!\scriptscriptstyle \diagup }}))$, $p=2,\infty$, equipped with a suitable distance we specify later in Sect. 3. We recast the well-posedness of (Co-NCL) in Esposito and Mikolás (2024) to the $L^p_\mu$ setting, which is needed to study the two main aspects of this paper that are not considered in Esposito and Mikolás (2024): the associated graph-continuity equation and the long-time behaviour of its solutions. First, we shall see that, for $\rho \ll \mu$, we can link (Co-NCL) to a particular nonlinear continuity equation on $\mathbb {R}^{d+1}$, which we shall call the *graph-continuity equation*. More precisely, for a given pair $(r, \eta )$ solution of (Euler Co-NCL) with base measure $\mu \in \mathcal {P}_2(\mathbb {R}^d)$, the measure $\sigma := \delta _r \otimes \mu$ defined by

1.1 $$\begin{aligned} ( \delta _r\otimes \mu )(A \times B) = \int _{B}\delta _{r(x)}(A) \textrm{d}\mu (x), \end{aligned}$$

on any Borel set $A\times B \in \mathcal {B}(\mathbb {R}\times \mathbb {R}^d)$, is a weak solution to the following continuity equation:

graph-CE $$\begin{aligned} {\left\{ \begin{array}{ll} \partial _t \sigma + \partial _{\xi }(\sigma \mathcal {X}[\sigma , r, \eta ]) = 0, \\ \sigma _0 = \bar{\sigma } \in \mathcal {P}(\mathbb {R}\times \mathbb {R}^d), \end{array}\right. } \end{aligned}$$

where $\xi \in \mathbb {R}$ and for any $\sigma \in \mathcal {P}(\mathbb {R}\times \mathbb {R}^d)$, the velocity field is given by

1.2 $$\begin{aligned} \mathcal {X}[\sigma ,r, \eta ](t,\xi ,x):=-\int _{\mathbb {R}\times \mathbb {R}^d\backslash \{x\}}\Phi ( \xi , \xi '; V_t[r_t](x,x'))\eta _t(x,x')\textrm{d}\sigma (\xi ',x'), \end{aligned}$$

and it depends on a solution $(r,\eta )$ to (Euler Co-NCL). We refer to (graph-CE) as the *graph-continuity equation* since the velocity field depends, indeed, on the solution of the co-evolving graph problem. The mass displacement over the vertices is now expressed through a structured transport equation since the vertices of the graph are not evolving in time. In particular, we look at the probability density of having a certain mass $\xi$ at the vertex *x*, acting as a “parameter”. Notice that (graph-CE) is a system of transport PDEs indexed by *x*. By looking at the disintegrated version of (graph-CE), we prove that weak solutions can be constructed as the push-forward of the initial datum through the corresponding flow map. We embed (Euler Co-NCL) into (graph-CE) in order to study the long-time asymptotics via contractions in a suitable distance, as in Sect. 4.2. Obtaining contractivity of the flow is, indeed, difficult and still open for solutions to (Co-NCL) in view of the non-standard possible Finslerian nature of the equation for $\rho$, cf. Esposito et al. (2021). Working with (graph-CE), we obtain contraction of the solutions, although the structure of the flow somehow requires a too strong condition on the overall flux which does not seem to be satisfactory, since it indicates that all vertices should loose mass, see the condition on the velocity given in Proposition 4.3. With the aim of obtaining a better result in this direction, the second contribution of this manuscript is the study of the long-time behaviour of (Euler Co-NCL) for the upwind interpolation and a particular type of velocity fields that we call *pointwise*—they can be written as $V_t[r](x,x') = V_t(r_t(x),r_t(x'))$, for any vertices $x,x' \in \mathbb {R}^d$ — and *monotonic* (see Definition 5.1), which intuitively means that vertices will send mass to neighbours with less mass and gain mass from those with more mass. In view of the pointwise nature of the velocity fields, we obtain the well-posedness of (Euler Co-NCL) for $r \in L^\infty _\mu (\mathbb {R}^d)$ by the Banach fixed-point theorem in Theorem 5.1. We then move to the study of the long-time behaviour of (Euler Co-NCL) in this framework. To this end, we restrict the co-evolving graph to be defined on a compact set $K \subset \mathbb {R}^d$ and fix the upwind interpolation $\Phi _\textrm{upwind}$. We obtain the long-time asymptotics by showing that the supremum norm of *r* decreases monotonically in time, while the infimum increases monotonically in time, resulting in a stabilisation of the dynamics at $\frac{M}{\mu (K)}$, the uniform mass distribution, where $\int _{\mathbb {R}^d}r_0(x)\textrm{d}\mu (x) = M$. As by-product, we provide long-time asymptotics for the $\mu$-monokinetic solution of (graph-CE).

Passing from (Euler Co-NCL) to (graph-CE) resembles the link between the graph-limit and the mean-field limit for interacting particle systems (IPSs) with labels, cf., e.g. Paul and Trélat (2024) and the references therein. This approach, in a different framework with respect to the one presented in this paper, concerns IPSs with heterogeneous interactions, depending on the particles’ identities. Thus, unlike traditional *exchangeable* IPSs where the system is completely invariant under any permutation of the variables’ labels, particles are no longer exchangeable. In this regard, a powerful way to keep track of the identities of the particles and their connections is to use weighted graphs. More precisely, the labels can be encoded into a vertex set $V:=\{1,\ldots ,N\}$ and the heterogeneous interactions can be modelled by a family of interaction potentials $(W^{i,j})_{i,j=1}^N$ depending on which pair of particles $(i,j) \in E$ is being considered, where $E = V \times V$ is the set of edges. For example, one can consider the following IPS composed of particles $X_t^i \in \mathbb {R}^d$, with labels $i=1,\ldots , N$, evolving according to

Het-IPS $$\begin{aligned} {\left\{ \begin{array}{ll} \frac{\hbox {d}}{\hbox {d}t}X^i_t = -\frac{1}{N}\sum _{i\ne j}^N\kappa ({i,j})\nabla f(X_t^i-X_t^j), \\ X_0^i \in \mathbb {R}^d, \end{array}\right. } \end{aligned}$$

where the family of potentials $(W^{i,j})_{i,j=1}^N$ is given by $W^{i,j}:=\kappa ({i,j})f(X_t^i-X_t^j)$, and $\kappa :E \rightarrow \mathbb {R}$ encodes the differences in the interactions between the particles depending on their labels. Therefore, the vertices of this graph can be viewed as a collection of point masses that move in space, with the trajectory of each point mass being influenced by the rest. We refer to this approach as the *fixed-mass* approach, in contrast to the framework of this paper, where the positions of the masses are fixed, but the mass is allowed to evolve. We refer to the latter point of view as the *evolving-mass* approach, which is useful, for instance, in applications to data science and machine learning, since data have usually the form of point clouds, which constitute the sets of vertices. These vertices are, indeed, fixed, and the weighted edges can encode some measure of similarity, such as the distance between these points. In this context, graphs are a powerful tool to represent the data. In particular, dynamics on graphs can be used to detect meaningful clusters, as explained in Craig et al. (2021). Characterising clustering in our functional setting is a challenging question that remains an open problem. Let us stress that in this context the position is fixed (vertices), whereas the “mass” is moving.

The study of IPSs on heterogeneous graphs, i.e. not all-to-all with homogeneous weights, can be traced back to the series of seminal papers by Medvedev (2014a, 2014b), studying a nonlinear heat-equation on graphs and its continuum limit as the number of vertices (particles) goes to infinity. To the best of the authors’ knowledge, these contributions were the first to rigorously justify the use of continuum evolution equations, called the *graph limit*, to approximate dynamics on large dense graphs by using *graphons*, a notion of limiting graph for sequences of dense graphs (Lovász 2012). The graph limit describes the trajectory of an agent with a given label when the number of agents goes to infinity. The notion of graph limit in the context of IPSs has been studied extensively; we mention, e.g. Ayi and Duteil (2023), Gkogkas et al. (2023), Ben-Porat et al. (2024a, 2024b), Bouchairi et al. (2023), Favre et al. (2024) and Haskovec (2024). Continuity equations similar to (graph-CE) have been obtained in the study of the mean-field limit of IPSs with heterogeneous interactions, see, for example, Kaliuzhnyi-Verbovetskyi and Medvedev (2018), Chiba and Medvedev (2019a, 2019b), Kuehn (2020), Gkogkas and Kuehn (2022) and Kuehn and Xu (2022) for static graphs, although this list is not exhaustive. In the case of co-evolving graphs, we highlight the recent work (Gkogkas et al. 2025) on the Kuramoto model on a co-evolving graph, where the authors prove the mean-field limit and provide several examples both for dense and sparse graphs. These works rely on the recent theory of graph limits (Lovász 2012; Backhausz and Szegedy 2022). We also mention (Ayi et al. 2024; Paul and Trélat 2024) for recent reviews on the different approaches to defining the mean-field limit of heterogeneous IPS. In this sense, the first contribution in this manuscript can be viewed as an application of the ideas in  Paul and Trélat (2024), Section 5.2.3 on how to obtain a continuity equation starting from the solution of a nonlinear Euler equation, although our setting is not included in Paul and Trélat (2024). A common point in many of the aforementioned works and the one presented in this manuscript is the approach to obtain the well-posedness of the nonlinear continuity equation. Namely, the well-posedness is obtained by studying a family of characteristic equations parametrized by the vertices of the underlying graph extending the work of  Neunzert (1981). To the best of the author’s knowledge, the first application of this approach in the context of all-to-all graphs can be found in Lancellotti (2005) and, in the case of heterogeneous graphs, can be found in Kaliuzhnyi-Verbovetskyi and Medvedev (2018). Finally, we notice that in our framework a finite graph corresponds to $\mu _n$ being an atomic measure, where *n* is the number of vertices. As $n\rightarrow \infty$, we can obtain the so-called *graph limit*, giving rise to an integro-differential equation such as (Euler Co-NCL) or (Co-NCL) rather than a continuity-type equation, as in Esposito and Mikolás (2024). The graph-limit can be seen as an intermediate step in view of the connection between the Euler equation (infinite graphs) and the continuity equation via the $\mu$-monokinetic solution. A finite-graph approximation for (graph-CE) is an interesting question that is beyond the purposes of this manuscript and would deserve a deeper study.

The approach we use in Sect. 5 to study the long-time behaviour for Eq. (Euler Co-NCL) follows similar ideas to the ones in Bonnet et al. (2022), where the authors study the long-time behaviour of large systems of interacting agents on time-evolving graphs. Indeed, our result is linked to the problem of *consensus formation* for systems of *non-exchangeable* interacting particles. More precisely, this problem consists in determining whether there exists an element $x^\infty$ such that

$$ \lim _{t \rightarrow \infty } |x_i(t) - x^\infty |= 0, $$

for any agent $i \in \{1,\ldots ,N\}$ in an IPS. In our case, the evolving-mass approach, we obtain that $x^\infty = \frac{M}{\mu (K)}$, the uniform mass distribution. Consensus formation has received a vast amount of attention, see, for example, Caponigro et al. (2013, 2015), Dalmao and Mordecki (2011), Moreau (2005) and Olfati-Saber and Murray (2004) and the references therein. The literature on this topic is very extensive as techniques have to be adapted to the particular type of interaction and connectivity in each case. Our approach is close to the works that tackle the problem by obtaining *diameter* decay estimates. We obtain that the rate at which the “vertex with maximum mass” (“vertex with minimum mass”) loses (gains) mass depends on the connectivity of the system through the quantities $\eta _*$, the smallest connectivity for any vertex in the history of the co-evolving graph, and $\alpha '_*$, the smallest rate of outflow for the velocities. This is closely related to the *scrambling-coefficient* (Seneta 1979), obtained, for example, in Bonnet et al. (2022), a quantity which is positive if either a pair of agents is interacting or they follow a common third agent. Our condition is stronger, since we impose that the graph is strongly connected, i.e. $\eta _t >0$, for $t\ge 0$. This is due to the co-evolving nature of $\eta$, which is not present in most of the aforementioned works, where the connectivity of the topology is, at most, allowed to evolve in time, but not to depend on the dynamics of the agents, as in our case. Finding weaker conditions on $\eta$ yielding convergence to consensus could be achieved upon investigating different co-evolving dynamics.

The setting outlined in Definition 1.1 was introduced in Esposito et al. (2021) where the authors considered nonlocal dynamics on graphs driven by nonlocal interaction energies. Similar frameworks can be also found in García Trillos and Slepčev (2016, 2018) in static problems. The work Esposito et al. (2021), was extended in different directions. In Esposito et al. (2023), the authors studied a generalisation of the dynamics by looking at a wider class of interpolation functions $\Phi$ (the ones considered in this paper), while Esposito et al. (2021) focused on the upwind interpolation $\Phi _\textrm{upwind}$ (see Example 2.1), also studied in Chen et al. (2018). In Heinze et al. (2023b), the authors extended the framework of Esposito et al. (2021) to the case of two interacting species on graphs. They allow for nonlinear mobilities and consider a suitable modified *p*-Wasserstein quasi-metric for $p\in (1,\infty )$, generalising the metric defined in Esposito et al. (2021). The authors show that the multi-species dynamics also define a gradient flow of the corresponding interaction energy with respect to the generalised version of *p*-Wasserstein (quasi) metric on graphs. The study of nonlocal cross-interaction equations on graphs is continued by the same authors in Heinze et al. (2023a), where they investigate certain qualitative properties of solutions for finite graphs. Namely, the authors give conditions on the self-interaction and cross-interaction kernels leading to energy minimisers and give conditions for obtaining stationary states. Finally, they study the stability of stationary states, the formation of patterns, and the effect of the connectivity $\eta$ on the resulting dynamics. They carry out these efforts analytically for small graphs of up to four vertices and present numerical results for graphs of up to one hundred vertices. In Esposito et al. (2025), the authors studied a natural question posed in Esposito et al. (2021): does one recover the nonlocal interaction equation on $\mathbb {R}^d$ as the graph localises? In other words, as the range of connections between different vertices decreases, but the weight of each connecting edge increases, do the nonlocal dynamics on the graph converge to the dynamics on Euclidean space? In Esposito et al. (2025), the authors show that, as the graph localises, the obtained dynamics is the nonlocal interaction equation on Euclidean space, with a tensor mobility $\mathbb {T}$, depending on the geometry of the graph through the connectivity, $\eta$, and the base measure, $\mu$. Furthermore, the resulting PDE is a Riemannian gradient flow on $(\mathcal {P}_2(\mathbb {R}^d_{\mathbb {T}}),d_2)$, where $\mathbb {R}^d_{\mathbb {T}}$ is the *d*-dimensional Euclidean space endowed with a metric induced by $\mathbb {T}^{-1}$. This result establishes a link between the Finslerian gradient flow structure obtained in Esposito et al. (2021) and the Riemannian gradient flow obtained in the limiting Euclidean case; see Esposito et al. (2023) for an extension to systems of *N* nonlocal cross-interaction equations on graphs. In particular, graphs are space-discretisation alternative to tessellations. In this regard, for interesting discrete-to-continuum evolution problems, we refer the reader to Disser and Liero (2015), Forkert et al. (2022), Hraivoronska and Tse (2023) and Hraivoronska et al. (2024). Furthermore, we also mention (Gladbach et al. 2020, 2023) for discrete optimal transport distances and their convergence to their continuous counterparts. Manuscripts dealing with evolutions on finite graphs are Maas (2011), Chow et al. (2012) and Mielke (2011), where the concept of Wasserstein metric on finite graphs was introduced independently. Related nonlocal Wasserstein distances are studied in Slepčev and Warren (2023), as well as the recent (Warren 2024), which focuses on a class of nonlocal diffusion equations driven by relative entropy. We also mention (Bianchi et al. 2022), where the authors provide a well-posedness theory for generalised porous medium equation on infinite graphs.

### Structure of the Manuscript

The rest of the manuscript is structured as follows. In Sect. 2, we recall relevant definitions and results from Esposito et al. (2021, 2023) and Esposito and Mikolás (2024). Focusing on the case $\rho \ll \mu$, in Sect. 3 we study the well-posedness of Eq. (Euler Co-NCL), derived from (Co-NCL). In Sect. 4, we show how we obtain (graph-CE) from (Euler Co-NCL), prove its well-posedness and a stability estimate. We also discuss how this equation was obtained in an attempt to study the long-time behaviour of (Co-NCL). Considering the upwind interpolation, Sect.  5 focuses on the long-time behaviour of (Euler Co-NCL), and (graph-CE) as consequence, for pointwise monotonic velocities.

## Preliminaries

We shall focus on *co-evolving* graphs, denoted by $\mathcal {G}_t:= (\mu , \eta _t[\rho ])$, in which the weight function is allowed to change in time depending on the mass configuration, $\rho$, defined on the vertices.

Since it is useful in our analysis, we recall the notation and the results obtained in Esposito et al. (2023) and Esposito and Mikolás (2024). There, the unknown $\rho \in \mathcal {\mathcal {M}}_{\textrm{TV}}(\mathbb {R}^d)$ is a signed Radon measure with finite total variation and the set $\mathcal {\mathcal {M}}_{\textrm{TV}}$ is equipped with the total variation norm, defined using the dual product between $C_0(\mathbb {R}^d)$ and $\mathcal {M}(\mathbb {R}^d)$. Furthermore, since the continuity equation induces mass preserving dynamics that do not increase the total variation, we have $\rho$ that belongs to $\mathcal {\mathcal {M}}_{\textrm{TV}}^M(\mathbb {R}^d):=\left\{ \rho \in \mathcal {\mathcal {M}}_{\textrm{TV}}(\mathbb {R}^d):|\rho |(\mathbb {R}^d)\le M\right\}$. We shall deal with dynamics on a non-Euclidean setting; hence, we recall the notion of gradient and divergence on co-evolving graphs, as in Esposito et al. (2021).

### Definition 2.1

(*Nonlocal gradient and divergence*) For any $\phi : \mathbb {R}^d \rightarrow \mathbb {R}$, we define its nonlocal gradient ${\overline{\nabla }} \phi : {\mathbb {R}^{2d}_{\!\scriptscriptstyle \diagup }}\rightarrow \mathbb {R}$ by

$$ {\overline{\nabla }} \phi (x, y)=\phi (y)-\phi (x), \quad \text{ for } \text{ all } (x, y) \in {\mathbb {R}^{2d}_{\!\scriptscriptstyle \diagup }}. $$

For any Radon measure $\boldsymbol{j} \in \mathcal {M}({\mathbb {R}^{2d}_{\!\scriptscriptstyle \diagup }})$, its nonlocal divergence ${\overline{\nabla }} \cdot \boldsymbol{j} \in \mathcal {M}(\mathbb {R}^d)$ is defined as the adjoint of ${\overline{\nabla }}$, i.e. for any $\phi : \mathbb {R}^d \rightarrow \mathbb {R}$ in $C_0(\mathbb {R}^d)$, there holds

$$ \begin{aligned} \int _{\mathbb {R}^d} \phi \textrm{d} {\overline{\nabla }} \cdot \boldsymbol{j}&=-\frac{1}{2} \iint _{\mathbb {R}^{2d}_{\!\scriptscriptstyle \diagup }}{\overline{\nabla }} \phi (x, y)\textrm{d} \boldsymbol{j}(x, y) \\&=\frac{1}{2} \int _{\mathbb {R}^d} \phi (x) \int _{\mathbb {R}^d \backslash \{x\}} (\textrm{d} \boldsymbol{j}(x, y)-\textrm{d} \boldsymbol{j}(y, x)). \end{aligned} $$

In particular, for $\boldsymbol{j}$ antisymmetric, that is, $\boldsymbol{j} \in \mathcal {M}({\mathbb {R}^{2d}_{\!\scriptscriptstyle \diagup }})$ and $\textrm{d}\boldsymbol{j}(x,y)=-\textrm{d}\boldsymbol{j}(y,x)$, denoted $\boldsymbol{j} \in$ $\mathcal {M}^{\textrm{as}}({\mathbb {R}^{2d}_{\!\scriptscriptstyle \diagup }})$, we have

$$ \int _{\mathbb {R}^d} \phi \textrm{d} {\overline{\nabla }} \cdot \boldsymbol{j}=\iint _{\mathbb {R}^{2d}_{\!\scriptscriptstyle \diagup }}\phi (x)\textrm{d} \boldsymbol{j}(x, y). $$

The solutions to the nonlocal continuity equations are curves on a time interval [0, *T*], with $T>0$, and we denote by ${{\,\textrm{AC}\,}}([0,T];\mathcal {\mathcal {M}}_{\textrm{TV}}(\mathbb {R}^d))$ the set of curves from [0, *T*] to $\mathcal {\mathcal {M}}_{\textrm{TV}}(\mathbb {R}^d)$ absolutely continuous with respect to the ${{\,\mathrm{{TV}}\,}}$ norm, that is, the set of curves $\rho :[0,T] \rightarrow \mathcal {\mathcal {M}}_{\textrm{TV}}(\mathbb {R}^d)$ such that there exists $m\in L^1([0,T])$ with

$$\begin{aligned} \Vert \rho _t-\rho _s \Vert _{{{\,\mathrm{{TV}}\,}}} \le \int _s^t m(r) \textrm{d}r, \qquad \text {for all } 0\le s< t\le T. \end{aligned}$$

The definition of the flux requires further considerations in the graph setting since the mass is a vertex-based quantity, while the velocity is an edge-based quantity. It is necessary to interpolate the mass at two given adjacent vertices to create an edge-based quantity according to an admissible flux interpolation. The definition of admissible flux interpolations and fluxes follows Esposito et al. (2023) and Esposito and Mikolás (2024).

### Definition 2.2

(*Admissible flux interpolation*) A measurable function $\Phi : \mathbb {R}^3 \rightarrow \mathbb {R}$ is called an admissible flux interpolation, provided that the following conditions hold:

(i) $$\Phi $$ satisfies
  $$ \Phi (0,0; v)=\Phi (a, b; 0)=0, \quad \text{ for } \text{ all } a, b, v \in \mathbb {R}; $$
(ii) $\Phi$ is argument-wise Lipschitz in the sense that, for some $L_{\Phi }>0$, any a, $b, c, d, v, w \in$ $\mathbb {R}$, it holds
  $$ \begin{aligned} |\Phi (a, b; w)-\Phi (a, b; v)|&\le L_{\Phi }(|a|+|b|)|w-v|; \\ |\Phi (a, b; v)-\Phi (c, d; v)|&\le L_{\Phi }(|a-c|+|b-d|)|v|; \end{aligned} $$
(iii) $\Phi$ is positively one-homogeneous in its first and second arguments, that is, for all $\alpha >0$ and $(a, b, w) \in \mathbb {R}^3$, it holds
  $$ \Phi (\alpha a, \alpha b; w)=\alpha \Phi (a, b; w). $$

### Example 2.1

A particularly useful interpolation, especially for potential vector fields, is the upwind interpolation, used, e.g. in Esposito et al. (2021),

$$ \Phi _{\text{ upwind } }(a, b; w)=a w_{+}-b w_{-}, \qquad \text{ for } (a,b,w)\in \mathbb {R}^3, $$

where $w_+:= \max \{0,w\}$ and $w_-:=(-w)_{+}$ are the positive and negative parts of $w\in \mathbb {R}$, respectively. Mean multipliers represent another example, given by

$$ \Phi _{\text{ prod } }(a, b; w)=\phi (a, b)w, \qquad \text{ for } (a,b,w)\in \mathbb {R}^3, $$

where common choices for $\phi$ include: $\phi (a,b) = \frac{a+b}{2}$, or $\phi (a,b) = \max \{a,b\}$.

An admissible flux is defined as follows.

### Definition 2.3

(*Admissible flux*) Let $\Phi$ be an admissible flux interpolation, and let $\rho \in \mathcal {M}_{\textrm{TV}}(\mathbb {R}^d)$, $w \in \mathcal {V}^{\textrm{as}}({\mathbb {R}^{2d}_{\!\scriptscriptstyle \diagup }}):=\left\{ v: {\mathbb {R}^{2d}_{\!\scriptscriptstyle \diagup }}\rightarrow \mathbb {R}| v(x,y)=-v(y,x)\right\}$, and $\eta :{\mathbb {R}^{2d}_{\!\scriptscriptstyle \diagup }}\rightarrow \mathbb {R}$ measurable. Furthermore, take a reference measure $\lambda \in \mathcal {M}^+(\mathbb {R}^{2 d})$ such that $\rho \otimes \mu , \mu \otimes \rho \ll \lambda$. Then, the admissible flux $F^{\Phi }[\mu , \eta ; \rho , w] \in \mathcal {M}({\mathbb {R}^{2d}_{\!\scriptscriptstyle \diagup }})$ at $(\rho , w)$ is defined by

$$ \textrm{d} F^{\Phi }[\mu , \eta ; \rho , w]=\Phi \left( \frac{\textrm{d}(\rho \otimes \mu )}{\textrm{d} \lambda }, \frac{\textrm{d}(\mu \otimes \rho )}{\textrm{d} \lambda }; w\right) \eta \, \textrm{d} \lambda . $$

The one-homogeneity of $\Phi$ implies that the definition does not depend of the choice of $\lambda$, as long as the absolute continuity requirement is considered, cf. (Esposito et al. 2023, Remark 2.6). The *co-evolving graphs*, $\mathcal {G}_t$, are determined by the following initial value problem introduced in Esposito et al. (2023)

Co-NCL $$\begin{aligned} \begin{aligned} \partial _t \rho _t&= - \overline{\nabla }\cdot F^\Phi [\mu , \eta _t ; \rho _t,V_t[\rho _t]], \\ \partial _t \eta _t&= \omega _t[\rho _t] -\eta _t, \end{aligned} \end{aligned}$$

for given initial data $\rho ^0 \in \mathcal {\mathcal {M}}_{\textrm{TV}}^M(\mathbb {R}^d)$ and $\eta ^0\in C_{b}({\mathbb {R}^{2d}_{\!\scriptscriptstyle \diagup }})$. Focusing on the dynamics for $\eta$, we consider $\omega :[0,T]\times \mathcal {\mathcal {M}}_{\textrm{TV}}(\mathbb {R}^d) \times {\mathbb {R}^{2d}_{\!\scriptscriptstyle \diagup }}\rightarrow \mathbb {R}$ satisfying the following assumptions:

$({\boldsymbol{\omega }}1)$: the map $(x,y)\in {\mathbb {R}^{2d}_{\!\scriptscriptstyle \diagup }}\mapsto \omega _t[\cdot ](x,y)$ is continuous and $(t\mapsto \omega _t[\cdot ](\cdot ,\cdot ))\in L^1([0,T])$;
$({\boldsymbol{\omega }}2)$: for any $\rho , \sigma \in \mathcal {\mathcal {M}}_{\textrm{TV}}(\mathbb {R}^d)$ there exists a constant $L_\omega \ge 0$ such that
  $$\begin{aligned} \sup _{t \in [0,T]}\sup _{x,y \in {\mathbb {R}^{2d}_{\!\scriptscriptstyle \diagup }}} | \omega _t[\sigma ](x,y) - \omega _t[\rho ](x,y)|&\le L_\omega \Vert \sigma - \rho \Vert _{TV}; \end{aligned}$$
$({\boldsymbol{\omega }}3)$: $\omega$ is bounded, that is, there exists a constant $C_\omega >0$ such that
  $$\begin{aligned} \sup _{t \in [0,T]}\sup _{\rho \in \mathcal {\mathcal {M}}_{\textrm{TV}}(\mathbb {R}^d)}\sup _{x,y \in {\mathbb {R}^{2d}_{\!\scriptscriptstyle \diagup }}}\big |\omega _t[\rho ](x,y)\big | \le C_\omega . \end{aligned}$$
$({\boldsymbol{\omega }}4)$: The map $(x,y) \mapsto \omega _\cdot [\cdot ](x,y)$ is symmetric.

These requirements are needed to ensure the well-posedness of (Co-NCL) and to be consistent with meaningful examples for the weight function $\eta$, see Remark 2.1. We observe that assumption $({\boldsymbol{\omega }}4)$ is not required in Esposito and Mikolás (2024) since it is not needed for the well-posedness of (Co-NCL). However, in what follows, we will require $\eta$ to be symmetric, and hence, $({\boldsymbol{\omega }}4)$ is necessary.

### Remark 2.1

The weight function is assumed to be continuous and bounded on its domain according to typical choices in applications to machine learning. We mention, for instance, geometric graphs where the edge-weight is a function of the distance, cf. García Trillos and Slepčev (2016) and García Trillos et al. (2020) and the references therein. This is also consistent with the setting developed in Esposito et al. (2021, 2023) and Esposito and Mikolás (2024).

A solution to (Co-NCL) is understood as follows.

### Definition 2.4

(*Solution to the initial value problem* (Co-NCL)) Given an admissible flux interpolation $\Phi$, a velocity field $V:[0,T]\times \mathcal {\mathcal {M}}_{\textrm{TV}}(\mathbb {R}^d)\rightarrow \mathcal {V}^{as}({\mathbb {R}^{2d}_{\!\scriptscriptstyle \diagup }})$, and function $\omega :[0,T]\times \mathcal {\mathcal {M}}_{\textrm{TV}}(\mathbb {R}^d)\times {\mathbb {R}^{2d}_{\!\scriptscriptstyle \diagup }}\rightarrow \mathbb {R}$, a pair $(\rho , \eta ): [0,T] \rightarrow \mathcal {\mathcal {M}}_{\textrm{TV}}(\mathbb {R}^d)\times C_{b}({\mathbb {R}^{2d}_{\!\scriptscriptstyle \diagup }})$ is a solution to the initial value problem (Co-NCL) if

(i) $$\rho \in AC([0,T], \mathcal {\mathcal {M}}_{\textrm{TV}}(\mathbb {R}^d)),\ \eta \in AC([0,T], C_{b}({\mathbb {R}^{2d}_{\!\scriptscriptstyle \diagup }}))$$;
(ii) the maps $t \mapsto \langle \varphi ,\overline{\nabla }\cdot F^\Phi [\mu , \eta _t; \rho _t,V_t[\rho _t]]\rangle$ and $t \mapsto \omega _t[\rho _t] -\eta _t$ belong to $L^1([0,T])$, for any $\varphi \in C_0(\mathbb {R}^d)$;
(iii) for a.e. $t \in [0,T]$, every $(x,y)\in {\mathbb {R}^{2d}_{\!\scriptscriptstyle \diagup }}$, for any $\varphi \in C_0(\mathbb {R}^d)$, the following conditions hold
  2.1 $$\begin{aligned} \int _{\mathbb {R}^d}\varphi (x)\textrm{d}\rho _t(x)&= \int _{\mathbb {R}^d}\varphi (x)\textrm{d}\rho _0(x)\nonumber \\&\quad + \frac{1}{2} \int _{0}^t \iint _{{\mathbb {R}^{2d}_{\!\scriptscriptstyle \diagup }}}\overline{\nabla }\varphi (x,y)\textrm{d}F^\Phi [\mu ,\eta _s,\rho _s;V_s[\rho _s]](x,y) \textrm{d}s\ ,\end{aligned}$$
  2.2 $$\begin{aligned} \eta _t(x,y)&= \eta _0(x,y) + \int _0^t \left( \omega _s[\rho _s](x,y) -\eta _s(x,y)\right) \,\textrm{d}s. \end{aligned}$$

We recall the main results needed to ensure well-posedness of (Co-NCL) from Esposito and Mikolás (2024).

### Theorem 2.1

(Existence and uniqueness for (Co-NCL)) Let $V:[0,T]\times \mathcal {\mathcal {M}}_{\textrm{TV}}^M(\mathbb {R}^d)\rightarrow \mathcal {V}^{as}({\mathbb {R}^{2d}_{\!\scriptscriptstyle \diagup }})$ satisfy the uniform compressibility assumption

2.3 $$\begin{aligned} \sup _{t \in [0, T]} \sup _{\rho \in \mathcal {M}_{TV}^M(\mathbb {R}^d)} \sup _{x \in \mathbb {R}^d} \int _{\mathbb {R}^d \backslash \{x\}}\left| V_t[\rho ](x, y)\right| \textrm{d} \mu (y) \le C_V\ , \end{aligned}$$

for $C_V>0$. Assume there is a constant $L_V > 0$ such that, for all $t \in [0,T]$ and all $\rho , \sigma \in \mathcal {M}_{TV}^M(\mathbb {R}^d)$:

2.4 $$\begin{aligned} \sup _{x \in \mathbb {R}^d} \int _{\mathbb {R}^d \backslash \{x\}}\left| V_t[\rho ](x, y)-V_t[\sigma ](x, y)\right| \textrm{d} \mu (y) \le L_V\Vert \rho -\sigma \Vert _{\textrm{TV}} \ . \end{aligned}$$

Let $\omega :[0,T]\times \mathcal {\mathcal {M}}_{\textrm{TV}}(\mathbb {R}^d)\times {\mathbb {R}^{2d}_{\!\scriptscriptstyle \diagup }}\rightarrow \mathbb {R}$ satisfy $({\boldsymbol{\omega }}1)$–$({\boldsymbol{\omega }}3)$. Then, there exists a unique solution $(\rho ,\eta )$ to (Co-NCL) such that $(\rho _0,\eta _0)=(\rho ^0,\eta ^0)$.

### Proof

We refer the reader to Esposito and Mikolás (2024), Lemma 3.3 and Esposito and Mikolás (2024), Theorem 3.6. $\square$

As shown in Esposito and Mikolás (2024), Proposition 2.8 (Co-NCL) preserves mass, i.e. $\rho _t(\mathbb {R}^d) = \rho _0(\mathbb {R}^d)$ for $t\in [0,T]$. In addition to Theorem 2.1, in Esposito and Mikolás (2024), the authors analyse different time scales for (Co-NCL) leading either to static graphs or to co-evolving graphs with $\eta =\eta (\rho )$. Furthermore, they study a stability result with respect to the base measure $\mu$, also known as *graph limit* in case $\mu _n\overset{*}{\rightharpoonup }\mu$ and $\mu _n$ is a sequence of atomic measures (describing finite graphs). In the remainder of this article, for any curve $\gamma \in C([0,T],S)$, for some normed space $(S, \Vert \cdot \Vert _S)$, we will write

$$ \Vert \gamma \Vert _{\infty ,S}:= \sup _{t \in [0,T]}\Vert \gamma _t \Vert _{S}. $$

We denote by $\Vert \cdot \Vert _\infty$ the usual sup-norm when this does not create confusion. For the reader’s convenience, we postpone to the corresponding section the preliminary results we shall use for the long-time behaviour in Sect. 5.2.

## The Euler Equation for (Co-NCL)

We focus on the case when $\mu$ defines the set of all possible vertices so that it makes sense to consider $\rho \ll \mu$. In particular, for any $t\in [0,T]$, $\rho _t: = r_t\textrm{d}\mu$, for a curve $r \in C([0,T],L^2_\mu (\mathbb {R}^d))$, and $\mu \in \mathcal {P}_2(\mathbb {R}^d)$. In this setting, the nonlocal continuity equation in (Co-NCL) can be rewritten so that it can be interpreted as a nonlinear Euler equation. We observe that in this set-up there is an interesting comparison between the study carried out in Esposito et al. (2021, 2023), Esposito and Mikolás (2024) and the recent developments on heterogeneous interacting particle systems, e.g. in Paul and Trélat (2024) and Ayi and Duteil (2024). Indeed, since $\rho \ll \mu$, we set $\rho :=r\textrm{d}\mu$, with *r* being the Radon–Nikodym derivative of $\rho$ with respect to $\mu$. By choosing $\lambda := \mu \otimes \mu$ in Definition 2.3, we write the flux as

$$ \textrm{d}F^{\Phi }[\mu , \eta ; \rho , w](x, y)=\Phi (r(x), r(y); w(x, y))\eta (x,y) \textrm{d}(\mu \otimes \mu )(x, y), $$

for $\ w \in \mathcal {V}^{\text {as}}({\mathbb {R}^{2d}_{\!\scriptscriptstyle \diagup }})$, and $(x, y) \in {\mathbb {R}^{2d}_{\!\scriptscriptstyle \diagup }}$, so that $\Phi$ is function of the density, *r*, w.r.t. $\mu$. Furthermore, if we assume that $\Phi$ is jointly antisymmetric, that is, $\Phi (a, b;-v)=-\Phi (b, a; v)$ for any $a, b, v \in \mathbb {R}$, and $\eta$ symmetric, the nonlocal divergence of $F^{\Phi }[\mu , \eta ; \rho , V[\rho ]]$ is given by

3.1 $$\begin{aligned} \overline{\nabla }\cdot F^\Phi [\mu , \eta ; \rho , V[\rho ]](x)=\int _{\mathbb {R}^d \backslash \{x\}} \Phi (r(x), r(y) ; V[\rho ](x, y)) \eta (x, y) \textrm{d} \mu (y) \ , \end{aligned}$$

for $\mu \text{-a.e. } x \in \mathbb {R}^d$. In view of this consideration, we shall consider the system

Euler Co-NCL $$\begin{aligned} \begin{aligned}&\partial _t r_t(x) = - \int _{\mathbb {R}^d\backslash \{x\}}\Phi (r_t(x),r_t(y); V_t[r](x,y))\eta _t(x,y)\textrm{d}\mu (y)\\&\partial _t \eta _t = \omega _t[r] -\eta _t, \end{aligned} \end{aligned}$$

for given initial data $r^0 \in L^2_\mu (\mathbb {R}^d)$ and $\eta ^0\in C_{b}({\mathbb {R}^{2d}_{\!\scriptscriptstyle \diagup }})$ and symmetric. By slight abuse of notation, both *V* and $\omega$ will be considered as functions of the density *r* to be consistent in the subsequent analysis. Solutions of (Euler Co-NCL) are defined as follows, either in $L^2_\mu (\mathbb {R}^d)$ or in $L^\infty _\mu (\mathbb {R}^d)$ since we shall need both settings.

### Definition 3.1

(*Solution to (Euler Co-NCL)*) Let $p=2,\infty$. Given a jointly antisymmetric admissible flux interpolation $\Phi$, a velocity field $V:[0,T]\times L^p_\mu (\mathbb {R}^d) \rightarrow \mathcal {V}^{as}({\mathbb {R}^{2d}_{\!\scriptscriptstyle \diagup }})$, and a function $\omega :[0,T]\times L^p_\mu (\mathbb {R}^d)\times {\mathbb {R}^{2d}_{\!\scriptscriptstyle \diagup }}\rightarrow \mathbb {R}$, a pair $(r, \eta ): [0,T] \rightarrow L^p_\mu (\mathbb {R}^d)\times C_{b}({\mathbb {R}^{2d}_{\!\scriptscriptstyle \diagup }})$ is a solution (Euler Co-NCL) with initial datum $(r_0,\eta _0)\in L^p_\mu (\mathbb {R}^d)\times C_b({\mathbb {R}^{2d}_{\!\scriptscriptstyle \diagup }})$ if it satisfies the following properties:

1. the maps $t \mapsto \int _{\mathbb {R}^d\backslash \{x\}}\Phi (r_t(x),r_t(y); V_t[r](x,y))\eta _t(x,y)\textrm{d}\mu (y)$ and $t \mapsto \omega _t[\rho _t] -\eta _t$ belong to $L^1([0,T])$;
2. for $\mu$-a.e. $x \in \mathbb {R}^d$, $t \in [0,T]$, $(x,y)\in {\mathbb {R}^{2d}_{\!\scriptscriptstyle \diagup }}$, it holds
  3.2a $$\begin{aligned} r_t(x)&= r_0(x) - \int _{0}^t \int _{\mathbb {R}^d\backslash \{x\}}\Phi (r_s(x),r_s(y); V_s[r](x,y))\eta _s(x,y)\textrm{d}\mu (y)\textrm{d}s \end{aligned}$$
  3.2b $$\begin{aligned} \eta _t(x,y)&= \eta _0(x,y) + \int _0^t \left( \omega _s[r](x,y) -\eta _s(x,y)\right) \,\textrm{d}s. \end{aligned}$$
3. $$r \in AC([0,T], L^p_\mu (\mathbb {R}^d)),\ \eta \in AC([0,T], C_{b}({\mathbb {R}^{2d}_{\!\scriptscriptstyle \diagup }}))$$.

Possible examples, in the $L^2_\mu$ setting, for the functions *V* and $\omega$ are given by

3.3 $$\begin{aligned} \omega _t[r](x,y)&= \int _{\mathbb {R}^d}W(t,x,y,z) r(z)\textrm{d}\mu (z), \end{aligned}$$

3.4 $$\begin{aligned} V_t[r](x,y)&= - \overline{\nabla }(K*\rho _t)(x,y)= -\int _{\mathbb {R}^d} (K(y,z)- K(x,z))r_t(z) \textrm{d}\mu (z), \end{aligned}$$

where for $(x,y) \in {\mathbb {R}^{2d}_{\!\scriptscriptstyle \diagup }}$, the map $t\mapsto W(t,\cdot ,\cdot ,\cdot ) \in L^1([0,T])$, and $W \in C_b([0,T]\times {\mathbb {R}^{2d}_{\!\scriptscriptstyle \diagup }}\times \mathbb {R}^d)$, $K \in C_b(\mathbb {R}^2)$ and the velocity defined in (3.4) is related to the nonlocal interaction energy $\mathcal {E}(\rho )=(1/2)\iint K(x,y)\textrm{d}\rho (x)\textrm{d}\rho (y)$.

For $p=2,\infty$, we obtain well-posedness of (Euler Co-NCL) by exploiting the Banach fixed-point theorem in the space $C([0,T], {L^p_\mu (\mathbb {R}^d)})\times C([0,T], C_b({\mathbb {R}^{2d}_{\!\scriptscriptstyle \diagup }}))$ equipped with the distance

$$ \tilde{d}_\infty ((f^1,g^1), (f^2,g^2)):= \Vert f^1-f^2 \Vert _{\infty , {L^p_\mu (\mathbb {R}^d)}} + \Vert g^1-g^2 \Vert _{\infty , C_b({\mathbb {R}^{2d}_{\!\scriptscriptstyle \diagup }})}, $$

$$\begin{aligned} \Vert f^1-f^2 \Vert _{\infty , {L^p_\mu (\mathbb {R}^d)}}&:= \sup _{t \in [0,T]} \Vert f^1_t-f^2_t \Vert _{{L^p_\mu (\mathbb {R}^d)}} \text{ and } \Vert g^1-g^2 \Vert _{\infty , C_b({\mathbb {R}^{2d}_{\!\scriptscriptstyle \diagup }})}\\&:= \sup _{t \in [0,T]} \Vert g^1_t-g^2_t \Vert _{C_b({\mathbb {R}^{2d}_{\!\scriptscriptstyle \diagup }})} \ . \end{aligned}$$

In this section, we fix $p=2$ and prove *a priori* properties for (Euler Co-NCL) useful to define the solution maps.

### Proposition 3.1

Let $\Phi$ be a jointly antisymmetric admissible flux interpolation, $r_0 \in L^2_{\mu }(\mathbb {R}^d)$, $\eta _0\in C_b({\mathbb {R}^{2d}_{\!\scriptscriptstyle \diagup }})$ and symmetric. Assume $\omega :[0,T]\times L^2_\mu (\mathbb {R}^d)\times {\mathbb {R}^{2d}_{\!\scriptscriptstyle \diagup }}\rightarrow \mathbb {R}$ is such that the map $(x,y)\in {\mathbb {R}^{2d}_{\!\scriptscriptstyle \diagup }}\mapsto \omega _t[\cdot ](x,y)$ is continuous and that for a constant $C_\omega >0$

$$ \int _0^T \sup _{r \in L^2_\mu (\mathbb {R}^d)}\sup _{(x,y) \in {\mathbb {R}^{2d}_{\!\scriptscriptstyle \diagup }}}|\omega _s[r](x,y)| \textrm{d}s\le C_\omega . $$

Suppose that $V:[0, T]\times L^2_\mu (\mathbb {R}^d) \rightarrow \mathcal {V}^{\textrm{as}}({\mathbb {R}^{2d}_{\!\scriptscriptstyle \diagup }})$ satisfies

3.5 $$\begin{aligned} \int _0^T \sup _{r \in L^2_\mu (\mathbb {R}^d)}\sup _{x \in \mathbb {R}^d} \int _{\mathbb {R}^d\backslash \{x\}}|V_t[r](x,y)|^2\textrm{d}\mu (y)\textrm{d}t \le C_V, \end{aligned}$$

for some $C_V>0$. For a pair $(r,\eta ):[0,T]\rightarrow L^2_\mu (\mathbb {R}^d)\times C_b({\mathbb {R}^{2d}_{\!\scriptscriptstyle \diagup }})$ satisfying () the following properties hold.

1. For $\mu$-a.e. $x \in \mathbb {R}^d$ and every $(x,y)\in {\mathbb {R}^{2d}_{\!\scriptscriptstyle \diagup }}$, the maps satisfy
  $$\begin{aligned}&t \mapsto \int _{\mathbb {R}^d\backslash \{x\}}\Phi (r_t(x),r_t(y); V_t[r](x,y))\eta _t(x,x)\textrm{d}\mu (y) \in L^1([0,T]), \\&t\mapsto \omega _t[r](x,y) - \eta _t(x,y) \in L^1([0,T]) , \end{aligned}$$
  and $$r \in L^\infty ([0,T];L^2_\mu (\mathbb {R}^d)), \eta \in L^\infty ([0,T],C_b({\mathbb {R}^{2d}_{\!\scriptscriptstyle \diagup }}))$$.
2. $$r \in AC([0,T];L^2_\mu (\mathbb {R}^d))$$ and $$\eta \in AC([0,T];C_b({\mathbb {R}^{2d}_{\!\scriptscriptstyle \diagup }}))$$.

### Proof

We obtain $\eta \in L^\infty ([0,T]; C_b({\mathbb {R}^{2d}_{\!\scriptscriptstyle \diagup }}))$ in an analogous way to Esposito and Mikolás (2024), Proposition 2.8 using (2.2), with

3.6 $$\begin{aligned} \Vert \eta _t\Vert _{\infty }\le \left( \Vert \eta _0\Vert _{\infty } + C_\omega \right) e^{T}\ , \end{aligned}$$

and note that the integrability of $t\mapsto \omega _t[r](x,y) - \eta _t(x,y)$ directly follows. As for *r*, for $\mu$-a.e. $x\in \mathbb {R}^d$, by using (i), (ii) in Definition 2.2, boundedness of $\eta$, and (3.5) we have

$$\begin{aligned} |r_t(x)|&\le |r_0(x)| + \int _{0}^t\int _{\mathbb {R}^d\backslash \{x\}}\left| \Phi (r_s(x),r_s(y); V_s[r](x,y))\eta _s(x,y)\right| \textrm{d}\mu (y)\textrm{d}s \\&\le |r_0(x)| +L_\Phi \Vert \eta \Vert _{\infty , C_b({\mathbb {R}^{2d}_{\!\scriptscriptstyle \diagup }})} \int _{0}^t\int _{\mathbb {R}^d\backslash \{x\}}\left| V_s[r](x,y)\right| (|r_s(x)| + |r_s(y)|)\textrm{d}\mu (y)\textrm{d}s \\&\le |r_0(x)|\! +\!L_\Phi \Vert \eta \Vert _{\infty , C_b({\mathbb {R}^{2d}_{\!\scriptscriptstyle \diagup }})}\! \int _{0}^t\!\left( \sup _{r\in L^2_\mu (\mathbb {R}^d)}\sup _{x \in \mathbb {R}^d}\int _{\mathbb {R}^d\backslash \{x\}}\left| V_s[r](x,y)\right| ^2\textrm{d}\mu (y)\!\right) ^{\frac{1}{2}}\!\! \\&\quad \times \left( \!\int _{\mathbb {R}^d\backslash \{x\}}\!\!\!(|r_s(x)|+ |r_s(y)|)^2\textrm{d}\mu (y)\right) ^{\frac{1}{2}}\!\!\!\! \textrm{d}s \\&\le |r_0(x)| + L_\Phi \Vert \eta \Vert _{\infty , C_b({\mathbb {R}^{2d}_{\!\scriptscriptstyle \diagup }})} \int _{0}^t\bar{v}_s^{1/2}({\Vert r_s \Vert _{L^2_\mu (\mathbb {R}^d)}} + |r_s(x)|) \textrm{d}s\ , \end{aligned}$$

where we used the Cauchy–Schwarz and Minkowski inequalities, $\mu (\mathbb {R}^d)=1$, and we set

3.7 $$\begin{aligned} \bar{v}_t:=\sup _{r\in L^2_\mu (\mathbb {R}^d)}\sup _{x \in \mathbb {R}^d}\left( \int _{\mathbb {R}^d\backslash \{x\}}|V_t[r](x,y)|^2 \textrm{d}\mu (y)\right) \ . \end{aligned}$$

An application of Minkowski’s inequality and (Folland 1999, Theorem 6.19 a.) yields

$$ \Vert r_t \Vert _{L^2_\mu (\mathbb {R}^d)} \le \Vert r_0 \Vert _{L^2_\mu (\mathbb {R}^d)} + L_\Phi \Vert \eta \Vert _{\infty , C_b({\mathbb {R}^{2d}_{\!\scriptscriptstyle \diagup }})} 2\int _{0}^t\bar{v}_s^{1/2}{\Vert r_s \Vert _{L^2_\mu (\mathbb {R}^d)}} \textrm{d}s . $$

Then, an application of Grönwall’s inequality yield, for any $t\in [0,T]$,

3.8 $$\begin{aligned} \Vert r_t \Vert _{L^2_\mu (\mathbb {R}^d)} \le \Vert r_0 \Vert _{L^2_\mu (\mathbb {R}^d)}e^{2L_\Phi \Vert \eta \Vert _\infty C_V}, \end{aligned}$$

thus $r \in L^{\infty }([0,T];L^2_\mu (\mathbb {R}^d))$. The integrability of the nonlocal divergence can be proven by a further application of Gronwall inequality. More precisely, by the previous argument we know that, for $\mu$-a.e. $x\in \mathbb {R}^d$,

$$\begin{aligned} |r_t(x)|&\le |r_0(x)|+\int _{0}^t\int _{\mathbb {R}^d\backslash \{x\}}\left| \Phi (r_t(x),r_t(y); V_s[r_s](x,y))\eta _s(x,y)\right| \textrm{d}\mu (y)\textrm{d}s \\&\le |r_0(x)|\\&\quad + L_\Phi \Vert \eta \Vert _{\infty , C_b({\mathbb {R}^{2d}_{\!\scriptscriptstyle \diagup }})}\left( \Vert r_0 \Vert _{L^2_\mu (\mathbb {R}^d)}e^{2L_\Phi \Vert \eta \Vert _\infty C_V} \int _{0}^t\bar{v}_s^{1/2}\textrm{d}s+ \int _{0}^t\bar{v}_s^{1/2}|r_s(x)| \textrm{d}s\right) \\&\le |r_0(x)| + L_\Phi \Vert \eta \Vert _{\infty , C_b({\mathbb {R}^{2d}_{\!\scriptscriptstyle \diagup }})}\left( \Vert r_0 \Vert _{L^2_\mu (\mathbb {R}^d)}e^{2L_\Phi \Vert \eta \Vert _\infty C_V}\left( t\int _{0}^t\bar{v}_s\textrm{d}s \right) ^{1/2}\right. \\&\quad \left. + \int _{0}^t\bar{v}_s^{1/2}|r_s(x)| \textrm{d}s\right) \\&\le |r_0(x)|+\bar{C}\sqrt{t}+L_\Phi \Vert \eta \Vert _{\infty , C_b({\mathbb {R}^{2d}_{\!\scriptscriptstyle \diagup }})}\int _{0}^t\bar{v}_s^{1/2}|r_s(x)| \textrm{d}s, \end{aligned}$$

for $\bar{C}=L_\Phi \Vert \eta \Vert _{\infty , C_b({\mathbb {R}^{2d}_{\!\scriptscriptstyle \diagup }})}\Vert r_0 \Vert _{L^2_\mu (\mathbb {R}^d)}e^{2L_\Phi \Vert \eta \Vert _\infty C_V}C_V^{1/2}$. Then, for any $t\in [0,T]$ and $\mu$-a.e. $x\in \mathbb {R}^d$,

$$\begin{aligned} |r_t(x)|&\le (|r_0(x)|+\bar{C}\sqrt{t})\exp \left( L_\Phi \Vert \eta \Vert _{\infty , C_b({\mathbb {R}^{2d}_{\!\scriptscriptstyle \diagup }})}\int _0^t\bar{v_s}^{1/2} \textrm{d}s\right) \\&\le (|r_0(x)|+\bar{C}\sqrt{t})e^{L_\Phi \Vert \eta \Vert _{\infty , C_b({\mathbb {R}^{2d}_{\!\scriptscriptstyle \diagup }})}\sqrt{t}\sqrt{C_V}}, \end{aligned}$$

from which we infer time integrability of the nonlocal divergence, since we can bound ${\Vert r_s \Vert _{L^2_\mu (\mathbb {R}^d)}} + |r_s(x)|$ on [0, *T*]. Part (2) is consequence of the time integrability and (). $\square$

We proceed with proving well-posedness of (Euler Co-NCL). Fix $r_0\in L^2_\mu (\mathbb {R}^d),\ \eta _0 \in C_b({\mathbb {R}^{2d}_{\!\scriptscriptstyle \diagup }})$ for $\eta _0$ symmetric, and assume $\Phi$ is a jointly antisymmetric admissible flux interpolation as in Definition 2.2. We consider

$$ \mathcal{A}\mathcal{C}_T:=AC([0,T]; L^2_{\mu }(\mathbb {R}^d)) \times AC([0,T]; C_b({\mathbb {R}^{2d}_{\!\scriptscriptstyle \diagup }})), $$

and equip it with the distance $\tilde{d}_\infty$. We define the solution map $\mathcal {S}:= (\mathcal {S}_V,\mathcal {S}_\omega ): \mathcal{A}\mathcal{C}_T\rightarrow \mathcal{A}\mathcal{C}_T$ as

$$\begin{aligned} \mathcal {S}_V(r,\eta )(t)(x)&:= r_0(x)\\&\quad - \int _0^t \int _{\mathbb {R}^d\backslash \{x\}} \Phi (r_s(x), r_s(y);V_s[r](x,y))\eta _s(x,y)\textrm{d}\mu (y)\textrm{d}s, \quad \mu \text {-a.e.}\ x \in \mathbb {R}^d, \\ \mathcal {S}_\omega (r,\eta )(t) (x,y)&:= \eta _0(x,y) + \int _0^t \left( \omega _s[r](x,y) - \eta _s(x,y)\right) \textrm{d}s\ , \end{aligned}$$

for any $t \in [0,T]$ and $(x,y) \in {\mathbb {R}^{2d}_{\!\scriptscriptstyle \diagup }}$. The a priori properties in Proposition 3.1 ensure this map $\mathcal {S}$ is well defined. Below, we prove existence and uniqueness of solutions to (Euler Co-NCL) by assuming a uniform-in-time integrability assumption for the velocity field *V* motivated by our guiding example, see (3.4), although the result holds for *V* satisfying (3.5). The stronger assumption is relevant in Sect. 4, where it is used to obtain the Lipschitzness of the characteristics.

### Theorem 3.1

Let $\Phi$ be a jointly antisymmetric admissible flux interpolation as in Definition 2.2 and consider an initial datum $(r^0, \eta ^0) \in L^2_\mu (\mathbb {R}^d)\times C_b({\mathbb {R}^{2d}_{\!\scriptscriptstyle \diagup }})$, with $\eta ^0$ symmetric. Assume that $V:[0,T]\times L^2_\mu (\mathbb {R}^d)\rightarrow \mathcal {V}^{as}({\mathbb {R}^{2d}_{\!\scriptscriptstyle \diagup }})$ satisfies (3.5) or

3.9a $$\begin{aligned} \sup _{t\in [0,T]} \sup _{r \in L^2_\mu (\mathbb {R}^d)} \sup _{x\in \mathbb {R}^d} \int _{\mathbb {R}^d\backslash \{x\}}|V_t[r](x,x')|^2\textrm{d}\mu (x') \le C_{V} , \end{aligned}$$

and, for all $t\in [0,T]$ and any $g,\tilde{g}\in L^2_\mu (\mathbb {R}^d)$,

3.9b $$\begin{aligned} \sup _{x\in \mathbb {R}^d} \int _{\mathbb {R}^d\backslash \{x\}}|V_t[g](x,x') - V_t[\tilde{g}](x,x')|^2\textrm{d}\mu (x') \le L_V\Vert g-\tilde{g} \Vert ^2_{L^2_\mu (\mathbb {R}^d)}, \end{aligned}$$

for constants $$C_V, L_V>0$$.

Assume that $\omega :[0,T]\times L^2_\mu (\mathbb {R}^d) \times {\mathbb {R}^{2d}_{\!\scriptscriptstyle \diagup }}\rightarrow \mathbb {R}$ satisfies $({\boldsymbol{\omega }}1)$–$({\boldsymbol{\omega }}4)$. Then, there exists a solution $(r, \eta )$ to (Euler Co-NCL) with initial datum $(r^0,\eta ^0)$, as in Definition 3.1.

### Proof

Let us assume (3.9a) since the argument is slightly more involved for this case at the end of the proof. For $(r^0,\eta ^0)$ given, we consider $(r,\eta ), (\tilde{r},\tilde{\eta }) \in \mathcal{A}\mathcal{C}_T$ and, for $\mu$-a.e. $x \in \mathbb {R}^d$, we have

$$\begin{aligned}&|\mathcal {S}_V(r,\eta )(t) - \mathcal {S}_V(\tilde{r},\tilde{\eta })(t)|(x) \\&\quad \le \int _0^t \int _{\mathbb {R}^d\backslash \{x\}}\Big | \Phi (r_s(x), r_s(y);V_s[r](x,y))\eta _s(x,y)\\&\qquad \qquad - \Phi (\tilde{r}_s(x), \tilde{r}_s(y);V_s[\tilde{r}](x,y))\tilde{\eta }_s(x,y)\Big |\textrm{d}\mu (y)\textrm{d}s \\&\quad \le L_\Phi \int _0^t \int _{\mathbb {R}^d\backslash \{x\}}\big |\eta _s(x,y)\big | (|r_s(x) - \tilde{r}_s(x)| + |r_s(y) - \tilde{r}_s(y)|)|V_s[r](x,y)|\textrm{d}\mu (y)\textrm{d}s \\&\qquad +L_\Phi \int _0^t \int _{\mathbb {R}^d\backslash \{x\}}\left( |\tilde{r}_s(x)| + |\tilde{r}_s(y)|\right) |V_s[r](x,y)|\big |\eta _s(x,y) - \tilde{\eta }_s(x,y)\big | \textrm{d}\mu (y)\textrm{d}s \\&\qquad + L_\Phi \int _0^t \int _{\mathbb {R}^d\backslash \{x\}}\big |\tilde{\eta }_s(x,y)\big |\left( |\tilde{r}_s(x)| + |\tilde{r}_s(y)|\right) |V_s[r](x,y) - V_s[\tilde{r}](x,y)|\textrm{d}\mu (y)\textrm{d}s \\&\quad =: I + II + III, \end{aligned}$$

where we used (i), (ii) in Definition 2.2. *I* can be estimated as follows by means of Cauchy–Schwarz and Minkowski inequalities:

3.10 $$\begin{aligned} |I|&\le L_\Phi \Vert \eta \Vert _{\infty , C_b({\mathbb {R}^{2d}_{\!\scriptscriptstyle \diagup }})} \left( \sup _{t \in [0,T]}\sup _{r \in L^2_\mu (\mathbb {R}^d)}\sup _{x\in \mathbb {R}^d}\int _{\mathbb {R}^d\backslash \{x\}}\left| V_t[r](x,y)\right| ^2\textrm{d}\mu (y)\right) ^{1/2} \nonumber \\ &\qquad \qquad \qquad \times \int _{0}^t\left( \int _{\mathbb {R}^d\backslash \{x\}}\left( |r_s(x)-\tilde{r}_s(x)| + |r_s(y) - \tilde{r}_s(y)|\right) ^2\textrm{d}\mu (y)\right) ^{1/2}\!\!\! \textrm{d}s \\&\le L_\Phi \Vert \eta \Vert _{\infty , C_b({\mathbb {R}^{2d}_{\!\scriptscriptstyle \diagup }})}C_V^{1/2}\int _{0}^t(\Vert r_s-\tilde{r}_s \Vert _{L^2_\mu (\mathbb {R}^d)} + |r_s(x) - \tilde{r}_s(x)|)\textrm{d}s\ , \nonumber \end{aligned}$$

where in the second inequality we used (3.9a). Squaring both sides and integrating with respect to $\mu$

$$\begin{aligned} \Vert I \Vert ^2_{L^2_\mu (\mathbb {R}^d)}&\le L_\Phi ^2 \Vert \eta \Vert _{\infty ,C_b({\mathbb {R}^{2d}_{\!\scriptscriptstyle \diagup }})}^2 C_V \int _{\mathbb {R}^d}\bigg |\int _{0}^t(\Vert r_s-\tilde{r}_s \Vert _{L^2_\mu (\mathbb {R}^d)} + |r_s(x) - \tilde{r}_s(x)|)\textrm{d}s\bigg |^2 \textrm{d}\mu (x) \\&\le L_\Phi ^2 \Vert \eta \Vert _{\infty ,C_b({\mathbb {R}^{2d}_{\!\scriptscriptstyle \diagup }})}^2C_V T\int _{\mathbb {R}^d}\int _0^T(\Vert r_s-\tilde{r}_s \Vert _{L^2_\mu (\mathbb {R}^d)} + |r_s(x) - \tilde{r}_s(x)|)^2\textrm{d}s\textrm{d}\mu (x) \\&\le L_\Phi ^2 \Vert \eta \Vert _{\infty ,C_b({\mathbb {R}^{2d}_{\!\scriptscriptstyle \diagup }})}^2 2C_V T\int _0^T \Vert r_s-\tilde{r}_s \Vert ^2_{L^2_\mu (\mathbb {R}^d)} \textrm{d}s\ \\&\le L_\Phi ^2 \Vert \eta \Vert _{\infty ,C_b({\mathbb {R}^{2d}_{\!\scriptscriptstyle \diagup }})}^2 2C_V T^2\Vert r-\tilde{r} \Vert ^2_{\infty , L^2_\mu (\mathbb {R}^d)}, \end{aligned}$$

thus

$$ \Vert I \Vert _{L^2_\mu (\mathbb {R}^d)} \le \sqrt{2} L_\Phi \Vert \eta \Vert _{\infty , C_b({\mathbb {R}^{2d}_{\!\scriptscriptstyle \diagup }})} C_V^{1/2}\Vert r - \tilde{r} \Vert _{\infty , L^2_\mu (\mathbb {R}^d)}{T}. $$

For *II*, we obtain similarly that

$$ |II| \le L_\Phi \Vert \eta -\tilde{\eta } \Vert _{\infty , C_b({\mathbb {R}^{2d}_{\!\scriptscriptstyle \diagup }})}C_V^{1/2}\int _0^t( \Vert \tilde{r}_s \Vert _{L^2_\mu (\mathbb {R}^d)}+ |r_s(x)|)\textrm{d}s, $$

and thus

$$\begin{aligned} \Vert II \Vert _{L^2_\mu (\mathbb {R}^d)}&\le L_\Phi \Vert \eta -\tilde{\eta } \Vert _{\infty , C_b({\mathbb {R}^{2d}_{\!\scriptscriptstyle \diagup }})}C_V^{1/2}(\Vert r \Vert _{\infty ,L^2_\mu (\mathbb {R}^d)} + \Vert \tilde{r} \Vert _{\infty ,L^2_\mu (\mathbb {R}^d)}) T\ \\&\le 2 L_\Phi \Vert \eta -\tilde{\eta } \Vert _{\infty , C_b({\mathbb {R}^{2d}_{\!\scriptscriptstyle \diagup }})}C_V^{1/2}\bar{C}_T T \ , \end{aligned}$$

where in the last inequality $\bar{C}_T=\Vert r_0 \Vert _{L^2_\mu (\mathbb {R}^d)}e^{2L_\Phi \Vert \eta \Vert _\infty C_V T}$, from (3.8). Finally, for *III* we use (3.9b) to have

$$\begin{aligned} |III|&\le L_\Phi \Vert \tilde{\eta } \Vert _{\infty , C_b({\mathbb {R}^{2d}_{\!\scriptscriptstyle \diagup }})}\left( \sup _{t\in [0,T]}\sup _{r,\tilde{r}\in L^2_\mu (\mathbb {R}^d)}\sup _{x \in \mathbb {R}^d} \int _{\mathbb {R}^d\backslash \{x\}}|V_t[r](x,y) - V_t[\tilde{r}](x,y)|^2 \textrm{d}\mu (y)\right) ^{1/2} \\&\qquad \qquad \qquad \qquad \qquad \times \int _0^t \Vert \tilde{r}_s \Vert _{L^2_\mu (\mathbb {R}^d)} + |\tilde{r}_s(x)|\textrm{d}s\ \\&\le L_\Phi \Vert \tilde{\eta } \Vert _{\infty , C_b({\mathbb {R}^{2d}_{\!\scriptscriptstyle \diagup }})} L_V^{1/2} \Vert r - \tilde{r} \Vert _{\infty , L^2_\mu (\mathbb {R}^d)}\int _0^t \Vert \tilde{r}_s \Vert _{L^2_\mu (\mathbb {R}^d)} + |\tilde{r}_s(x)|\textrm{d}s. \end{aligned}$$

Therefore,

$$\begin{aligned} \Vert III \Vert _{L^2_\mu (\mathbb {R}^d)}&\le \sqrt{2} L_\Phi \Vert \tilde{\eta } \Vert _{\infty , C_b({\mathbb {R}^{2d}_{\!\scriptscriptstyle \diagup }})} L_V^{1/2}\Vert r \Vert _{ \infty , L^2_\mu (\mathbb {R}^d)}\Vert r - \tilde{r} \Vert _{\infty , L^2_\mu (\mathbb {R}^d)}T\ \\&\le \sqrt{2} L_\Phi \Vert \tilde{\eta } \Vert _{\infty , C_b({\mathbb {R}^{2d}_{\!\scriptscriptstyle \diagup }})} L_V^{1/2}\Vert r - \tilde{r} \Vert _{\infty , L^2_\mu (\mathbb {R}^d)}\bar{C}_TT. \end{aligned}$$

The same estimate for $\mathcal {S}_\omega$ gives

$$\begin{aligned} \Vert \mathcal {S}_\omega (r,\eta ) - \mathcal {S}_\omega (\tilde{r},\tilde{\eta }) \Vert _{\infty , C_b({\mathbb {R}^{2d}_{\!\scriptscriptstyle \diagup }})}&\le \Vert \eta -\tilde{\eta } \Vert _{\infty , C_b({\mathbb {R}^{2d}_{\!\scriptscriptstyle \diagup }})} T + L_\omega \Vert r - \tilde{r} \Vert _{\infty , L^2_\mu (\mathbb {R}^d)}T, \end{aligned}$$

due to $({\boldsymbol{\omega }}2)$ and Proposition 3.1, see also (Esposito and Mikolás 2024, Lemma 3.3). A combination of the previous estimates implies that

$$\begin{aligned} d_\infty ((r,\eta ),(\tilde{r},\tilde{\eta }))&\le (\sqrt{2} L_\Phi \Vert \tilde{\eta } \Vert _{\infty , C_b({\mathbb {R}^{2d}_{\!\scriptscriptstyle \diagup }})}( L_V^{1/2}\bar{C}_T+C_V^{1/2}) + L_\omega ))\Vert r - \tilde{r} \Vert _{\infty , L^2_\mu (\mathbb {R}^d)} T \\&\quad + ( 1 + 2L_\Phi C_V^{1/2}\bar{C}_T)\Vert \eta - \tilde{\eta } \Vert _{\infty , C_b({\mathbb {R}^{2d}_{\!\scriptscriptstyle \diagup }})}T\\&\le \underbrace{(\sqrt{2} L_\Phi (\Vert \eta _0 \Vert _{\infty }+C_\omega T)e^T( L_V^{1/2}\bar{C}_T+C_V^{1/2}) + L_\omega ))}_{:=\alpha (T)}\Vert r - \tilde{r} \Vert _{\infty , L^2_\mu (\mathbb {R}^d)} T \\&\quad + \underbrace{( 1 + 2L_\Phi C_V^{1/2}\bar{C}_T)}_{:=\beta (T)}\Vert \eta - \tilde{\eta } \Vert _{\infty , C_b({\mathbb {R}^{2d}_{\!\scriptscriptstyle \diagup }})}T \end{aligned}$$

where we used (3.6) with $({\boldsymbol{\omega }}3)$. In particular, it follows that

$$ d_\infty (\mathcal {S}(r,\eta ),\mathcal {S}(\tilde{r},\tilde{\eta })) \le \max \{\alpha (T),\beta (T)\}Td_\infty ((r,\eta ),(\tilde{r},\tilde{\eta })) $$

so that for $T < \kappa (T):=1/\max \{\alpha (T), \beta (T)\}$ the solution map is a contraction. By the Banach fixed-point theorem, we can conclude there exists a unique solution to (Euler Co-NCL) in $C_T=C([0,T]; L^2_\mu (\mathbb {R}^d)) \times C([0,T]; C_b({\mathbb {R}^{2d}_{\!\scriptscriptstyle \diagup }}))$ for $T<T^*$, where $T^*$ is such that $\kappa (T^*)^{-1}T^*=1$. This can be verified since $\alpha (T)\simeq 1+ e^T + Te^T$ and $\beta (T) \simeq 1 + e^{T^2e^T}$. Absolute continuity in time follows since $\overline{\nabla }\cdot F[\mu , \eta ; r, V[r]]$ and the map $(t\mapsto \omega _t[r_t]-\eta _t)$ belong to $L^1([0,T])$, as proven in Proposition 3.1. The solution can be extended by using a standard iteration procedure so that the time interval does not depend on any $T^*$, see, e.g. (Esposito and Mikolás 2024, Theorem 3.6).

Now we turn to the analysis of the case *V* satisfying (3.5). Following the same strategy, upon using (3.5) instead of (3.9a), we obtain the bound

$$\begin{aligned} d_\infty ((r,\eta ),(\tilde{r},\tilde{\eta }))&\le \underbrace{(\sqrt{2} L_\Phi (\Vert \eta _0 \Vert _{\infty }+C_\omega T)e^T( L_V^{1/2}\bar{C}+C_V^{1/2}) + L_\omega ))}_{:=\alpha (T)}\Vert r - \tilde{r} \Vert _{\infty , L^2_\mu (\mathbb {R}^d)} (T+\sqrt{T}) \\&\quad + \underbrace{( 1 + 2L_\Phi C_V^{1/2}\bar{C})}_{:=\beta }\Vert \eta - \tilde{\eta } \Vert _{\infty , C_b({\mathbb {R}^{2d}_{\!\scriptscriptstyle \diagup }})}(T+\sqrt{T})\ , \end{aligned}$$

where $\bar{C}=\Vert r_0 \Vert _{L^2_\mu (\mathbb {R}^d)}e^{2L_\Phi \Vert \eta \Vert _\infty C_V}$ and thus

$$ d_\infty (\mathcal {S}(r,\eta ),\mathcal {S}(\tilde{r},\tilde{\eta })) \le \max \{\alpha (T),\beta \}(T+\sqrt{T})d_\infty ((r,\eta ),(\tilde{r},\tilde{\eta })),\, $$

so that for $T+\sqrt{T} < \kappa (T):=1/\max \{\alpha (T), \beta \}$ the solution map is a contraction. The rest of the argument follows the previous case in a completely analogous way. $\square$

### Remark 3.1

The assumptions on the joint antisymmetry of $\Phi$ and the symmetry of $\eta$ through $({\boldsymbol{\omega }}4)$ and $\eta _0$ symmetric in Theorem 3.1 are needed so that (Co-NCL) can be expressed as (Euler Co-NCL), see Esposito et al. (2023), Section 5. However, these assumptions are not required to obtain well-posedness of the dynamics described by (Euler Co-NCL) itself.

Since we proved existence and uniqueness of solutions to (Euler Co-NCL), we note that equation (3.2b) admits the explicit solution depending on *r*:

3.11 $$\begin{aligned} \eta _t[r](x, y)=e^{-t }\left( \eta _0(x, y)+\int _0^t e^{s} \omega _s\left[ r\right] (x, y) \textrm{d} s\right) \ . \end{aligned}$$

Thus, the bound on $\Vert \eta \Vert _{\infty ,C_b({\mathbb {R}^{2d}_{\!\scriptscriptstyle \diagup }})}$ can be improved to

3.12 $$\begin{aligned} \Vert \eta \Vert _{\infty ,C_b({\mathbb {R}^{2d}_{\!\scriptscriptstyle \diagup }})} \le \Vert \eta _0 \Vert _{C_b({\mathbb {R}^{2d}_{\!\scriptscriptstyle \diagup }})} + C_\omega \ . \end{aligned}$$

Having proved the well-posedness of (Euler Co-NCL), we turn to the study of the graph-continuity equation in the next section.

## The Graph-Continuity Equation

Throughout this section, we consider a co-evolving graph, $\mathcal {G}_t=(\mu ,\eta _t[r])$, for $(r,\eta )$ a solution to (Euler Co-NCL) in the sense of Definition 3.1, which exists and is unique due to Theorem 3.1. We observe that (Euler Co-NCL) is linked to a particular nonlinear continuity equation if we consider the evolution of the measure $\delta _{r_t}\otimes \mu$, see also (Paul and Trélat 2024, Section 3.1.3) for a similar case. More precisely, for any Borel set $A\times B \in \mathcal {B}(\mathbb {R}\times \mathbb {R}^d)$ let us consider the measure $\sigma := \delta _r \otimes \mu$ given by

4.1 $$\begin{aligned} ( \delta _r\otimes \mu )(A \times B) = \int _{B}\delta _{r(x)}(A) \textrm{d}\mu (x), \end{aligned}$$

where $(r, \eta )$ is the solution of (Euler Co-NCL) with base measure $\mu \in \mathcal {P}_2(\mathbb {R}^d)$. One can check that $\delta _{r_t}\otimes \mu$ is a weak solution (in the sense of Definition 4.1) of the following initial value problem:

graph-CE $$\begin{aligned} {\left\{ \begin{array}{ll} \partial _t \sigma + \partial _{\xi }(\sigma \mathcal {X}[\sigma , r, \eta ]) = 0,\\ \sigma _0 = \bar{\sigma } \in \mathcal {P}(\mathbb {R}\times \mathbb {R}^d), \end{array}\right. } \end{aligned}$$

where $\xi \in \mathbb {R}$ and, for any $\sigma \in \mathcal {P}(\mathbb {R}\times \mathbb {R}^d)$, the velocity field is

4.2 $$\begin{aligned} \mathcal {X}[\sigma ,r, \eta ](t,\xi ,x):=-\int _{\mathbb {R}\times \mathbb {R}^d\backslash \{x\}}\Phi ( \xi , \xi '; V_t[r](x,x'))\eta _t(x,x')\textrm{d}\sigma (\xi ',x') \ , \end{aligned}$$

for a solution $(r,\eta )$ to $({Euler Co-NCL})$. We refer to (graph-CE) as the *graph-continuity equation* since the velocity field depends on the solution of the co-evolving graph problem. The measure in (4.1) can be interpreted as the amount of mass in $A\subseteq \mathbb {R}$ over the vertices located in $B\subseteq \mathbb {R}^d$. If $\mu$ is the Lebesgue measure on $\mathbb {R}^d$, $\sigma$ is supported on the graph of the map $x\mapsto r(x)$. Since the base measure, $\mu$, is constant in time, (graph-CE) describes the evolution of the density of mass over the vertices of the graph, which are fixed entities. (graph-CE) is actually a structured dynamics where the vertices are parameters. We embed (Euler Co-NCL) in (graph-CE) to obtain new insights in the study of the long-time behaviour of its solutions as consequence of contractivity. For ease of presentation, we split this section into two parts. First we prove that (graph-CE) is well-posed, then we move to the contraction property with respect to a suitable distance.

### Well-Posedness for (graph-CE)

The solutions to (graph-CE) are understood in the distributional sense as follows.

#### Definition 4.1

Let $\mathcal {G}_t=(\mu ,\eta _t[r])$ be a given co-evolving graph, where $(r,\eta )$ solve (Euler Co-NCL). A curve $\sigma \in C([0,T], \mathcal {P}(\mathbb {R}\times \mathbb {R}^d))$ is a solution to (graph-CE) with initial data $\sigma _0 \in \mathcal {P}(\mathbb {R}\times \mathbb {R}^d)$ if, for any $\varphi \in C_c^1([0,T] \times \mathbb {R}\times \mathbb {R}^d)$, it holds

4.3 $$\begin{aligned} \begin{aligned}&\int _0^T\int _{\mathbb {R}\times \mathbb {R}^d} \partial _t\varphi (t, \xi , x) d \sigma _t(\xi , x)\textrm{d}t\\&\qquad + \int _{\mathbb {R}\times \mathbb {R}^d} \varphi (0,\xi ,x) \textrm{d}\sigma _0(\xi ,x) - \int _{\mathbb {R}\times \mathbb {R}^d} \varphi (T,\xi ,x) \textrm{d}\sigma _{T}(\xi ,x) \\&\quad =\int _0^T \int _{\mathbb {R}\times \mathbb {R}^d} \int _{\mathbb {R}\times \mathbb {R}^d\backslash \{x\}}\partial _{\xi } \varphi (t,\xi , x)\Phi ( \xi , \xi '; V_t[r](x,x'))\eta _t(x,x') \textrm{d}\sigma _t(\xi ^{\prime }, x^{\prime }) \textrm{d}\sigma _t(\xi , x) \textrm{d}t \ . \end{aligned} \end{aligned}$$

We are interested on evolutions on graphs, whose possible vertices are given by $\mu$, which is constant in time. For this reason, it makes sense to consider the disintegration of a solution $\sigma _{t}$ to (graph-CE) of the form (4.1) with respect to the marginal $\mu$ on $\mathbb {R}^d$, i.e. $\sigma _{t} = \int _{\mathbb {R}^d} \sigma _{t,x}\textrm{d}\mu (x)$. In what follows, we will denote by $\pi ^1:\mathbb {R}\times \mathbb {R}^d\rightarrow \mathbb {R}$ and $\pi ^2:\mathbb {R}\times \mathbb {R}^d\rightarrow \mathbb {R}^d$ the canonical projections given by $\pi ^1(x,x') = x$ and $\pi ^2(x,x') = x'$ for any $(x,x') \in \mathbb {R}\times \mathbb {R}^d$, so that $\mu = \pi ^2_\# \sigma$ for any measure $\sigma$ on $\mathbb {R}\times \mathbb {R}^d$ with marginal $\mu$ on $\mathbb {R}^d$. By uniqueness $\mu$-almost everywhere of the disintegration, we consider the *disintegrated version* of (graph-CE)

4.4 $$\begin{aligned} {\left\{ \begin{array}{ll} \partial _t \sigma _{t,x} + \partial _\xi (\sigma _{t,x}\mathcal {X}[\sigma ,r,\eta ](t,\cdot , x)) = 0,\\ \sigma _{0,x}=\bar{\sigma }_x, \end{array}\right. } \end{aligned}$$

for $\mu$-almost every $x\in \mathbb {R}^d$ and $\bar{\sigma }=\int _{\mathbb {R}^d}\bar{\sigma }_x\textrm{d}\mu (x)$. For $\mu$-a.e. $x\in \mathbb {R}^d$, we interpret the above equation in the distributional sense, i.e. for any $\varphi \in C^1_c([0,T]\times \mathbb {R}\times \mathbb {R}^d)$,

4.5 $$\begin{aligned} \begin{aligned}&\int _0^T\int _{\mathbb {R}} \partial _t\varphi (t, \xi , x) d \sigma _{t,x}(\xi )\textrm{d}t + \int _{\mathbb {R}} \varphi (0,\xi ,x) \textrm{d}\sigma _{0,x}(\xi ) - \int _{\mathbb {R}} \varphi (T,\xi ,x) \textrm{d}\sigma _{T,x}(\xi ) \\&\quad =\int _0^T \int _{\mathbb {R}} \int _{\mathbb {R}\times \mathbb {R}^d\backslash \{x\}}\partial _{\xi } \varphi (t,\xi , x)\Phi ( \xi , \xi '; V_t[r](x,x'))\eta _t(x,x') \textrm{d}\sigma _t(\xi ^{\prime }, x^{\prime }) \textrm{d}\sigma _{t,x}(\xi ) \textrm{d}t . \end{aligned} \end{aligned}$$

Note that integrating (4.5) with respect to $\mu$ we obtain $\sigma _t=\int _{\mathbb {R}^d}\sigma _{t,x}\textrm{d}\mu (x)$ is a distributional solution to (graph-CE). Due to the nature of our problem, we introduce the notation

$$ \mathcal {P}^\mu _2(\mathbb {R}\times \mathbb {R}^d):=\{\nu \in \mathcal {P}_2(\mathbb {R}\times \mathbb {R}^d) \ | \ {\pi _2}_\# \nu = \mu \}\, $$

for measures in $\mathcal {P}_2(\mathbb {R}\times \mathbb {R}^d)$ having the marginal $\mu$ on $\mathbb {R}^d$. This means we will consider the embedding of the dynamics (Euler Co-NCL) into (graph-CE) for graphs with the same base measure $\mu \in \mathcal {P}_2(\mathbb {R}^d)$. We equip $\mathcal {P}_2^\mu (\mathbb {R}\times \mathbb {R}^d)$ with the distance

$$ L^2_\mu d_2(\nu ^1,\nu ^2):=\left( \int _{\mathbb {R}^d}d^2_2(\nu ^1_x,\nu ^2_x) \textrm{d}\mu (x)\right) ^{\frac{1}{2}}, \quad \nu ^1,\nu ^2 \in \mathcal {P}^\mu _2(\mathbb {R}\times \mathbb {R}^d)\, $$

which forms a complete metric space, see Mikolas (2024), Proposition A.0.1 for the formal statement and proof. For $\sigma \in \mathcal {P}_2^\mu (\mathbb {R}\times \mathbb {R}^d)$, we denote by $m_i[\sigma _{t,x}]:=\int _{\mathbb {R}}|\xi |^i\textrm{d}\sigma _{t,x}(\xi )$ $\mu$-a.e. $x \in \mathbb {R}^d$, and $t \in [0,T]$ the *i*-th moment of the disintegration of $\sigma$ with respect to $\mu$, for $i=1,2$, and we will denote by $m_i[\gamma _*]:= \sup _{t \in [0,T]}m_i[\gamma _t]$ the bound in time for the *i*-th moment of a curve $\gamma \in C([0,T];\mathcal {P}(\mathbb {R}))$. In order to prove existence and uniqueness of weak solutions, we proceed by looking at the characteristics’ equation, as usual for continuity-type equation.

#### Lemma 4.1

Let $V:[0,T]\times L^2_\mu (\mathbb {R}^d) \rightarrow \mathcal {V}^{as}({\mathbb {R}^{2d}_{\!\scriptscriptstyle \diagup }})$ be continuous in time and assume it satisfies (3.9a) and (3.9b). Let $\sigma \in C([0,T], \mathcal {P}^{\mu }_2(\mathbb {R}{\times \mathbb {R}^d}))$ with ${\pi _1}_\#\sigma =\gamma \in C([0,T];\mathcal {P}(\mathbb {R}))$ and $(r,\eta )$ be the solution of (Euler Co-NCL). Then, for $\mu$-a.e. $x \in \mathbb {R}^d$, the ODE initial value problem

$$\begin{aligned} \frac{\hbox {d}}{\hbox {d}t} v_t(x)&= \mathcal {X}[\sigma ,r,\eta ](t,v_t(x), x) \ , \\ v_0(x)&= u \in \mathbb {R}\ , \end{aligned}$$

has a unique solution on [0, *T*].

#### Proof

We begin by showing that our assumptions imply the map $\mathbb {R}\ni \xi \mapsto \mathcal {X}[\sigma ,r,\eta ](t,\xi ,x)$ is globally Lipschitz, for any $(t,x)\in [0,T]\times \mathbb {R}^d$. Indeed, for any $a,b \in \mathbb {R}$ we use (i), (ii) from Definition 2.2, and (3.9a) to obtain

4.6 $$\begin{aligned} {\begin{matrix} & |\mathcal {X}[\sigma ,r,\eta ](t,a,x) - \mathcal {X}[\sigma ,r,\eta ](t,b,x)|\\ & \quad \le \ \int _{\mathbb {R}\times \mathbb {R}^d\backslash \{x\}} \bigg |(\Phi (a,\xi ';V_t[r](x,x'))- \Phi (b,\xi ';V_t[r](x,x'))\eta _t(x,x')\bigg |\textrm{d}\sigma _t(\xi ',x') \\ & \quad \le L_\Phi \int _{\mathbb {R}\times \mathbb {R}^d\backslash \{x\}} |\eta _t(x,x')|\ |V_t[r](x,x')|\ |a-b| \textrm{d}\sigma _{t,x'}(\xi ')\textrm{d}\mu (x') \\ & \quad \le L_\Phi C_V^{1/2} \Vert \eta \Vert _{\infty , C_b({\mathbb {R}^{2d}_{\!\scriptscriptstyle \diagup }})} |a - b|. \end{matrix}} \end{aligned}$$

Let us check that $t \mapsto \mathcal {X}[\sigma ,r,\eta ](t,a,x)$ is continuous. Without loss of generality, for any $(a,x) \in \mathbb {R}\times \mathbb {R}^d$ and $s<t \in [0,T]$, consider

$$\begin{aligned}&|\mathcal {X}[\sigma ,r,\eta ](t,a,x) - \mathcal {X}[\sigma ,r,\eta ](s,a,x)| \\&\quad = \bigg |\int _{\mathbb {R}\times \mathbb {R}^d\backslash \{x\}}\!\!\Phi (a,\xi ';V_t[r](x,x')\eta _t(x,x')\textrm{d}\sigma _t(\xi ',x')\\&\qquad \quad - \int _{\mathbb {R}\times \mathbb {R}^d\backslash \{x\}}\!\!\Phi (a,\xi ';V_s[r](x,x'))\eta _s(x,x') \textrm{d}\sigma _s(\xi ',x') \nonumber \bigg | \\&\le \bigg | \int _{\mathbb {R}\times \mathbb {R}^d\backslash \{x\}} \Phi (a,\xi ';V_t[r](x,x')\eta _t(x,x')\textrm{d}(\sigma _t-\sigma _s)(\xi ',x') \bigg | \\&\qquad + \int _{\mathbb {R}\times \mathbb {R}^d\backslash \{x\}}\bigg | \Phi (a,\xi ';V_t[r](x,x'))\eta _t(x,x') - \Phi (a,\xi ';V_s[r](x,x'))\eta _s(x,x')\bigg | \textrm{d}\sigma _s(\xi ',x') \\&\quad =: I + II\ . \end{aligned}$$

We begin by estimating *I*. Note that for $(\mu \otimes \mu )$-a.e. $(x,x') \in {\mathbb {R}^{2d}_{\!\scriptscriptstyle \diagup }}$, $a\in \mathbb {R}$, and any $t \in [0,T]$ we have

4.7 $$\begin{aligned} {\begin{matrix} Lip[\Phi & (a,\cdot ,V_t[r](x,x'))\eta _t(x, x')] \\ & :=\sup _{\xi ^1\ne \xi ^2\in \mathbb {R}}\frac{|(\Phi (a,\xi ^1;V_t[r](x,x')-\Phi (a,\xi ^2;V_t[r](x,x'))\eta _t|}{|\xi ^1-\xi ^2|} \\ & \le L_\Phi |\eta _t(x,x')||V_t[r](x,x')| \\ & \le L_\Phi \Vert \eta \Vert _{\infty , C_b({\mathbb {R}^{2d}_{\!\scriptscriptstyle \diagup }})}|V_t[r](x,x')| \ , \end{matrix}} \end{aligned}$$

similarly to (4.6). Thus, the Lipschitz constant of the map $\xi \mapsto \Phi (a,\xi ;V_t[r](x,x'))\eta _t(x,x')$ is bounded by $L_\Phi \Vert \eta \Vert _{\infty , C_b({\mathbb {R}^{2d}_{\!\scriptscriptstyle \diagup }})}|V_t[r](x,x')|$ for $(\mu \otimes \mu )$-a.e. $(x,x') \in \mathbb {R}^d\times \mathbb {R}^d$ and any $t \in [0,T]$. Then, we can estimate *I* by

$$\begin{aligned} |I|&= \bigg | \int _{ \mathbb {R}^d\backslash \{x\}}\int _{\mathbb {R}} (\Phi (a,\xi ';V_t[r](x,x'))\eta _t(x,x')\textrm{d}(\sigma _{t,x}-\sigma _{s,x})(\xi ')\textrm{d}\mu (x') \bigg | \\&\le \int _{\mathbb {R}^d\backslash \{x\}} \bigg |\int _{\mathbb {R}} \Phi (a,\xi ';V_t[r](x,x'))\eta _t(x,x')\textrm{d}(\sigma _{t,x'}-\sigma _{s,x'})(\xi ')\bigg | \textrm{d}\mu (x') \\&\le \int _{\mathbb {R}^d} Lip[\Phi (a,\cdot ,V_t[r](x,x')\eta _t(x,x')]d_1(\sigma _{t,x'},\sigma _{s,x'})\textrm{d}\mu (x') \\&\le L_\Phi \Vert \eta \Vert _{\infty ,C_b({\mathbb {R}^{2d}_{\!\scriptscriptstyle \diagup }})} \int _{\mathbb {R}^d}|V_t[r](x,x')|d_1(\sigma _{t,x'},\sigma _{s,x'})\textrm{d}\mu (x') \\&\le L_\Phi \Vert \eta \Vert _{\infty ,C_b({\mathbb {R}^{2d}_{\!\scriptscriptstyle \diagup }})} C_V^{1/2}L^2_\mu d_2(\sigma _t,\sigma _s), \end{aligned}$$

where we used Cauchy–Schwarz inequality and (3.9a). By the continuity in time of $\sigma$, we have $I\rightarrow 0$ as $t\rightarrow s$.

Let us now focus on *II*. For any $a \in \mathbb {R}$ and $x \in \mathbb {R}^d$, note that $\Phi _t-\Phi _s:=\Phi (a,\xi ';V_t[r](x,x'))\eta _t(x,x') - \Phi (a,\xi ';V_s[r](x,x'))\eta _s(x,x')$ is integrable, since

$$\begin{aligned} |II|&\le \int _{\mathbb {R}\times \mathbb {R}^d\backslash \{x\}}\bigg |\Phi (a,\xi ';V_s[r](x,x'))\eta _s(x,x')\bigg |\textrm{d}\sigma _{s}(\xi ',x') \\&\quad +\int _{\mathbb {R}\times \mathbb {R}^d\backslash \{x\}}\bigg |\Phi (a,\xi ';V_t[r](x,x'))\eta _t(x,x')\bigg |\textrm{d}\sigma _{s}(\xi ',x') \\&\le L_\Phi \Vert \eta \Vert _{\infty ,C_b({\mathbb {R}^{2d}_{\!\scriptscriptstyle \diagup }})}\bigg (|a|\int _{\mathbb {R}^d}|V_s[r](x,x')|\textrm{d}\mu (x')\\&\qquad + \int _{\mathbb {R}^d}|V_s[r](x,x')|\int _{\mathbb {R}}|\xi '|\textrm{d}\sigma _{s,x'}(\xi ')\textrm{d}\mu (x') \\&\qquad + |a|\int _{\mathbb {R}^d}|V_t[r](x,x')|\textrm{d}\mu (x') + \int _{\mathbb {R}^d}|V_t[r](x,x')|\int _{\mathbb {R}}|\xi '|\textrm{d}\sigma _{s,x'}(\xi ')\textrm{d}\mu (x')\bigg ) \\&=L_\Phi \Vert \eta \Vert _{\infty ,C_b({\mathbb {R}^{2d}_{\!\scriptscriptstyle \diagup }})}\bigg (|a|\int _{\mathbb {R}^d}|V_s[r](x,x')\textrm{d}\mu (x') + \int _{\mathbb {R}^d}|V_s[r](x,x')|m_1[\sigma _{s,x'}]\textrm{d}\mu (x') \\&\qquad + |a|\int _{\mathbb {R}^d}|V_t[r](x,x')|\textrm{d}\mu (x') + \int _{\mathbb {R}^d}|V_t[r](x,x')|m_1[\sigma _{s,x'}]\textrm{d}\mu (x')\bigg )\\&\le 2L_\Phi \Vert \eta \Vert _{\infty ,C_b({\mathbb {R}^{2d}_{\!\scriptscriptstyle \diagup }})} C_V^{1/2} |a| + L_\Phi \Vert \eta \Vert _{\infty ,C_b({\mathbb {R}^{2d}_{\!\scriptscriptstyle \diagup }})}C_V^{1/2}\bigg (\left( \int _{\mathbb {R}^d}m_1^2[\sigma _{s,x'}]\textrm{d}\mu (x')\right) ^{1/2} \\&\qquad + \left( \int _{\mathbb {R}^d}m_1^2[\sigma _{s,x'}]\textrm{d}\mu (x')\right) ^{1/2}\bigg )\\&= 2L_\Phi \Vert \eta \Vert _{\infty ,C_b({\mathbb {R}^{2d}_{\!\scriptscriptstyle \diagup }})} C_V^{1/2} |a| \\&\quad + L_\Phi \Vert \eta \Vert _{\infty ,C_b({\mathbb {R}^{2d}_{\!\scriptscriptstyle \diagup }})}C_V^{1/2}\!\!\left[ \left( \!\int _{\mathbb {R}^d}\!\left( \int _{\mathbb {R}}|\xi '|\textrm{d}\sigma _{s,x'}(\xi ')\right) ^2\textrm{d}\mu (x')\!\right) ^{1/2}\right. \\&\qquad \left. + \left( \!\int _{\mathbb {R}^d}\!\left( \int _{\mathbb {R}}|\xi '|\textrm{d}\sigma _{s,x'}(\xi ')\right) ^2\textrm{d}\mu (x')\!\right) ^{1/2}\right] \\&\le 2L_\Phi \Vert \eta \Vert _\infty C_V^{1/2} |a| + 2L_\Phi \Vert \eta \Vert _{\infty ,C_b({\mathbb {R}^{2d}_{\!\scriptscriptstyle \diagup }})}C_V^{1/2}\left( \int _{\mathbb {R}^d}m_2[\sigma _{s,x'}]\textrm{d}\mu (x')\right) ^{1/2} \ \\&= 2L_\Phi \Vert \eta \Vert _\infty C_V^{1/2} |a| + 2L_\Phi \Vert \eta \Vert _{\infty ,C_b({\mathbb {R}^{2d}_{\!\scriptscriptstyle \diagup }})}C_V^{1/2} m_2^{1/2}[\gamma _s] <\infty \ , \end{aligned}$$

where in the third inequality we used Cauchy–Schwarz and (3.9a), and the final inequality follows from Jensen’s inequality. Then, since $t\mapsto V_t$ and $t \mapsto \eta _t$ are continuous, by the dominated convergence theorem we have that $II \rightarrow 0$ as $t \rightarrow s$. The limit $s \rightarrow t$ follows from an analogous argument. The remainder of the statement follows from the Cauchy–Lipschitz theory for ODEs. $\square$

The previous lemma guarantees we can consider, for $\mu$-a.e. $x\in \mathbb {R}^d$, a flow map $f_x[\sigma , r,\eta ]$, dependent on $\sigma ,r,\eta$, which is the solution to

4.8 $$\begin{aligned}&\frac{\hbox {d}}{\hbox {d}t}f_x[\sigma ,r,\eta ](t,u) = \mathcal {X}[\sigma ,r,\eta ](t,f_x[\sigma ,r,\eta ](t,u), x)\ , \\&f_x[\sigma ,r,\eta ](0,u) = u \in \mathbb {R}\ , \nonumber \end{aligned}$$

In particular, for an initial time $s>0$, we write $f_x[\sigma ,r,\eta ]((s,t),u)$, meaning that $f_x[\sigma ,r,\eta ](s,u) = u$. Before establishing the well-posedness of the nonlinear problem (graph-CE), we need to study some further properties of the flow.

#### Lemma 4.2

Let $V:[0,T]\times L^2_\mu (\mathbb {R}^d) \rightarrow \mathcal {V}^{as}({\mathbb {R}^{2d}_{\!\scriptscriptstyle \diagup }})$ be continuous in time and such that it satisfies (3.9a)–(3.9b). Let $\sigma \in C([0,T], \mathcal {P}^{\mu }_2(\mathbb {R}{\times \mathbb {R}^d}))$ with ${\pi _1}_\#\sigma =\gamma$ and $(r,\eta )$ be the solution of (Euler Co-NCL). The flow map $f_x[\sigma ,r,\eta ]$ defined in (4.8) satisfies the properties below.

1. Linear growth: for all $T>0$, there exists a constant $\widetilde{C}=C(\eta _0,m_2[\gamma ]) > 0$ such that
  $$ |f_x[\sigma ,r,\eta ](t,u)| \le e^{\widetilde{C}T}(1 + |u|), $$
  for any $$t\in [0,T]$$, $$u\in \mathbb {R}$$, $$\mu $$-a.e. $$x\in \mathbb {R}^d$$.
2. Lipschitz in *u*: there exists a constant $\bar{C}=C(\eta _0,V,\Phi ) > 0$ such that for any $t\in [0,T]$ and any $u,\ell \in \mathbb {R}$
  $$ |f_x[\sigma ,r,\eta ](t,u) - f_x[\sigma , r,\eta ](t,\ell )| \le e^{\bar{C}T}|u-\ell | . $$
3. Time continuity: the map $t \mapsto f_x[\sigma ,r,\eta ](t,\cdot )$ is continuous.

#### Proof

We begin by proving (1). By definition of the flow, we have

$$\begin{aligned} f_x[\sigma ,r,\eta ](t,u) = u + \int _0^t\mathcal {X}[\sigma ,r,\eta ](s,f_x[\sigma ,r,\eta ](s,u),x) \textrm{d}s \ . \end{aligned}$$

By means of (i), (ii) in Definition 2.2,

$$\begin{aligned} \big |\mathcal {X}[\sigma , r,\eta ]&(s,f_x[\sigma ,r,\eta ](s,u),x)\big | \\&\le \int _{\mathbb {R}\times \mathbb {R}^d\backslash \{x\}} \bigg |\Phi (f_x[\sigma ,r,\eta ](s,u),\xi ';V_s[r](x,x'))\eta _s(x,x')\bigg |\textrm{d}\sigma _s(\xi ',x') \\&\le L_{\Phi }\Vert \eta \Vert _{\infty , C_b({\mathbb {R}^{2d}_{\!\scriptscriptstyle \diagup }})} C_V^{1/2}\bigg (|f_x[\sigma ,r,\eta ](s,u)|\! +\! \left( \int _{\mathbb {R}^d}\!\!m_1^2[\sigma _{s,x'}]\textrm{d}\mu (x')\right) ^{1/2} \!\bigg ) \\&\le L_{\Phi }\Vert \eta \Vert _{\infty , C_b({\mathbb {R}^{2d}_{\!\scriptscriptstyle \diagup }})} C_V^{1/2}\bigg (|f_x[\sigma ,r,\eta ](s,u)| + m_2[\gamma _s]^{1/2}\bigg ) \\&\le L_{\Phi }\Vert \eta \Vert _{\infty , C_b({\mathbb {R}^{2d}_{\!\scriptscriptstyle \diagup }})} C_V^{1/2}\bigg (|f_x[\sigma ,r,\eta ](s,u)| + m_2[\gamma _*]^{1/2}\bigg )\ \\&\le L_\Phi \left( \Vert \eta _0\Vert _{\infty } + C_\omega \right) C_V^{1/2}\bigg (|f_x[\sigma ,r,\eta ](s,u)| + m_2[\gamma _*]^{1/2}\bigg ) \\&\le {\widetilde{C}}(1 + |f_x[\sigma ,r,\eta ](s,u)|)\ , \end{aligned}$$

for a constant $\widetilde{C}=C(\eta _0,m_2(\gamma ))$, where we used (3.12) in the penultimate inequality. An application of Grönwall’s inequality yields

$$\begin{aligned} |f_x[\sigma ,r,\eta ](t,u)| \le e^{\widetilde{C}T}(1 + |u|). \end{aligned}$$

To prove (2), consider $u,\ell \in \mathbb {R}$ and use (4.6) from Lemma 4.1 in order to have

$$\begin{aligned}&|f_x[\sigma , r,\eta ](t,u) - f_x[\sigma , r,\eta ](t,\ell )|\\&\le |u - \ell |\\&\quad + \int _0^t|\mathcal {X}[\sigma , r,\eta ](s,f_x[\sigma , r,\eta ](s,u),x) - \mathcal {X}[\sigma , r,\eta ](s,f_x[\sigma , r,\eta ](s,\ell ),x)|\textrm{d}s \\&\le |u-\ell |\\&\quad + L_\Phi C_V^{1/2} \Vert \eta \Vert _{\infty , C_b({\mathbb {R}^{2d}_{\!\scriptscriptstyle \diagup }})} \int _0^t|f_x[\sigma , r,\eta ](s,\ell ) - f_x[\sigma , r,\eta ](s,u)| \textrm{d}s . \end{aligned}$$

An application of Grönwall’s inequality yields

$$\begin{aligned} |f_x[\sigma , r,\eta ](t,u) - f_x[\sigma , r,\eta ](t,\ell )|&\le \exp \left( L_\Phi C_V^{1/2} \Vert \eta \Vert _{\infty , C_b({\mathbb {R}^{2d}_{\!\scriptscriptstyle \diagup }})} T\right) |u-\ell | \ , \\&\le \exp \left( L_\Phi C_V^{1/2} \left( \Vert \eta _0\Vert _{\infty } + C_\omega \right) T\right) |u-\ell | \\&\le e^{\bar{C}T}|u - \ell | \end{aligned}$$

for a constant $\bar{C}=C(\eta _0,V,\Phi ) > 0$, which concludes the proof of (2). The final statement follows from the definition of the flow and the time continuity of $\mathcal {X}[\sigma , r,\eta ]$ proved in Lemma 4.1. $\square$

The flow map depends on a probability measure $\sigma$ and a given co-evolving graph $\mathcal {G}_t(\mu ,\eta _t[r])$. To conclude our argument, we estimate the difference of the flow maps depending on different measures, $\sigma ^1,\ \sigma ^2$. To this end, let us consider the space $C([0,T], \mathcal {P}^\mu _2(\mathbb {R}\times \mathbb {R}^d))$ equipped with the metric

$$ \mathcal {D}_{\mu ,d}(\nu ^1,\nu ^2):=\sup _{t\in [0,T]}L^2_\mu d_2(\nu ^1_t,\nu ^2_t) = \sup _{t\in [0,T]} \left( \int _{\mathbb {R}^d} d^2_2(\nu ^1_{t,x'},\nu ^2_{t,x'})\textrm{d}\mu (x')\right) ^{1/2}, $$

for $\nu ^1,\nu ^2 \in C([0,T], \mathcal {P}^\mu _2(\mathbb {R}\times \mathbb {R}^d))$, so that $(C([0,T], \mathcal {P}^\mu _2(\mathbb {R}\times \mathbb {R}^d)), \mathcal {D}_{\mu ,d})$ is a complete metric space, see Mikolas (2024) (Proposition A.0.1) for a similar proof. We obtain the following estimate.

#### Lemma 4.3

Let $\sigma ^i \in C([0,T],\mathcal {P}^\mu _2(\mathbb {R}\times \mathbb {R}^d))$, with ${\pi _1}_\#\sigma ^i=\gamma ^i$ and $(r,\eta )$ be the solution of (Euler Co-NCL) with the initial condition $(r_0,\eta _0)$. Let $V:[0,T]\times L^2_\mu (\mathbb {R}^d) \rightarrow \mathcal {V}^{as}({\mathbb {R}^{2d}_{\!\scriptscriptstyle \diagup }})$ be continuous in time satisfying (3.9a) and (3.9b). Consider two flows $f^i_x[\sigma ^i, r, \eta ]$, for $\mu$-a.e. $x \in \mathbb {R}^d$, solutions to (4.8), for $i =1,2$. Then, it holds

4.9 $$\begin{aligned} |f^1_x[\sigma ^1,r,\eta ](t,u) - f_x^2[\sigma ^2,r,\eta ](t,u)|^2\le \bar{C}_TT^2\mathcal {D}_{\mu ,d}^2(\sigma ^1,\sigma ^2), \end{aligned}$$

where $\bar{C}_T=L_\Phi ^2\left( \Vert \eta _0 \Vert _{\infty }+C_\omega \right) ^2 C_V\exp \{ L_\Phi ^2 \left( \Vert \eta _0 \Vert _{\infty }+C_\omega \right) ^2 C_V \ T^2\}$. Consequently, for any measure $\nu \in \mathcal {P}_2^\mu (\mathbb {R}\times \mathbb {R}^d)$, with disintegration $\nu = \int _{\mathbb {R}^d}\nu _x \textrm{d}\mu (x)$, we have

4.10 $$\begin{aligned} \mathcal {D}_{\mu ,d}(f^1[\sigma ^1,r,\eta ]_\# \nu , f^2[\sigma ^2,r,\eta ]_\# \nu ) \le \tilde{C}_TT \mathcal {D}_{\mu ,d}(\sigma ^1,\sigma ^2) \, \end{aligned}$$

where $\tilde{C}_T=\bar{C}_T^{\frac{1}{2}}$ from above.

#### Proof

To improve the readability, we use the shorthand notation $f^i_x:= f^i_x[\sigma ^i,r,\eta ]$, for $i=1,2$. Let us start by estimating the difference of the two flows:

$$\begin{aligned} |f^1_x(t,u) - f_x^2(t,u)|&= \bigg |\int _0^t \int _{\mathbb {R}\times \mathbb {R}^d\backslash \{x\}} \Phi (f_x^1(s,u),\xi ';V_s[r_s](x,x'))\eta _s(x,x')\textrm{d}\sigma ^1_s(\xi ',x') \\&\qquad - \int _{\mathbb {R}\times \mathbb {R}^d\backslash \{x\}} \Phi (f_x^2(s,u),\xi ';V_s[r_s](x,x'))\eta _s(x,x')\textrm{d}\sigma ^2_s(\xi ',x') \textrm{d}s \bigg | \\&\le \int _0^t\bigg |\int _{\mathbb {R}\times \mathbb {R}^d\backslash \{x\}}\bigg ( \Phi (f_x^1(s,u),\xi ';V_s[r](x,x'))\eta _s(x,x') \\&\qquad - \Phi (f_x^2(s,u),\xi ';V_s[r_s](x,x'))\eta _s(x,x')\bigg )\textrm{d}\sigma _s^1(\xi ',x')\bigg | \textrm{d}s \\&\qquad + \int _0^t \bigg |\int _{\mathbb {R}\times \mathbb {R}^d\backslash \{x\}}\Phi (f_x^2(s,u),\xi ';V_s[r_s](x,x'))\eta _s(x,x')\textrm{d}(\sigma _s^1-\sigma ^2_s)(\xi ',x') \bigg |\textrm{d}s \\&=: I + II. \end{aligned}$$

We bound the term *I* analogous calculation to the ones for (3.10) to obtain

$$\begin{aligned} |I|&= \int _0^t\bigg | \int _{\mathbb {R}\times \mathbb {R}^d\backslash \{x\}}\!\!\!\bigg ( \Phi (f_x^1(s,u),\xi ';V_s[r_s](x,x')) \! \\&\qquad -\! \Phi (f_x^2(s,u),\xi ';V_s[r_s](x,x'))\bigg ) \eta _s(x,x') \textrm{d}\sigma _s^1(\xi ',x')\bigg | \textrm{d}s \\&\le L_\Phi \Vert \eta \Vert _{\infty ,C_b({\mathbb {R}^{2d}_{\!\scriptscriptstyle \diagup }})}\int _0^t\int _{\mathbb {R}^d}|V_s[r_s](x,x')||f_x^1(s,u)-f_x^2(s,u)|\textrm{d}\mu (x') \\&\le L_\Phi \Vert \eta \Vert _{\infty ,C_b({\mathbb {R}^{2d}_{\!\scriptscriptstyle \diagup }})} C_V^{1/2} \int _0^t|f_x^1(s,u)-f^2_x(s,u)|\textrm{d}s, \end{aligned}$$

whence

$$ |I|^2 \le L_\Phi ^2 \Vert \eta \Vert ^2_{\infty ,C_b({\mathbb {R}^{2d}_{\!\scriptscriptstyle \diagup }})} C_V \ T\int _0^t|f_x^1(s,u)-f^2_x(s,u)|^2\textrm{d}s . $$

On the other hand, for *II*, we have

4.11 $$\begin{aligned} {\begin{matrix} |II | & \le \int _{0}^t\int _{\mathbb {R}^d} \bigg |\int _{\mathbb {R}} \Phi (f_x^2(s,u),\xi ';V_s[r_s](x,x'))\eta _s(x,x')\textrm{d}(\sigma ^1_{s,x'}-\sigma ^2_{s,x'})(\xi ')\bigg | \textrm{d}\mu (x')\textrm{d}s \\ & \le \int _0^t\int _{\mathbb {R}^d} Lip[\Phi (f_x^2(s,u),\cdot ,V_s[r_s](x,x')\eta _s(x,x')]d_1(\sigma ^1_{s,x'},\sigma ^2_{s,x'})\textrm{d}\mu (x') \textrm{d}s \\ & \le L_\Phi \Vert \eta \Vert _{\infty ,C_b({\mathbb {R}^{2d}_{\!\scriptscriptstyle \diagup }})} \int _0^t\int _{\mathbb {R}^d}|V_s[r_s](x,x')|d_1(\sigma ^1_{s,x'},\sigma ^2_{s,x'})\textrm{d}\mu (x') \textrm{d}s \\ & \le L_\Phi \Vert \eta \Vert _{\infty ,C_b({\mathbb {R}^{2d}_{\!\scriptscriptstyle \diagup }})}C_V^{1/2} \int _0^t\left( \int _{\mathbb {R}^d}d^2_1(\sigma ^1_{s,x'},\sigma ^2_{s,x'})\textrm{d}\mu (x')\right) ^{1/2} \textrm{d}s \\ & \le L_\Phi \Vert \eta \Vert _{\infty ,C_b({\mathbb {R}^{2d}_{\!\scriptscriptstyle \diagup }})}C_V^{1/2}\int _0^t L^2_\mu d_2(\sigma ^1_{s},\sigma ^2_{s}) \textrm{d}s\\ & \le L_\Phi \Vert \eta \Vert _{\infty ,C_b({\mathbb {R}^{2d}_{\!\scriptscriptstyle \diagup }})}C_V^{1/2} T\mathcal {D}_{\mu ,d}(\sigma ^1,\sigma ^2) \end{matrix}} \end{aligned}$$

where the second and third inequalities follow from the definition by duality of the 1-Wasserstein distance and (4.7), the fourth inequality follows from Cauchy–Schwarz. Squaring both sides and taking the supremum over time yield

$$\begin{aligned} |II|^2&\le L_\Phi ^2 \Vert \eta \Vert ^2_{\infty ,C_b({\mathbb {R}^{2d}_{\!\scriptscriptstyle \diagup }})}C_V \mathcal {D}_{\mu ,d}^2(\sigma ^1,\sigma ^2)T^2 \ . \end{aligned}$$

Thus, collecting all the terms we get that

$$\begin{aligned} |f^1_x(t,u) - f_x^2(t,u)|^2 \le \&L_\Phi ^2 \Vert \eta \Vert ^2_{\infty ,C_b({\mathbb {R}^{2d}_{\!\scriptscriptstyle \diagup }})} C_V \ T\int _0^t |f_x^1(s,u)-f^2_x(s,u)|^2\textrm{d}s \\&+ L_\Phi ^2 \Vert \eta \Vert ^2_{\infty ,C_b({\mathbb {R}^{2d}_{\!\scriptscriptstyle \diagup }})}C_V\mathcal {D}_{\mu ,d}^2(\sigma ^1,\sigma ^2)T^2, \end{aligned}$$

and an application of Grönwall’s inequality yields

4.12 $$\begin{aligned} {\begin{matrix} |f^1_x(t,u) - f_x^2(t,u)|^2 & \le L_\Phi ^2\Vert \eta \Vert ^2_{\infty ,C_b({\mathbb {R}^{2d}_{\!\scriptscriptstyle \diagup }})}C_V\mathcal {D}_{\mu ,d}^2(\sigma ^1,\sigma ^2)T^2\exp \left\{ L_\Phi ^2 \Vert \eta \Vert ^2_{\infty ,C_b({\mathbb {R}^{2d}_{\!\scriptscriptstyle \diagup }})} C_V \ T^2\right\} \\ & \le \bar{C}_TT^2\mathcal {D}_{\mu ,d}^2(\sigma ^1,\sigma ^2), \end{matrix}} \end{aligned}$$

where $\bar{C}_T=L_\Phi ^2\left( \Vert \eta _0 \Vert _{\infty }+C_\omega \right) ^2 C_V\exp \left\{ L_\Phi ^2 \left( \Vert \eta _0 \Vert _{\infty }+C_\omega \right) ^2 C_V \ T^2\right\}$. The first statement is therefore proven. To obtain the second estimate, consider the transference plan $\Pi _{t,x} = (f_x^1 \times f_x^2)_\# \nu _x$. For $\mu$-a.e. $x \in \mathbb {R}^d$, we have that

$$\begin{aligned} d^2_2({f_x}^1_\#{\nu _x}, {f_x}^2_\#{\nu _x})&\le \int _{\mathbb {R}\times \mathbb {R}} |a-b|^2 \textrm{d}\Pi _{t,x}(a,b) \\&\le \int _{\mathbb {R}}|f_x^1(t,u) - f_x^2(t,u)|^2 \textrm{d}\nu _x(u) \\&\le \bar{C}_TT^2\mathcal {D}_{\mu ,d}^2(\sigma ^1,\sigma ^2), \end{aligned}$$

where the final inequality follows from (4.12). Integrating with respect to $\mu$, taking the square root on both sides and the supremum in time gives

$$ \mathcal {D}_{\mu ,d} (f^1_\#\nu ,f^2_\#\nu ) \le \tilde{C}_TT \mathcal {D}_{\mu ,d}(\sigma ^1,\sigma ^2), $$

where $$\tilde{C}_T=\bar{C}_T^{\frac{1}{2}}$$. $$\square $$

In order to prove well-posedness of (graph-CE) through its disintegrated version, we first need to study well-posedness for solutions of a linear version of this problem, namely given $\sigma \in C([0,T];\mathcal {P}_2^\mu (\mathbb {R}\times \mathbb {R}^d))$,

4.13 $$\begin{aligned} {\begin{matrix} & \partial _t \tilde{\sigma }_{t,x} + \partial _{\xi }(\tilde{\sigma }_{t,x}\mathcal {X}[\sigma ,\rho ,\eta ]) = 0, \\ & \tilde{\sigma }_{0,x} = \bar{\sigma }_x \in \mathcal {P}(\mathbb {R}), \end{matrix}} \end{aligned}$$

for $\mu$-a. e. $x\in \mathbb {R}^d$ and $\bar{\sigma }=\int _{\mathbb {R}^d}\bar{\sigma }_x\textrm{d}\mu (x)$. We prove existence and uniqueness of solutions to (4.13) by duality, that is, we first consider classical solutions to the transport equation, $\mu \text {-a.e. } x \in \mathbb {R}^d$:

4.14 $$\begin{aligned} {\left\{ \begin{array}{ll} \partial _t \varphi + \partial _\xi \varphi \mathcal {X}[\sigma , r, \eta ](t,\xi ,x) = 0, \qquad t<T, \, (\xi ,x)\in \mathbb {R}\times \mathbb {R}^d\\ \varphi (T,\xi ,x) = \psi (\xi ,x) \in C^1_c(\mathbb {R}\times \mathbb {R}^d). \end{array}\right. } \end{aligned}$$

The method of characteristics provides the following result; see (Evans 2010, Chapter 3) for further details.

#### Proposition 4.1

Let ${\sigma }\in C([0,T],\mathcal {P}^\mu _2(\mathbb {R}\times \mathbb {R}^d))$. The problem (4.14) has a unique classical solution $\varphi (t,u,x) = \psi (f_{x}[\sigma ,r,\eta ]((0,t), u)) \in C^1_c([0,T]\times \mathbb {R})$, for $\mu$-a.e. $x \in \mathbb {R}^d$, where $f_x$ is as in (4.8).

For a given $\sigma \in C([0,T];\mathcal {P}_2^\mu (\mathbb {R}\times \mathbb {R}^d))$, we can construct explicit distributional solutions for (4.13) as in the following theorem.

#### Proposition 4.2

Let $\tilde{\sigma }_{0} \in \mathcal {P}^\mu _2(\mathbb {R}\times \mathbb {R}^d)$ such that $\tilde{\sigma }_0 = \int _{\mathbb {R}^d}\tilde{\sigma }_{0,x'}\textrm{d}\mu (x')$ and $\sigma \in C([0,T], \mathcal {P}^\mu _2(\mathbb {R}\times \mathbb {R}^d))$. Assume $V:[0,T]\times L^2_\mu (\mathbb {R}^d) \rightarrow \mathcal {V}^{as}({\mathbb {R}^{2d}_{\!\scriptscriptstyle \diagup }})$ satisfies (3.9a), (3.9b) and that it is continuous in time. Let $(r,\eta )$ be a solution to (Euler Co-NCL). Then, $\tilde{\sigma }_{t,x} = f_x[\sigma ,r,\eta ](t,x,\cdot )_\# \tilde{\sigma }_{0,x}$ is the unique distributional solution to (4.13), for $\mu$-a.e. $x \in \mathbb {R}^d$, where $f_x$ is defined in (4.8).

#### Proof

First, we notice that by direct computation it can be checked that $\tilde{\sigma }_{t,x} = f_x[\sigma ,\rho ,\eta ](t,\cdot )_\# \tilde{\sigma }_{0,x}$ satisfies the equation in the sense of distributions, i.e. (4.5), for $\mu$-a.e. $x \in \mathbb {R}^d$. Secondly, by the linearity of (4.13), we can show uniqueness for solutions with $\tilde{\sigma }_{0} = 0$. By choosing $\varphi (t,\xi ,x) = \psi (f_x[\sigma ,\rho ,\eta ]((0,t),\xi ))$ as a test function in (4.5), we obtain

$$\begin{aligned} \int _{\mathbb {R}} \varphi (T,\xi ,x) \textrm{d}\tilde{\sigma }_{T,x}(\xi ) = \int _{\mathbb {R}}\psi (\xi ,x)\textrm{d}\tilde{\sigma }_{T,x}(\xi ) = 0 \ , \ \mu \text {-a.e. } x \in \mathbb {R}^d, \end{aligned}$$

for all $\psi \in C^1_c(\mathbb {R}\times \mathbb {R}^d)$, whence $\tilde{\sigma }_{T,x}=0$ and we obtain uniqueness. $\square$

We are ready to prove the well-posedness of (graph-CE). For any $\nu \in \mathcal {P}^\mu _2(\mathbb {R}\times \mathbb {R}^d)$ with disintegration $\nu = \int _{\mathbb {R}^d}\nu _x \textrm{d}\mu (x)$, $x \in \mathop {\textrm{supp}}\limits \mu$ and the solution $(r,\eta )$ of (Euler Co-NCL), we define the solution map $\mathcal {S}^{\nu ,r,\eta }:C([0,T], \mathcal {P}^\mu _2(\mathbb {R}\times \mathbb {R}^d)) \rightarrow C([0,T], \mathcal {P}^\mu _2(\mathbb {R}\times \mathbb {R}^d))$ as

4.15 $$\begin{aligned} \mathcal {S}^{\nu ,r,\eta }[\sigma ](t) :=\int _{\mathbb {R}^d} f_x[\sigma ,r,\eta ](t,\cdot )_\# \nu _x\textrm{d}\mu (x) \ , \quad \sigma \in \mathcal {P}_2^\mu (\mathbb {R}\times \mathbb {R}^d), \end{aligned}$$

where $f_x[\sigma ,r,\eta ]$ is defined in (4.8). Note that the solution map is well defined due to the previous results. We denote the disintegration with respect to $\mu$ by $\mathcal {S}^{\nu ,r,\eta }_x[\sigma ](t):= f_x[\sigma ,r,\eta ](t,\cdot )_\# \nu _x$, for $\mu$-a.e. $x \in \mathbb {R}^d$. We prove well-posedness of the nonlinear problem (graph-CE) by another fixed-point argument.

#### Theorem 4.1

Let $V:[0,T]\times L^2_\mu (\mathbb {R}^d) \rightarrow \mathcal {V}^{as}({\mathbb {R}^{2d}_{\!\scriptscriptstyle \diagup }})$ be continuous in time and satisfy (3.9a) and (3.9b). Let $(r,\eta )$ be the solution of (Euler Co-NCL). Then, there exists a unique solution $\sigma \in C([0,T],\mathcal {P}^\mu _2(\mathbb {R}\times \mathbb {R}^d))$ to (graph-CE) with the initial condition $\nu \in \mathcal {P}^\mu _2(\mathbb {R}\times \mathbb {R}^d)$ given by $\sigma = \int _{\mathbb {R}^d}\sigma _{t,x}\textrm{d}\mu (x)$ where $\sigma _{t,x}=f_x[\sigma ,r,\eta ](t,\cdot )_\#\nu _x$ for $\mu$-a.e. $x \in \mathbb {R}^d$.

#### Proof

Let us consider the flow $f_x[\sigma ,\rho , \eta ]$ as defined in (4.8), for some $\sigma \in C([0,T],\mathcal {P}_2^\mu (\mathbb {R}\times \mathbb {R}^d))$. Note that by Proposition 4.2, we have that $\tilde{\sigma }_x = \mathcal {S}_x^{\nu ,r,\eta }[\sigma ]$ is the unique solution in $C([0,T],\mathcal {P}^\mu _2(\mathbb {R}\times \mathbb {R}^d))$ to the linear problem (4.13) with initial datum $\nu \in \mathcal {P}^\mu _2(\mathbb {R}\times \mathbb {R}^d)$. Thus, to obtain existence and uniqueness of solution to the nonlinear problem (graph-CE), it suffices to show the solution map $\mathcal {S}^{\nu ,r,\eta }[\sigma ]$ has a unique fixed-point. Using the disintegrated version of the solution map, $\mathcal {S}^{\nu ,r,\eta }_x[\sigma ](t)$, and Lemma 4.3 we have, for any $\sigma ^1,\sigma ^2 \in C([0,T],\mathcal {P}_2^\mu (\mathbb {R}\times \mathbb {R}^d))$,

$$ \mathcal {D}_{\mu ,d}(\mathcal {S}^{\nu ,r,\eta }[\sigma ^1], \mathcal {S}^{\nu ,r,\eta }[\sigma ^2]) \le \tilde{C}_T T\mathcal {D}_{\mu ,d}(\sigma ^1,\sigma ^2), $$

where $\tilde{C}_T>0$ is the constant from (4.10) and it is of the form $\tilde{C}_T = C \exp \{\alpha T^2\}$. Then, for $T < \tilde{C}_T^{-1}$ the solution map is a contraction. By the Banach fixed-point theorem, we can conclude there exists a unique solution to (graph-CE) in $C([0,T],\mathcal {P}_2^\mu (\mathbb {R}\times \mathbb {R}^d))$ for $T < T^*$, where $T^*$ is such that $\tilde{C}_{T*}{T^*}=1$. The solution can be extended using a standard iteration procedure so that the time interval does not depend on any $T^*$, see, e.g. the proof of Theorem 2.1 in Esposito and Mikolás (2024). $\square$

#### Theorem 4.2

Assume $V:[0,T]\times L^2_\mu (\mathbb {R}^d) \rightarrow \mathcal {V}^{as}({\mathbb {R}^{2d}_{\!\scriptscriptstyle \diagup }})$ to be continuous in time and to satisfy (3.9a), (3.9b). Let $(r,\eta )$ be the solution to (Euler Co-NCL) with the initial datum $(r_0, \eta _0)\in L^2_\mu (\mathbb {R}^d) \times C_b({\mathbb {R}^{2d}_{\!\scriptscriptstyle \diagup }})$ and let $\sigma ^i \in C([0,T], \mathcal {P}_2^\mu (\mathbb {R}\times \mathbb {R}^d))$ be the solutions to the corresponding (graph-CE) with initial conditions $\tilde{\sigma }^i_0\in \mathcal {P}^\mu _2(\mathbb {R}\times \mathbb {R}^d)$ for $i=1,2$. The following stability estimate holds

$$ \mathcal {D}_{\mu ,d}(\sigma ^1,\sigma ^2) \le \sqrt{2}e^{C(T)} L^2_\mu d_2(\tilde{\sigma }^1_0,\tilde{\sigma }^2_0). $$

#### Proof

Let us write $f^i_x = f^i_x[\sigma ^i,r,\eta ]$, for $i=1,2$. Then, we have that, for $\mu$-a.e. $x\in \mathbb {R}^d$

$$\begin{aligned} d_2^2(\sigma ^1_{t,x}, \sigma ^2_{t,x})&= d_2^2(f^1_x[\sigma ^1,r,\eta ](t,\cdot )_\#\tilde{\sigma }^1_{0,x}, f^2_x[\sigma ^2,r,\eta ](t,\cdot )_\#\tilde{\sigma }^2_{0,x}) \\&\le 2 d_2^2(f^1_x(t)_\#\tilde{\sigma }^1_{0,x}, f^2_x(t)_\#\tilde{\sigma }^1_{0,x}) + 2 d_2^2(f^2_x(t)_\#\tilde{\sigma }^1_{0,x}, f^2_x(t)_\#\tilde{\sigma }^2_{0,x}) \\&\le \mathcal {C}(T) \left( \int _0^t L^2_\mu d_2({\sigma ^1_s}, {\sigma ^2_s})\textrm{d}s \right) ^2 + 2d_2^2(f^2_x(t)_\#\tilde{\sigma }^1_{0,x}, f^2_x(t)_\#\tilde{\sigma }^2_{0,x}) \ , \end{aligned}$$

where $\mathcal {C}(T)=L_\Phi ^2\left( \Vert \eta _0 \Vert _{\infty }+C_\omega \right) ^2 C_V\exp \{ L_\Phi ^2 \left( \Vert \eta _0 \Vert _{\infty }+C_\omega \right) ^2 C_V \ T^2\}$ is the constant obtained as in (4.12) if we do not take the supremum in time in (4.11). To bound the second term on the right-hand side, we proceed as follows. Let $\Pi _{0,x} \in \Gamma (\tilde{\sigma }_{0,x}^1,\tilde{\sigma }_{0,x}^2)$ be an optimal transference plan for $\tilde{\sigma }_{0,x}^1$ and $\tilde{\sigma }_{0,x}^2$. Then for $\mu$-a.e. $x\in \mathbb {R}^d$ we have that

$$\begin{aligned} d_2^2(f^2_x(t)_\#\tilde{\sigma }^1_{0,x}, f^2_x(t)_\#\tilde{\sigma }^2_{0,x})&\le \int _{\mathbb {R}\times \mathbb {R}}|f^2_x(t,u) - f^2_x(t,v)|^2\textrm{d}\Pi _{0,x}(u,v) \\&\le e^{\bar{C}_T}\int _{\mathbb {R}\times \mathbb {R}}|u-v|^2\textrm{d}\Pi _{0,x}(u,v) \\&\le e^{\bar{C}_T}d_2^2(\tilde{\sigma }_{0,x}^1,\tilde{\sigma }_{0,x}^2) \ , \end{aligned}$$

where the second inequality follows from (2) of Lemma 4.2 and $\bar{C}=C(\eta _0,V,\Phi )>0$. Summarising, we get

$$ d_2^2(\sigma ^1_{t,x}, \sigma ^2_{t,x}) \le \mathcal {C}(T) \left( \int _0^t L^2_\mu d_2({\sigma ^1_s}, {\sigma ^2_s})\textrm{d}s \right) ^2 + 2e^{\bar{C}_T}d_2^2(\tilde{\sigma }_{0,x}^1,\tilde{\sigma }_{0,x}^2), $$

whence, by integrating w.r.t. $\mu$ and taking the square root, we obtain

$$ L^2_\mu d_2(\sigma ^1_t,\sigma ^2_t)\le \bar{\mathcal {C}}(T)\int _0^t L^2_\mu d_2({\sigma ^1_s}, {\sigma ^2_s})\textrm{d}s + \sqrt{2}e^{\bar{C}_T} L^2_\mu d_2(\sigma _0^1,\sigma _0^2), $$

where $\bar{\mathcal {C}}(T)=\sqrt{\mathcal {C}(T)}$ An application of Grönwall’s inequality yields

$$ L^2_\mu d_2(\sigma ^1_t,\sigma ^2_t)\le \sqrt{2}e^{\bar{C}_T+\bar{\mathcal {C}}(T)T} L^2_\mu d_2(\sigma _0^1,\sigma _0^2), $$

hence the result. $\square$

### Contraction in $L^2_\mu d_2$

In this section, we focus on proving contraction in $L^2_\mu$ for solutions of (graph-CE), as this can allow to study the long-time behaviour of the $\mu$-monokinetic solutions and hence the long-time asymptotics for evolutions on graphs. We present the result in the simpler case of $\Phi \equiv \Phi _{upwind}$ and *V* and $\omega$ not depending on $r \in L^2_\mu (\mathbb {R}^d)$, so that the system (Euler Co-NCL) decouples, i.e. the graph is no longer co-evolving but only evolving.

#### Proposition 4.3

Fix $\Phi \equiv \Phi _{upwind}$. Let $\sigma ^i \in C([0,T], \mathcal {P}^\mu _2(\mathbb {R}\times \mathbb {R}^d))$ be $\mu$-monokinetic solutions of (graph-CE), with initial data $\sigma ^i_0 = \delta _{r^i_0(\cdot )} \otimes \mu$, where $(r^i,\eta )$ are solutions to (Euler Co-NCL) starting from $(r^i_0,\bar{\eta }) \in L^2_\mu (\mathbb {R}^d)\times C_b({\mathbb {R}^{2d}_{\!\scriptscriptstyle \diagup }})$, for $i=1,2$, and $\bar{\eta }>0$. Let $V:[0,T] \rightarrow \mathcal {V}^{as}({\mathbb {R}^{2d}_{\!\scriptscriptstyle \diagup }})$ be an antisymmetric velocity field which is continuous in time and satisfies (3.9a) and (3.9b), and let $\omega :[0,T] \times {\mathbb {R}^{2d}_{\!\scriptscriptstyle \diagup }}\rightarrow \mathbb {R}$ satisfy $({\boldsymbol{\omega }}1)$–$({\boldsymbol{\omega }}4)$, and $\omega \ge \omega _*>0$. Assume,

4.16 $$\begin{aligned} \lambda := \inf _{t\in [0,T]}\inf _{x \in \mathbb {R}^d}\int _{\mathbb {R}^d}V_t(x,x')\eta _t(x,x')\textrm{d}\mu (x') > 0. \end{aligned}$$

Then, for any $t\in [0,T]$,

$$ L^2_\mu d_2(\sigma ^1_t, \sigma ^2_t) \le e^{- 2\lambda t}L^2_\mu d_2(\sigma ^1_0, \sigma ^2_0). $$

#### Proof

First, we notice that $\eta _t>0$ since $\eta _0=\bar{\eta }>0$ and $\omega \ge \omega _*>0$, by means of the explicit solution form of $\eta$ given in (3.11) (see Lemma 5.4). Let $\sigma ^i_{t,x} = \delta _{r^i_t(x)}\in \mathcal {P}_2(\mathbb {R})$ be the disintegration of $\sigma ^i$ with respect to $\mu$ for $i=1,2$. Then, for $\mu$-a.e. $x \in \mathbb {R}^d$, $\Pi _{t,x} = \delta _{r^1_t(x)}\otimes \delta _{r^2_t(x)}$ is the optimal transference plan between $\sigma ^1_{t,x}$ and $\sigma ^2_{t,x}$, so that

$$ \left( L^2_\mu d_2(\sigma ^1_t,\sigma ^2_t)\right) ^2 = \int _{\mathbb {R}^d}d^2_2(\sigma ^1_{t,x},\sigma ^2_{t,x})\textrm{d}\mu (x) = \int _{\mathbb {R}^d}|r^1_t(x) - r^2_t(x)|^2\textrm{d}\mu (x). $$

Since $r^1,r^2 \in {{\,\textrm{AC}\,}}([0,T],L^2_\mu (\mathbb {R}^d))$ are solutions to (Euler Co-NCL), by exploiting the dominated convergence theorem, we obtain the following estimate:

$$\begin{aligned} \frac{\hbox {d}}{\hbox {d}t}&\left( L^2_\mu d_2(\sigma ^1_t,\sigma ^2_t)\right) ^2\\&=\int _{\mathbb {R}^d}\frac{\hbox {d}}{\hbox {d}t}|r_t^1(x) - r_t^2(x)|^2\textrm{d}\mu (x) \\&= \ -2\int _{\mathbb {R}^d}(r^1_t(x) - r^2_t(x)) \\&\quad \times \bigg (r^1_t(x) \int _{\mathbb {R}^d\backslash \{x\}}V^+_t(x,x')\eta _t(x,x') \textrm{d}\mu (x') - \int _{\mathbb {R}^d}r^1_t(x') V^-_t(x,x') \eta _t(x,x') \textrm{d}\mu (x') \\&\qquad \quad - r^2_t(x) \int _{\mathbb {R}^d\backslash \{x\}}V^+_t(x,x')\eta _t(x,x') \textrm{d}\mu (x') + \int _{\mathbb {R}^d}r^2_t(x') V^-_t(x,x') \eta _t(x,x') \textrm{d}\mu (x')\bigg ) \\&= -2\int _{\mathbb {R}^d}(r^1_t(x) - r^2_t(x))^2\int _{\mathbb {R}^d\backslash \{x\}}V^+_t(x,x')\eta _t(x,x')\textrm{d}\mu (x')\textrm{d}\mu (x) \\&\quad + 2 \int _{\mathbb {R}^d}\int _{\mathbb {R}^d\backslash \{x\}}(r^1_t(x) - r^2_t(x))(r^1_t(x') - r^2_t(x'))V^-_t(x,x')\eta _t(x,x') \textrm{d}\mu (x') \textrm{d}\mu (x)\\&\le -2\int _{\mathbb {R}^d}(r^1_t(x) - r^2_t(x))^2\int _{\mathbb {R}^d\backslash \{x\}}V^+_t(x,x')\eta _t(x,x')\textrm{d}\mu (x')\textrm{d}\mu (x) \\&\quad + \int _{\mathbb {R}^d}\int _{\mathbb {R}^d\backslash \{x\}}[(r^1_t(x) - r^2_t(x))^2 + (r^1_t(x') - r^2_t(x'))^2]V^-_t(x,x')\eta _t(x,x') \textrm{d}\mu (x') \textrm{d}\mu (x)\\&= -2\int _{\mathbb {R}^d}(r^1_t(x) - r^2_t(x))^2\int _{\mathbb {R}^d\backslash \{x\}}V^+_t(x,x')\eta _t(x,x')\textrm{d}\mu (x')\textrm{d}\mu (x) \\&\quad +\int _{\mathbb {R}^d}(r^1_t(x) - r^2_t(x))^2\int _{\mathbb {R}^d\backslash \{x\}}V^-_t(x,x')\eta _t(x,x') \textrm{d}\mu (x') \textrm{d}\mu (x) \\&\quad + \int _{\mathbb {R}^d}(r^1_t(x) - r^2_t(x))^2\int _{\mathbb {R}^d\backslash \{x\}}V^+_t(x,x')\eta _t(x,x') \textrm{d}\mu (x') \textrm{d}\mu (x) \\&= -\int _{\mathbb {R}^d}(r^1_t(x) - r^2_t(x))^2\int _{\mathbb {R}^d\backslash \{x\}}V_t(x,x') \eta _t(x,x')\textrm{d}\mu (x')\textrm{d}\mu (x) \\&\le -\lambda \left( L^2_\mu d_2(\sigma ^1_t, \sigma ^2_t)\right) ^2 \ , \end{aligned}$$

where in the third equality we have used the antisymmetry of *V* and the symmetry of $\eta$. An application of Grönwall’s inequality concludes the proof. $\square$

Condition (4.16) does not seem to be reasonable since, even for fully connected graphs, i.e. with $\eta >0$ for all $t \ge 0$, it can only be satisfied if the velocity is positive for all vertices for any time—this means that all vertices must “give mass”to, at least, one neighbour. It is, therefore, difficult to find examples that fulfil this condition and that are of the form $V(x,x') = -\overline{\nabla }\phi (x,x') = -(\phi (x') - \phi (x))$ for some suitable, potentially solution dependent, function $\phi$. This motivates the alternative approach presented in Sect. 5 to obtain a possible long-time behaviour of solutions to (Euler Co-NCL). Providing a more reasonable condition in place of (4.16) is then an open problem.

## Long-Time Behaviour for Pointwise and Monotonic Velocities

In this section, we consider a specific class of velocities on the graph in order to define a certain order for the flow, helpful to establish a possible long-time behaviour for solutions of (Euler Co-NCL), for $\Phi \equiv \Phi _{upwind}$. We highlight two main features. First, velocities are *pointwise*, that is $V_t[r](x,x') = V_t(r_t(x),r_t(x'))$ for a solution $(r,\eta )$ to (Euler Co-NCL). Second, they are *monotonic*, intuitively meaning that the sign of the velocity, hence the flow, is determined by the amount of mass at the vertices.

### Definition 5.1

We say that a pointwise velocity $V: [0, T] \times L_\mu ^\infty (\mathbb {R}^d) \rightarrow \mathcal {V}^{as}({{\mathbb {R}^{2d}_{\!\scriptscriptstyle \diagup }}})$ is *monotonic* if, for $r \in L_\mu ^\infty (\mathbb {R}^d)$, any $t \ge 0$, and $\mu$-a.e. $x, x' \in \mathbb {R}^d$ we have that:

- $$r(x) > r(y)$$, then $$V_t^+(r(x), r(x')) > V_t^+(r(y), r(x'))$$;
- $$r(x) < r(y)$$, then $$V_t^-(r(x), r(x')) > V_t^-(r(y), r(x'))$$;
- $$r(x) = r(x')$$, then $$V_t(r(x), r(x')) =0$$.

For any $t\ge 0$, $V_t^+:= \max (V_t,0)$ and $V_t^-:=(-V_t)^+$ are the positive and negative parts of $V_t$, respectively.

Let us explain the meaning of the conditions in the previous definition. The first condition can be interpreted as saying that, if the mass is greater at a vertex $x\in \mathbb {R}^d$ than $y\in \mathbb {R}^d$, then the outflow from vertex *x* to its neighbours is greater than the outflow from *y*. Conversely, if the mass at vertex *x* is lower than the mass at vertex *y*, the inflow to *x* from its neighbours should be greater than the inflow to *y*. The rest of the conditions follow the same type of reasoning. This assumption on the velocity is similar on the upwind structure of (Euler Co-NCL), i.e. $\Phi \equiv \Phi _{upwind}$, although we now require that the direction depends on the quantity of mass. The pointwise nature of the velocities requires we should work in the $L^\infty _\mu$ setting. Furthermore, we stress that monotonic velocities ensure that the mass on the graph diffuses as we shall show in Sect. 5.2.

Our main example for such velocities is given by

5.1 $$\begin{aligned} V_t(r_t(x),r_t(x')) = -\overline{\nabla }\alpha (r_t)(x,x') = \alpha [r_t(x)] - \alpha [r_t(x')]\ , \end{aligned}$$

where $\alpha :{\mathbb {R}}\rightarrow \mathbb {R}$ is monotonically increasing and bounded such as $\alpha (x) = \frac{1}{1+ e^{-x}}$ for $x\in \mathbb {R}^d$. We remark that this type of velocity is different from the ones considered in the previous sections, which came from interaction and potential energies.

### $L^\infty$ Theory for (Euler Co-NCL)

Let us start by briefly adapting the theory developed in Sect. 3 to the $L^\infty _\mu$-setting. Focusing on the dynamics for $\eta$, the function $\omega :[0,T]\times L_\mu ^\infty (\mathbb {R}^d)\times {\mathbb {R}^{2d}_{\!\scriptscriptstyle \diagup }}\rightarrow \mathbb {R}$ will now satisfy the following version of assumption $({\boldsymbol{\omega }}1)$–$({\boldsymbol{\omega }}4)$.

$(\tilde{{\boldsymbol{\omega }}}1)$: the map $(x,y)\in {\mathbb {R}^{2d}_{\!\scriptscriptstyle \diagup }}\mapsto \omega _t[\cdot ](x,y)$ is continuous and $(t\mapsto \omega _t[\cdot ](\cdot ,\cdot ))\in L^1([0,T])$;
$(\tilde{{\boldsymbol{\omega }}}2)$: there exists a constant $L_\omega \ge 0$ such that for any $f,g \in L_\mu ^\infty (\mathbb {R}^d)$
  $$\begin{aligned} \sup _{t \in [0,T]}\sup _{(x,y) \in {\mathbb {R}^{2d}_{\!\scriptscriptstyle \diagup }}} | \omega _t[f](x,y) - \omega _t[g](x,y)|&\le L_\omega \Vert f - g \Vert _{L_\mu ^\infty (\mathbb {R}^d)}; \end{aligned}$$
$(\tilde{{\boldsymbol{\omega }}}3)$: $\omega$ is bounded, that is, there exists a constant $\tilde{C}_{\omega }>0$ such that
  $$\begin{aligned} \sup _{t \in [0,T]}\sup _{r \in L_\mu ^\infty (\mathbb {R}^d)}\sup _{(x,y) \in {\mathbb {R}^{2d}_{\!\scriptscriptstyle \diagup }}}\big |\omega _t[r](x,y)\big | \le \tilde{C}_{\omega }. \end{aligned}$$
$(\tilde{{\boldsymbol{\omega }}}4)$: The map $(x,y) \mapsto \omega _\cdot [\cdot ](x,y)$ is symmetric.

Although assumption $(\tilde{{\boldsymbol{\omega }}}4)$ is not needed to obtain the well-posedness, it is important both to obtain the form (3.1) for the divergence of the flux and to be able to apply the result in Esposito et al. (2023), Proposition 5.2. Our guiding example for the function $\omega$ is as in (3.3). Solutions of (Euler Co-NCL) in the $L^\infty _\mu$-setting are defined in Definition 3.1 for $p=\infty$. Well-posedness of (Euler Co-NCL) in the $L^\infty _\mu$-setting can be proven by following the same strategy as in Sect. 3, by exploiting the Banach fixed-point theorem, with $\mu \in \mathcal {P}(\mathbb {R}^d)$. Below we provide the statements without proofs, cf. Mikolas (2024) for further details.

#### Proposition 5.1

Let $\Phi$ be a jointly antisymmetric admissible flux interpolation, $r_0 \in L^\infty _{\mu }(\mathbb {R}^d)$, $\eta _0\in C_b({\mathbb {R}^{2d}_{\!\scriptscriptstyle \diagup }})$. Assume $\omega :[0,T]\times L^\infty _\mu (\mathbb {R}^d)\times {\mathbb {R}^{2d}_{\!\scriptscriptstyle \diagup }}\rightarrow \mathbb {R}$ is such that the map $(x,y)\in {\mathbb {R}^{2d}_{\!\scriptscriptstyle \diagup }}\mapsto \omega _t[\cdot ](x,y)$ is continuous and that, for a constant $C_{\omega }>0$,

5.2 $$\begin{aligned} \int _0^T \sup _{r \in L^\infty _\mu (\mathbb {R}^d)}\sup _{(x,y) \in {\mathbb {R}^{2d}_{\!\scriptscriptstyle \diagup }}}|\omega _s[r](x,y)| \textrm{d}s\le C_{\omega }\ . \end{aligned}$$

Suppose that $V:[0, T]\times L^\infty _\mu (\mathbb {R}^d) \rightarrow \mathcal {V}^{\textrm{as}}({\mathbb {R}^{2d}_{\!\scriptscriptstyle \diagup }})$ is pointwise and satisfies

5.3 $$\begin{aligned} \sup _{t\in [0,T]} \sup _{r \in L_\mu ^\infty (\mathbb {R}^d)}\sup _{(x,x') \in {\mathbb {R}^{2d}_{\!\scriptscriptstyle \diagup }}} |V_t(r_t(x),r_t(x'))| \le C_{V}\ , \end{aligned}$$

for some $C_{V}>0$. For a pair $(r,\eta ):[0,T]\rightarrow L^\infty _\mu (\mathbb {R}^d)\times C_b({\mathbb {R}^{2d}_{\!\scriptscriptstyle \diagup }})$ satisfying (), the following properties hold.

1. For $\mu$-a.e. $x \in \mathbb {R}^d$ and every $(x,y)\in {\mathbb {R}^{2d}_{\!\scriptscriptstyle \diagup }}$, the maps
  $$\begin{aligned}&t \mapsto \int _{\mathbb {R}^d\backslash \{x\}}\Phi (r_t(x),r_t(y); V_s(r_s(x),r_s(x')))\eta _s(x,x)\textrm{d}\mu (y) \in L^1([0,T])\ , \\&t\mapsto \omega _t[r](x,y) - \eta _t(x,y) \in L^1([0,T])\ , \end{aligned}$$
  and $$r \in L^\infty ([0,T];L^\infty _\mu (\mathbb {R}^d)), \eta \in L^\infty ([0,T],C_b({\mathbb {R}^{2d}_{\!\scriptscriptstyle \diagup }}))$$.
2. $$r \in AC([0,T];L^\infty _\mu (\mathbb {R}^d))$$ and $$\eta \in AC([0,T];C_b({\mathbb {R}^{2d}_{\!\scriptscriptstyle \diagup }}))$$.

#### Theorem 5.1

Let $\Phi$ be a jointly antisymmetric admissible flux interpolation as in Definition 2.2 and consider an initial datum $(r^0, \eta ^0) \in L^\infty _\mu (\mathbb {R}^d)\times C_b({\mathbb {R}^{2d}_{\!\scriptscriptstyle \diagup }})$. Assume that $V:[0,T]\times L^\infty _\mu (\mathbb {R}^d) \rightarrow \mathcal {V}^{as}({\mathbb {R}^{2d}_{\!\scriptscriptstyle \diagup }})$ satisfies (5.3) and, for any $g,\tilde{g}\in L^\infty _\mu (\mathbb {R}^d)$,

5.4 $$\begin{aligned} \sup _{t\in [0,T]} \sup _{(x,x')\in {\mathbb {R}^{2d}_{\!\scriptscriptstyle \diagup }}} |V_t(g_t(x),g_t(x')) - V_t(\tilde{g}_t(x),\tilde{g}_t(x'))| \le L_{V}\Vert g-\tilde{g} \Vert _{L^\infty _\mu (\mathbb {R}^d)}, \end{aligned}$$

for a constant $$ L_{V}>0$$. Assume that $$\omega :[0,T]\times L^\infty _\mu (\mathbb {R}^d) \times {\mathbb {R}^{2d}_{\!\scriptscriptstyle \diagup }}\rightarrow \mathbb {R}$$ satisfies $$(\tilde{{\boldsymbol{\omega }}}1)$$–$$(\tilde{{\boldsymbol{\omega }}}4)$$. Then, there exists a solution $$(r, \eta )$$ to (Euler Co-NCL) with initial data $$(r^0,\eta ^0)$$, in the sense of Definition 3.1, for $$p=\infty $$.

### Long-Time Behaviour

Let us now focus on the long-time asymptotics for (Euler Co-NCL). We assume the vertices of the graph belong to a compact space, i.e. $\mathop {\textrm{supp}}\limits \mu = K \subset \mathbb {R}^d$ for *K* compact. Analogously to the previous section, we use the following notation ${K^2_{\!\scriptscriptstyle \diagup }}= \{(x,y) \in K^2\ | \ x \ne y\}$. The class of velocities we have in mind is as follows.

#### Assumption 5.1

Assume that $V:[0,\infty ) \times L_\mu ^\infty (K)\rightarrow \mathcal {V}^{as}({K^2_{\!\scriptscriptstyle \diagup }})$ is continuous in time and is of the form

$$ V(r_t(x),r_t(x')) = \alpha (r_t(x)) - \alpha (r_t(x')), $$

where $\alpha \in C^1_b(\mathbb {R})$ is increasing on compact sets, i.e. for any compact $\Omega \subset \mathbb {R}$

$$ \alpha '(y)=\frac{\hbox {d}}{\hbox {d}y}\alpha (y)>c>0\, \qquad \text{ for } y \in \Omega $$

and a constant *c*. We denote by ${\alpha '_*}:= \inf _{y \in \Omega } \alpha '(y)$.

Before proving the main result of this section, we recall some technical results—the first three are a direct adaptation of Bonnet et al. (2022), Theorems 2.1, 2.2, Lemma 2.3, respectively (see also Berliocchi and Lasry (1973) and Danskin (1967) for the original reference of Theorems 5.2 and 5.3, respectively).

#### Theorem 5.2

(Scorza-Dragoni) Let $\Omega \subset \mathbb {R}^d$ be a locally compact set and $f: \mathbb {R}_+ \times \Omega \rightarrow \mathbb {R}$ be such that $x \in \Omega \mapsto f(t,x) \in \mathbb {R}$ is $\mu$-measurable for each $t \ge 0$, and $t \in \mathbb {R}_+ \mapsto f(t,x) \in \mathbb {R}$ is continuous for $\mu$-almost every $x \in \Omega$. Then, for every $\varepsilon > 0$, there exists a compact set $\Omega _\varepsilon \subset \Omega$ satisfying $\mu (\Omega \setminus \Omega _\varepsilon ) < \varepsilon$ and such that the restricted map $f: \mathbb {R}_+ \times \Omega _\varepsilon \rightarrow \mathbb {R}$ is continuous.

#### Theorem 5.3

(Danskin) Let $\Omega \subset \mathbb {R}^d$ be a compact set and $f: \mathbb {R}_+ \times \Omega \rightarrow \mathbb {R}$ be a continuous function such that $t \in \mathbb {R}_+ \mapsto f(t,x) \in \mathbb {R}$ is differentiable for all $x \in \Omega$. Then, the application $g: t \in \mathbb {R}_+ \mapsto \max _{x \in \Omega } f(t,x) \in \mathbb {R}$ is differentiable $\mathscr {L}^1$-almost everywhere, with

$$ \frac{d}{dt} g(t) = \max _{x \in \overline{\Omega }(t)} \partial _t f(t,x) $$

for $\mathscr {L}^1$-almost every $t \ge 0$, where we introduced the notation $\overline{\Omega }(t):= \arg \max _{x \in \Omega } f(t,x)$.

#### Lemma 5.1

(Interior estimates for supremums) Let $\Omega \subset \mathbb {R}^d$ be a compact set and $f \in L^\infty _\mu (\Omega , \mathbb {R}^d)$. Then, for every $\delta > 0$, there exists $\varepsilon > 0$ such that

5.5 $$\begin{aligned} \mathop {\mathrm {ess\,sup}}\limits _{x \in \Omega }f(x) - \delta \le \mathop {\mathrm {ess\,sup}}\limits _{x \in \Omega ^\varepsilon }f(x) \le \mathop {\mathrm {ess\,sup}}\limits _{x \in \Omega } f(x), \end{aligned}$$

whenever $\Omega _\varepsilon \subset \Omega$ is a measurable set satisfying $\mu (\Omega \setminus \Omega _\varepsilon ) < \varepsilon$. In particular, it holds that

$$ \mathop {\mathrm {ess\,sup}}\limits _{x \in \Omega ^\varepsilon }f(x) \xrightarrow {\varepsilon \rightarrow 0^+} \mathop {\mathrm {ess\,sup}}\limits _{x \in \Omega }f(x), $$

for each family of sets $(\Omega _\varepsilon )_{\varepsilon >0} \subset \textrm{P}(\Omega )$ satisfying these properties.

#### Proof

The second inequality in (5.5) is satisfied for any subset of $\Omega$. For the first inequality, by definition of the essential supremum of a set, for every $\delta >0$ there exists a set $\Omega _\delta \subset \Omega$ of positive measure such that

$$ f(x) > \mathop {\mathrm {ess\,sup}}\limits _{x\in \Omega } f(x) - \delta $$

for $\mu$-a.e. $x \in \Omega _\delta$. Then, by choosing $\varepsilon >0$ satisfying $\varepsilon <\mu (\Omega _\delta )$ and considering any closed set $\Omega _\varepsilon \subset \Omega$ with $\mu (\Omega \backslash \Omega _\varepsilon ) < \varepsilon$, it necessarily holds that $\mu (\Omega _\delta \cap \Omega _\varepsilon ) >0$. Indeed assuming the measure is 0, by contradiction, one would have

$$ \mu (\Omega _\delta \cup \Omega _\varepsilon ) = \mu (\Omega _\delta ) + \mu (\Omega _\varepsilon )> \mu (\Omega ), $$

which is a contradiction since both sets are subsets of $\Omega$. Hence, by construction we have

$$ f(x) > \mathop {\mathrm {ess\,sup}}\limits _{x\in \Omega } f(x) - \delta , $$

for $\mu$-a.e. $x \in \Omega _\delta \cap \Omega _{\varepsilon }$, which concludes the proof. $\square$

We will also resort to the following comparison results.

#### Lemma 5.2

Let $\phi :\mathbb {R}\rightarrow \mathbb {R}$ be a locally-Lipschitz function. Assume that $f\in C(\mathbb {R}^+,\mathbb {R})$ solves the inequality $f(t) \le f(0) + \int _0^t\phi (f(s))\textrm{d}s$ with $f(0)=a \in \mathbb {R}$ and $g\in C(\mathbb {R}^+,\mathbb {R})$ solves the equation $g(t) = g(0) + \int _0^t\phi (g(s))\textrm{d}s$ with $f(0)\le g(0)$. Then, for $t\ge 0$, $f(t) \le g(t)$.

#### Proof

Let $h(t):= f(t) - g(t)$ and, by contradiction, assume there is $b > 0$ such that $h(b) > 0$. By continuity of *h*, Bolzano’s theorem implies that there must be a $c \in {[0},b)$ such that $h(t) \ge 0$ for $t \in (c,b]$ and $h(c) = 0$. Then, for $t \in (c,b]$, we have

$$\begin{aligned} h(t)&\le h(c) + \int _c^t\phi (f(s)) - \phi (g(s))\textrm{d}s\\&\le h(c) + L\int _c^t(f(s) - g(s))\textrm{d}s \le h(c) + L \int _c^t h(s)\textrm{d}s, \end{aligned}$$

where, for $t\in (c,b]$, $L>0$ is the local-Lipschitz constant of $\phi$. Then, by Grönwall’s inequality

$$ h(b) \le h(c)e^{L(b-c)} = 0 \, $$

which gives the contradiction. $\square$

The opposite inequality holds and can be proven in a similar way.

#### Lemma 5.3

Let $\phi :\mathbb {R}\rightarrow \mathbb {R}$ be a locally-Lipschitz function. Assume that $f\in C(\mathbb {R}^+,\mathbb {R})$ solves the inequality $f(t) \ge f(0) + \int _0^t\phi (f(s))\textrm{d}s$ with $f(0)=a \in \mathbb {R}$ and $g\in C(\mathbb {R}^+,\mathbb {R})$ solves the equation $g(t) = g(0) + \int _0^t\phi (g(s))\textrm{d}s$ with $f(0)\ge g(0)$. Then for, $t\ge 0$, $f(t) \ge g(t)$

Throughout this section, we consider (Euler Co-NCL) on the time domain $[0,\infty )$. The well-posedness follows upon adapting assumptions $(\tilde{{\boldsymbol{\omega }}}1)$–$(\tilde{{\boldsymbol{\omega }}}3)$, (5.2), (5.3) and (5.4) to hold on $[0, \infty )$, and noticing the solution can be extended to $[0,\infty )$ in view of the bounds on the respective norms. To obtain our long-time behaviour result, we need to impose some further conditions on $\eta$. First, let us recall that we can obtain an explicit representation for $\eta$ given by

5.6 $$\begin{aligned} \eta _t(x,y) = e^{-t}\eta _0(x,y) + \int _0^t e^{-(t-s)}\omega _s[r](x,y) \textrm{d}s\ . \end{aligned}$$

We refer the reader to (Mikolas 2024, Lemma A.0.1) for a derivation of (5.6). Using (5.6), we can obtain positivity preservation for the edge-weight function $\eta$ as well as a sharper bound on its supremum norm which we present in the following two results.

#### Lemma 5.4

Assume that $(r,\eta ) \in AC([0,\infty ), L_\mu ^\infty (\mathbb {R}^d))\times AC([0,\infty ), C_b({K^2_{\!\scriptscriptstyle \diagup }}))$ is the solution of (Euler Co-NCL) with initial datum $(r_0,\eta _0) \in (L_\mu ^\infty (\mathbb {R}^d),C_b({K^2_{\!\scriptscriptstyle \diagup }}))$. If, in addition to Assumptions $(\tilde{{\boldsymbol{\omega }}}1)$–$(\tilde{{\boldsymbol{\omega }}}3)$, we impose

5.7 $$\begin{aligned} \omega _*:=\inf _{t \in [0,\infty )} \inf _{(x,x') \in {K^2_{\!\scriptscriptstyle \diagup }}} \inf _{r \in L_\mu ^\infty (\mathbb {R}^d)\backslash \{0\}}\omega _t[r](x,x') > 0\ , \end{aligned}$$

and $\eta _0>0$, then $\eta _t >0$ for $t \in [0,\infty )$. Furthermore, if $\eta ^0 \in C_b({K^2_{\!\scriptscriptstyle \diagup }})$ is symmetric, and $\omega$ satisfies assumption $(\tilde{{\boldsymbol{\omega }}}4)$ for any $t\in [0,\infty )$ and $r\in L_\mu ^\infty (\mathbb {R}^d)$, $\eta _t$ is symmetric for $t \in [0,\infty )$.

#### Proof

Using the representation for $\eta$ in (5.6), we have, for any $(x,x') \in {K^2_{\!\scriptscriptstyle \diagup }}$ and $t\ge 0$,

$$\begin{aligned} \eta _t(x,x')&= e^{-t }\left( \eta _0(x, x')+\int _0^t e^{s} \omega _s\left[ r\right] (x, x') \textrm{d} s\right) \ \\&\ge \inf _{(x,x')\in {K^2_{\!\scriptscriptstyle \diagup }}}\eta _0(x,x')e^{-t} + \omega _*(1-e^{-t}) > 0 \end{aligned}$$

by (5.7) and $\eta _0>0$. The claim about symmetry follows directly from the representation (5.6). $\square$

#### Lemma 5.5

Let $(r,\eta ) \in AC([0,T], L_\mu ^\infty (\mathbb {R}^d))\times AC([0,T], C_b({K^2_{\!\scriptscriptstyle \diagup }}))$ solve (Euler Co-NCL) with initial datum $(r_0,\eta _0) \in (L_\mu ^\infty (\mathbb {R}^d),C_b({K^2_{\!\scriptscriptstyle \diagup }}))$, where $\omega$ satisfies $(\tilde{{\boldsymbol{\omega }}}1)$–$(\tilde{{\boldsymbol{\omega }}}4)$. Then,

5.8 $$\begin{aligned} \Vert \eta _t \Vert _{\infty ,C_b({K^2_{\!\scriptscriptstyle \diagup }})} \le \Vert \eta _0 \Vert _{\infty ,C_b({K^2_{\!\scriptscriptstyle \diagup }})} + C_{\omega }\ . \end{aligned}$$

#### Proof

The proof follows directly from the representation (5.6). $\square$

In what follows, we will use the notation

$$ \eta _*:= \inf _{t \in [0,\infty )}\inf _{(x,x')\in {K^2_{\!\scriptscriptstyle \diagup }}}\eta _t(x,x') \, $$

and note that we have $\eta _*>0$ in view of Lemma 5.4. The following theorem holds.

#### Theorem 5.4

Fix $\Phi \equiv \Phi _{upwind}$. Let $(r,\eta )\in AC(\mathbb {R}^+,L_\mu ^\infty (K)) \times AC(\mathbb {R}^+,C_b({K^2_{\!\scriptscriptstyle \diagup }}))$ be a solution of (Euler Co-NCL) with a pointwise monotonic velocity field $V:\mathbb {R}^+\times L_\mu ^\infty (K)\rightarrow \mathcal {V}^{as}({K^2_{\!\scriptscriptstyle \diagup }})$ satisfying (5.3), (5.4), and Assumption 5.1. Assume $\omega :\mathbb {R}^+\times L_\mu ^\infty (K)\times {K^2_{\!\scriptscriptstyle \diagup }}\rightarrow \mathbb {R}$ satisfies $(\tilde{{\boldsymbol{\omega }}}1)$–$(\tilde{{\boldsymbol{\omega }}}4)$, and (5.7). Suppose $r_0 \ge 0$ and $\int _{K}r_0(x)\textrm{d}\mu (x) = M$ and that $\eta ^0 \in C_b({K^2_{\!\scriptscriptstyle \diagup }})$ is symmetric and $\eta _0 >0$. Then, we have

5.9a $$\begin{aligned} \Vert r\Vert _{\infty ,L^\infty _\mu (K)}\le \Vert r_0\Vert _{L^\infty _\mu (K)}, \end{aligned}$$

and, for any $t\ge 0$,

5.9b $$\begin{aligned} \Vert r_t \Vert _{L_\mu ^\infty (K)} \le \frac{\Vert r_0 \Vert _{L_\mu ^\infty (K)}\alpha '_{*,0}\eta _{*,0}Me^{\alpha '_{*,0}\eta _*Mt}}{\alpha '_{*,0}\eta _*\left[ \Vert r_0 \Vert _{L_\mu ^\infty (K)}\mu (K)(e^{\alpha '_{*,0}\eta _*Mt} - 1) + M\right] }, \end{aligned}$$

where $\alpha '_{*,0}$ indicates that $\alpha '_*$ depends on $r_0$.

#### Proof

We begin by proving (5.9a) which is needed for the second result. Let us note that, given our assumptions on $\omega$ and $\eta ^0$, we have that $\eta _t$ is positive and symmetric for $t \ge 0$. Thus, since we are restricting to the upwind interpolation, by Esposito et al. (2023), Proposition 5.2, (Euler Co-NCL) is non-negativity preserving on $t\ge 0$. Next, fix $\varepsilon >0$ and observe that by Theorem 5.2 there exists a compact set $K^\varepsilon \subset K$ such that the restriction $r:\mathbb {R}^+ \times K^\varepsilon \rightarrow \mathbb {R}$ is continuous and $\mu (K\backslash K^\varepsilon ) < \varepsilon$. For all $t \ge 0$, we define the family of restricted supremum norms

$$ L_\varepsilon (t):= \max _{x \in K^\varepsilon } |r_t(x)| . $$

Since $(r,\eta )$ is the solution of (Euler Co-NCL), we have $r \in AC([0,\infty ),L^\infty _\mu (\mathbb {R}^d))$. As any absolutely continuous curve can be reparametrized in time to be Lipschitz continuous (see, e.g. Santambrogio (2015), Box 5.1), the mapping $L_\varepsilon (t)$ is Lipschitz, being the pointwise maximum of a family of equi-Lipschitz continuous functions. In particular, it is differentiable $\mathscr {L}^1$-almost everywhere by Rademacher’s theorem. Let us denote by $\mathbb {V}_\varepsilon (t):= \mathop {\mathrm {arg\,max}}\limits _{x \in K^\varepsilon }r_t(x)$. Then, by Theorem 5.3 we have

$$ \partial _t L_\varepsilon (t) = \max _{x \in \mathbb {V}_\varepsilon (t) }\partial _t r_t(x) . $$

Thus, taking an arbitrary vertex $x \in \mathbb {V}_\varepsilon (t)$, we have

5.10 $$\begin{aligned} \partial _t r_t(x)&= -r_t(x) \int _{K^\varepsilon \backslash \{x\}}V_t^+(r_t(x),r_t(x'))\eta _t(x,x')\textrm{d}\mu (x') \nonumber \\&\quad -r_t(x) \int _{K\backslash K^\varepsilon }V_t^+(r_t(x),r_t(x'))\eta _t(x,x')\textrm{d}\mu (x') \\&\quad + \int _{K^\varepsilon \backslash \{x\}}r_t(x')V_t^-(r_t(x),r_t(x'))\eta _t(x,x') \textrm{d}\mu (x')\nonumber \\&\quad + \int _{K\backslash K^\varepsilon }r_t(x')V_t^-(r_t(x),r_t(x'))\eta _t(x,x') \textrm{d}\mu (x')\ . \nonumber \end{aligned}$$

For any $x \in \mathbb {V}_\varepsilon (t)$, it holds

$$ \int _{K^\varepsilon \backslash \{x\}} r_t(x')V_t^-(r_t(x),r_t(x')) \eta _t(x,x') \textrm{d}\mu (x') = 0, $$

since $V_t^-(r_t(x),r_t(x')) = \big (\alpha (r_t(x')) - \alpha (r_t(x))\big )_{+}=0$, $x \in \mathbb {V}_\varepsilon (t)$, $x'\in K^\varepsilon \backslash \{x\}$ and $\alpha$ is monotonically increasing. Furthermore, since $\int _{K\backslash K^\varepsilon }V_t^+(r_t(x),r_t(x'))\eta _t(x,x')\textrm{d}\mu (x') \ge 0$, by $r_t \ge 0$ we infer

$$\begin{aligned} \partial _t r_t(x)&\le \ -r_t(x) \int _{K^\varepsilon \backslash \{x\}}V_t^+(r_t(x),r_t(x'))\eta _t(x,x')\textrm{d}\mu (x') \\&\quad + \int _{K\backslash K^\varepsilon }r_t(x')V_t^-(r_t(x),r_t(x'))\eta _t(x,x') \textrm{d}\mu (x')\ . \end{aligned}$$

In particular, recalling that for any $x \in \mathbb {V}_\varepsilon (t)$ we have $r_t(x) = L_\varepsilon (t)$, we can rewrite

5.11 $$\begin{aligned} {\begin{matrix} \partial _t L_\varepsilon (t) & \le \ -L_\varepsilon (t) \int _{K^\varepsilon \backslash \{x\}}V_t^+(L_\varepsilon (t),r_t(x'))\eta _t(x,x')\textrm{d}\mu (x') \\ & \quad + \int _{K\backslash K^\varepsilon }r_t(x')V_t^-(L_\varepsilon (t),r_t(x'))\eta _t(x,x') \textrm{d}\mu (x')\ \\ & \le C_V\Vert r_t \Vert _{L_\mu ^\infty (K)}\Vert \eta \Vert _{\infty ,C_b({K^2_{\!\scriptscriptstyle \diagup }})}\varepsilon \ , \end{matrix}} \end{aligned}$$

where we used the boundedness of *V* and $\eta$ and that $\mu (K\backslash K^\varepsilon ) < \varepsilon$. Integrating with respect to time yields

$$\begin{aligned} L_\varepsilon (t)&\le L_\varepsilon (0) +\varepsilon \int _0^tC_V\Vert r_s \Vert _{L_\mu ^\infty (K)}\Vert \eta \Vert _{\infty ,C_b({K^2_{\!\scriptscriptstyle \diagup }})} \textrm{d}s\ . \end{aligned}$$

Letting $\varepsilon \rightarrow 0^+$, Lemma 5.1 implies (5.9a), i.e.

$$\begin{aligned} \Vert r_t \Vert _{L_\mu ^\infty (\mathbb {R}^d)}&\le \Vert r_0 \Vert _{L_\mu ^\infty (\mathbb {R}^d)}. \end{aligned}$$

By repeating the same argument up to (5.11), we have

$$\begin{aligned} \partial _t L_\varepsilon (t)&\le -L_\varepsilon (t) \int _{K^\varepsilon \backslash \{x\}}V_t^+(L_\varepsilon (t),r_t(x'))\eta _t(x,x')\textrm{d}\mu (x') \\&\quad + \int _{K\backslash K^\varepsilon }r_t(x')V_t^-(L_\varepsilon (t),r_t(x'))\eta _t(x,x') \textrm{d}\mu (x')\ \\&\le \ -L_\varepsilon (t) \eta _*\int _{K^\varepsilon \backslash \{x\}}V_t^+(L_\varepsilon (t),r_t(x'))\textrm{d}\mu (x')\\&\quad + C_V\Vert r_t \Vert _{L_\mu ^\infty (K)}\Vert \eta \Vert _{\infty ,C_b({K^2_{\!\scriptscriptstyle \diagup }})}\varepsilon \ , \end{aligned}$$

where we used the boundedness of *V* and $\eta$ and that $\mu (K\backslash K^\varepsilon ) < \varepsilon$. The monotonicity of *V*, for $x \in \mathbb {V}_\varepsilon (t)$ and $\mu$-a.e. $x' \in K^\varepsilon$,

$$ V^+_t(L_\varepsilon (t), r_t(x')) = V_t(L_\varepsilon (t), r_t(x')) . $$

Furthermore, for velocities satisfying Assumption 5.1, the mean-value theorem gives

$$\begin{aligned} \alpha (L_\varepsilon (t)) - \alpha (r_t(x'))&= \alpha '(c_{t,x})(L_\varepsilon (t) - r_t(x')) \\&\ge {\alpha '_{*,0}}(L_\varepsilon (t) - r_t(x'))\ , \end{aligned}$$

for some $c_{t,x} \in [r_t(x'),L_\varepsilon (t)]\subset \Omega _0:=[0,\Vert r_0\Vert _{L^\infty _\mu (K)}]$, where $\alpha '_{*,0}: = \inf _{y\in \Omega _0}\alpha (y)$. Hence,

$$\begin{aligned} \partial _t L_\varepsilon (t)&\le \ -L_\varepsilon (t) \eta _*\alpha '_{*,0}\int _{K^\varepsilon \backslash \{x\}}(L_\varepsilon (t) - r_t(x'))\textrm{d}\mu (x') \\&\quad + C_V\Vert r_t \Vert _{L_\mu ^\infty (K)}\Vert \eta \Vert _{\infty ,C_b({K^2_{\!\scriptscriptstyle \diagup }})}\varepsilon \ , \\&= \ -L^2_\varepsilon (t) \eta _*\alpha '_{*,0}\mu (K^\varepsilon ) + \eta _*\alpha '_{*,0} L_\varepsilon (t)\int _{K^\varepsilon \backslash \{x\}}r_t(x')\textrm{d}\mu (x') \\&\quad + C_V\Vert r_t \Vert _{L_\mu ^\infty (K)}\Vert \eta \Vert _{\infty ,C_b({K^2_{\!\scriptscriptstyle \diagup }})}\varepsilon \ , \end{aligned}$$

whence, integrating with respect to time yields

$$\begin{aligned} L_\varepsilon (t)&\le L_\varepsilon (0) -\int _0^t \bigg [L^2_\varepsilon (s) \eta _*\alpha '_{*,0}\mu (K^\varepsilon ) - \eta _*\alpha '_{*,0} L_\varepsilon (s)\int _{K^\varepsilon \backslash \{x\}}r_s(x')\textrm{d}\mu (x')\bigg ]\textrm{d}s \\&\quad + \int _0^tC_V\Vert r_s \Vert _{L_\mu ^\infty (K)}\Vert \eta \Vert _{\infty ,C_b({K^2_{\!\scriptscriptstyle \diagup }})}\varepsilon \textrm{d}s\ . \end{aligned}$$

Letting $\varepsilon \rightarrow 0^+$, Lemma 5.1 implies

5.12 $$\begin{aligned} \Vert r_t \Vert _{L_\mu ^\infty (\mathbb {R}^d)}&\le \Vert r_0 \Vert _{L_\mu ^\infty (\mathbb {R}^d)} -\int _0^t \bigg [\Vert r_s \Vert ^2_{L_\mu ^\infty (\mathbb {R}^d)} \eta _*\alpha '_{*,0}\mu (K) - \eta _*\alpha '_{*,0} \Vert r_s \Vert _{L_\mu ^\infty (\mathbb {R}^d)}M\bigg ] \textrm{d}s\ , \end{aligned}$$

where we have used that $\int _{K}r_t(x)\textrm{d}\mu (x') = M$, by the conservation of mass property (see Mikolas (2024), Lemma A.0.2). Noting that (5.12) is an inequality version of a Bernoulli ODE (see Mikolas (2024), Lemma A.0.3), using Lemma 5.2 we infer

$$\begin{aligned} \Vert r_t \Vert _{L_\mu ^\infty (K)} \le \frac{\Vert r_0 \Vert _{L_\mu ^\infty (K)}\alpha '_{*,0}\eta _*Me^{\alpha '_{*,0}\eta _*Mt}}{\alpha '_{*,0}\eta _*\left[ \Vert r_0 \Vert _{L_\mu ^\infty (K)}\mu (K)(e^{\alpha '_{*,0}\eta _*Mt} - 1) + M\right] }\ , \end{aligned}$$

which gives the result. $\square$

Next, we find a lower bound for $r_{t,*}:= \inf _{x \in K} r_{t}(x)$. Note that since *r* is lower bounded and *K* is compact, the infimum and the minimum coincide.

#### Theorem 5.5

Fix $\Phi \equiv \Phi _{upwind}$. Let $(r,\eta )\in AC(\mathbb {R}^+,L_\mu ^\infty (K)) \times AC(\mathbb {R}^+,C_b({K^2_{\!\scriptscriptstyle \diagup }}))$ be a solution of (Euler Co-NCL) with a pointwise monotonic velocity field $V:\mathbb {R}^+\times L_\mu ^\infty (K)\rightarrow \mathcal {V}^{as}({K^2_{\!\scriptscriptstyle \diagup }})$ satisfying (5.3), (5.4), and Assumption 5.1. Let $\omega :\mathbb {R}^+\times L_\mu ^\infty (K)\times {K^2_{\!\scriptscriptstyle \diagup }}\rightarrow \mathbb {R}$ satisfy $(\tilde{{\boldsymbol{\omega }}}1)$–$(\tilde{{\boldsymbol{\omega }}}4)$. Assume $r_0 \ge 0$ and $\int _{K}r_0(x)\textrm{d}\mu (x) = M$ and $\eta ^0 \in C_b({K^2_{\!\scriptscriptstyle \diagup }})$ is positive and symmetric. Then, we have

5.13 $$\begin{aligned} r_{t,*} \ge \frac{r_{0,*}\eta _*\alpha '_{*,0}Me^{\eta _*\alpha '_{*,0}M t}}{\eta _*\alpha '_{*,0}(M+ (e^{\eta _*\alpha '_{*,0}M t}-1)\mu (K)r_{0,*})} \ . \end{aligned}$$

#### Proof

As in Theorem 5.4, by our assumptions on $\omega$, $r^0,\eta ^0$ and the use of the upwind interpolation, we have non-negativity preservation of *r* for $t\ge 0$ by Esposito et al. (2023), Proposition 5.2. Furthermore, let us consider the Lipschitz continuous reparametrization of the absolutely continuous curve $r_t$. By abuse of notation, let us denote $r_t^-:= -r_t$ and fix $\varepsilon >0$. By Theorem 5.2 there exists a compact set $K^\varepsilon \subset K$ such that the restriction $r^-:[0,T]\times K^\varepsilon \rightarrow \mathbb {R}$ is continuous and $\mu (K\backslash K^\varepsilon ) < \varepsilon$ for some $\varepsilon >0$. For all $t \ge 0$, define the family of restricted maxima on $K^\varepsilon$, i.e.

$$ L^-_\varepsilon (t):= \max _{x \in K^\varepsilon } r^-_t(x) . $$

Since $L^-_{\varepsilon }(\cdot )$ is the pointwise maximum of equi-Lipschitz continuous functions, Theorem 5.3 implies

5.14 $$\begin{aligned} \partial _t L^-_\varepsilon (t)&= \partial _t\max _{x \in \mathbb {V}^-_\varepsilon (t)} r_t^-(x) = \max _{x \in \mathbb {V}^-_\varepsilon (t)}\partial _t r_t^-(x)= \max _{x \in \mathbb {V}^-_\varepsilon (t)} -\partial _t r_t(x)\ , \end{aligned}$$

where $\mathbb {V}^-_\varepsilon (t) = \mathop {\mathrm {arg\,max}}\limits _{x \in K^\varepsilon } r^-_t(x)$. Then, for any vertex $x \in \mathbb {V}^-_\varepsilon (t)$ it holds that

$$\begin{aligned} \partial _t r^-_t(x)&= \ r_t(x) \int _{K^\varepsilon \backslash \{x\}}V_t^+(r_t(x),r_t(x'))\eta _t(x,x')\textrm{d}\mu (x') \\&\quad +r_t(x) \int _{K\backslash K^\varepsilon }V_t^+(r_t(x),r_t(x'))\eta _t(x,x')\textrm{d}\mu (x') \\&\quad - \int _{K^\varepsilon \backslash \{x\}}r_t(x')V_t^-(r_t(x),r_t(x'))\eta _t(x,x') \textrm{d}\mu (x') \\&\quad - \int _{K\backslash K^\varepsilon }r_t(x')V_t^-(r_t(x),r_t(x'))\eta _t(x,x') \textrm{d}\mu (x')\ . \end{aligned}$$

Note that $r^\varepsilon _{t,*}:= \min _{x \in K^\varepsilon } r_t(x)= - \max _{x \in K^\varepsilon } r^-_t(x)$. Then, in view of (5.14) we have for $x \in \mathbb {V}_{\varepsilon }^-(t)$ that

5.15 $$\begin{aligned} \partial _t r_{t}(x)&= - r_t(x) \int _{K^\varepsilon \backslash \{x\}}V_t^+(r_t(x),r_t(x'))\eta _t(x,x')\textrm{d}\mu (x') \nonumber \\&\quad -r_t(x) \int _{K\backslash K^\varepsilon }V_t^+(r_t(x),r_t(x'))\eta _t(x,x')\textrm{d}\mu (x') \\&\quad + \int _{K^\varepsilon \backslash \{x\}}r_t(x')V_t^-(r_t(x),r_t(x'))\eta _t(x,x') \textrm{d}\mu (x') \nonumber \\&\quad + \int _{K\backslash K^\varepsilon }r_t(x')V_t^-(r_t(x),r_t(x'))\eta _t(x,x') \textrm{d}\mu (x')\ \nonumber \end{aligned}$$

which is the evolution of $$ r_t(x)$$ for $$x \in \mathop {\mathrm {arg\,min}}\limits _{x\in K^\varepsilon } r_t(x)$$, since for any function *f* such that the maximum and the minimum exist, $$\mathop {\mathrm {arg\,max}}\limits -f =\mathop {\mathrm {arg\,min}}\limits f$$. Furthermore, this implies that

5.16 $$\begin{aligned} V_t^+(r_t(x), r_t(x')) = \bigg (\alpha (r_t(x)) - \alpha (r_t(x'))\bigg )_+ = 0\ , \end{aligned}$$

for $x \in \mathbb {V}^-_\varepsilon (t)$ and $x'\in K^\varepsilon \backslash \{x\}$, since $\mathbb {V}^-_\varepsilon (t) = \mathop {\mathrm {arg\,max}}\limits _{x \in K^\varepsilon } -r_t(x) = \mathop {\mathrm {arg\,min}}\limits _{x \in K^\varepsilon } r_t(x)$ and $\alpha$ is monotonic. In particular, the first term vanishes. Furthermore, noting that the last term is nonnegative, we can drop it to obtain the following lower bound

$$\begin{aligned} \partial _t r_{t}(x)&\ge - r_{t}(x) \int _{K\backslash K^\varepsilon }V_t^+(r_t(x),r_t(x'))\eta _t(x,x')\textrm{d}\mu (x') \\&\quad + \int _{K^\varepsilon \backslash \{x\}}r_t(x')V_t^-(r_t(x),r_t(x'))\eta _t(x,x') \textrm{d}\mu (x') \\&\ge -\Vert r_t \Vert _{L_\mu ^\infty (K)}C_V\Vert \eta \Vert _{\infty ,C_b({K^2_{\!\scriptscriptstyle \diagup }})}\varepsilon \\&\quad + \int _{K^\varepsilon \backslash \{x\}}r_t(x')\bigg (\alpha (r_t(x'))-\alpha (r_t(x))\bigg )_+\eta _t(x,x') \textrm{d}\mu (x') \\&= -\Vert r_t \Vert _{L_\mu ^\infty (K)}C_V\Vert \eta \Vert _{\infty ,C_b({K^2_{\!\scriptscriptstyle \diagup }})}\varepsilon \\&\quad + \int _{K^\varepsilon \backslash \{x\}}r_t(x')(\alpha (r_t(x'))-\alpha (r_t(x)))\eta _t(x,x') \textrm{d}\mu (x') \\&\ge \ -\Vert r_t \Vert _{L_\mu ^\infty (K)}C_V\Vert \eta \Vert _{\infty ,C_b({K^2_{\!\scriptscriptstyle \diagup }})}\varepsilon \\&\quad +\eta _*\alpha '_{*,0}\int _{K^\varepsilon \backslash \{x\}} r_t(x')(r_t(x')-r_t(x))\textrm{d}\mu (x') \end{aligned}$$

where in the second and third inequality we used that $x \in \mathbb {V}_\varepsilon ^-(t)= \mathop {\mathrm {arg\,min}}\limits _{x \in K^\varepsilon }r_t(x)$, and in the third inequality we also used the monotonicity of the velocity. Since for $x \in \mathbb {V}^-_\varepsilon (t)$, $r_t(x) = r^\varepsilon _{t,*}$, we have,

$$\begin{aligned} \partial _t r^\varepsilon _{t,*}&= -\Vert r_t \Vert _{L_\mu ^\infty (K)}C_V\Vert \eta \Vert _{\infty ,C_b({K^2_{\!\scriptscriptstyle \diagup }})}\varepsilon \\&\quad +\eta _*\alpha '_{*,0}\int _{K^\varepsilon } r_t(x')(r_t(x')-r^\varepsilon _{t,*})\textrm{d}\mu (x') \\&\ge -\Vert r_t \Vert _{L_\mu ^\infty (K)}C_V\Vert \eta \Vert _{\infty ,C_b({K^2_{\!\scriptscriptstyle \diagup }})}\varepsilon \\&\quad +\eta _*\alpha '_{*,0}\left( r^\varepsilon _{t,*}\int _{K^\varepsilon }r_{t}(x') \textrm{d}\mu (x') - (r^\varepsilon _{t,*})^2\mu (K^\varepsilon )\right) \ . \end{aligned}$$

Integrating in time, sending $\varepsilon \rightarrow 0^+$ and using Lemma 5.1, we obtain

5.17 $$\begin{aligned} r_{t,*} \ge \ r_{0,*} +\int _0^t \eta _*\alpha '_{*,0}( r_{s,*}M - (r_{s,*})^2\mu (K))\textrm{d}s , \end{aligned}$$

where we used that $\int _{K}r_t(x)\textrm{d}\mu (x) = M$ and the preservation of mass property. Noting that (5.17) resembles a Bernoulli ODE, Lemma 5.3 and (Mikolas 2024, Lemma A.0.3) imply

$$\begin{aligned} r_{t,*} \ge \frac{r_{0,*}\eta _*\alpha '_{*,0}Me^{\eta _*\alpha '_*M t}}{\eta _*\alpha '_{*,0}(M+ (e^{\eta _*\alpha '_{*,0}M t}-1)\mu (K)r_{0,*})} \ . \end{aligned}$$

$$\square $$

Now we are ready to obtain the long-time behaviour of the upwind (Euler Co-NCL) for pointwise monotonic velocities.

#### Theorem 5.6

Fix $\Phi \equiv \Phi _{upwind}$. Let $(r,\eta )\in AC(\mathbb {R}^+,L_\mu ^\infty (K)) \times AC(\mathbb {R}^+,C_b({K^2_{\!\scriptscriptstyle \diagup }}))$ be a solution of (Euler Co-NCL) with a pointwise monotonic velocity field $V:\mathbb {R}^+\times L_\mu ^\infty (K)\rightarrow \mathcal {V}^{as}({K^2_{\!\scriptscriptstyle \diagup }})$ satisfying (5.3), (5.4) and Assumption 5.1. Let $\omega :\mathbb {R}^+\times L_\mu ^\infty (K)\times {K^2_{\!\scriptscriptstyle \diagup }}\rightarrow \mathbb {R}$ satisfy $(\tilde{{\boldsymbol{\omega }}}1)$–$(\tilde{{\boldsymbol{\omega }}}4)$. Assume $r_0 \ge 0$ and $\int _{K}r_0(x)\textrm{d}\mu (x) = M$, and $\eta ^0 \in C_b({K^2_{\!\scriptscriptstyle \diagup }})$ is positive and symmetric. Then, for $\mu$-a.e. $x \in \mathbb {R}^d$, it holds

$$ \lim _{t \rightarrow \infty }r_t(x) = \frac{M}{\mu (K)}, $$

the uniform distribution of mass over *K*.

#### Proof

For $\mu$-a.e. $x\in \mathbb {R}^d$ and $t\ge 0$, we have, by (5.9b) and (5.13), that

$$\begin{aligned}&\frac{r_{0,*}\eta _*\alpha '_{*,0}Me^{\eta _*\alpha '_{*,0}M t}}{\eta _*\alpha '_{*,0}(M+ (e^{\eta _*\alpha '_{*,0}M t}-1)\mu (K)r_{0,*})}\\&\quad \le r_{t,*} \\&\quad \le r_t(x) \\&\quad \le \Vert {r_t} \Vert _{{L_\mu ^\infty (K)}}\ \\&\quad \le \frac{\Vert r_0 \Vert _{L_\mu ^\infty (K)}\alpha '_{*,0}\eta _*Me^{\alpha '_{*,0}\eta _*Mt}}{\alpha '_{*,0}\eta _*\left[ \Vert r_0 \Vert _{L_\mu ^\infty (K)}\mu (K)(e^{\alpha '_{*,0}\eta _*Mt} - 1) + M\right] }\ , \end{aligned}$$

and letting $t \rightarrow \infty$ yields the result. $\square$

As a direct consequence of the previous theorem and the results in Sect. 4, we have the following corollary for the long-time asymptotics of the $\mu$-monokinetic solutions to (graph-CE).

#### Corollary 5.1

Let $\Phi \equiv \Phi _{upwind}$ and let $(r,\eta )\in AC(\mathbb {R}^+,L_\mu ^\infty (K)) \times AC(\mathbb {R}^+,C_b({K^2_{\!\scriptscriptstyle \diagup }}))$ be a solution of (Euler Co-NCL) with a pointwise monotonic velocity field $V:\mathbb {R}^+\times L_\mu ^\infty (K)\rightarrow \mathcal {V}^{as}({K^2_{\!\scriptscriptstyle \diagup }})$ satisfying (5.3), (5.4) and Assumption 5.1. Let $\omega :\mathbb {R}^+\times L_\mu ^\infty (K)\times {K^2_{\!\scriptscriptstyle \diagup }}\rightarrow \mathbb {R}$ satisfy $(\tilde{{\boldsymbol{\omega }}}1)$–$(\tilde{{\boldsymbol{\omega }}}4)$. Assume $r_0 \ge 0$ and $\int _{K}r_0(x)\textrm{d}\mu (x) = M$, and $\eta ^0 \in C_b({K^2_{\!\scriptscriptstyle \diagup }})$ is positive and symmetric. Then, the $\mu$-monokinetic solution to (graph-CE) weakly-* converges to $\bar{\sigma }=\delta _{M/\mu (K)}\otimes \mu$, as $t\rightarrow \infty$.

#### Proof

The $\mu$-monokinetic solution to (graph-CE) is given by $\sigma _t=\delta _{r_t}\otimes \mu$. One can check, indeed, that $\sigma _t$ satisfies (4.5) for $\varphi \in C_c^1([0,+\infty )\times \mathbb {R}\times K)$. Due to Theorem 5.6, for any $f\in C_0(\mathbb {R}\times K)$ it holds

$$\begin{aligned} \lim _{t\rightarrow \infty }\int _{\mathbb {R}\times K}f(\xi ,x)\textrm{d}\delta _{r_t(x)}\otimes \mu (\xi ,x)&=\lim _{t\rightarrow \infty }\int _{K}f(r_t(x),x)\textrm{d}\mu (x)\\&=\int _{K}f(M/\mu (K),x)\textrm{d}\mu (x), \end{aligned}$$

whence the thesis. $\square$

## Data Availability

No datasets were generated or analysed during the current study.

## References

[CR1] Ayi, N., Duteil, N.P.: Graph limit for interacting particle systems on weighted random graphs. arXiv:2307.12801 (2023)

[CR2] Ayi, N., Duteil, N.P.: Large-population limits of non-exchangeable particle systems. arXiv:2401.07748 (2024)

[CR3] Ayi, N., Duteil, N.P., Poyato, D.: Mean-field limit of non-exchangeable multi-agent systems over hypergraphs with unbounded rank. arXiv:2406.04691 (2024)

[CR4] Backhausz, A., Szegedy, B.: Action convergence of operators and graphs. Can. J. Math. **74**(1), 72–121 (2022)

[CR5] Ben-Porat, I., Carrillo, J.A., Galtung, S.T.: Mean field limit for one dimensional opinion dynamics with coulomb interaction and time dependent weights. Nonlinear Anal. **240**, 113462 (2024a)

[CR6] Ben-Porat, I., Carrillo, J.A., Jabin, P.-E.: The graph limit for a pairwise competition model. J. Differ. Equ. **413**, 329–369 (2024b)

[CR7] Berliocchi, H., Lasry, J.-M.: Intégrandes normales et mesures paramétrées en calcul des variations. Bull. Soc. Math. Fr. **101**, 129–184 (1973)

[CR8] Berner, R., Gross, T., Kuehn, C., Kurths, J., Yanchuk, S.: Adaptive dynamical networks. Phys. Rep. **1031**, 1–59 (2023)

[CR9] Bianchi, D., Setti, A.G., Wojciechowski, R.K.: The generalized porous medium equation on graphs: existence and uniqueness of solutions with data. Calc. Var. Partial. Differ. Equ. **61**(5), 171 (2022)

[CR10] Bonnet, B., Duteil, N.P., Sigalotti, M.: Consensus formation in first-order graphon models with time-varying topologies. Math. Models Methods Appl. Sci. **32**(11), 2121–2188 (2022)

[CR11] Caponigro, M., Fornasier, M., Piccoli, B., Trélat, E.: Sparse stabilization and optimal control of the Cucker–Smale model. Math. Control Relat. Fields **3**(4), 447–466 (2013)

[CR12] Caponigro, M., Fornasier, M., Piccoli, B., Trélat, E.: Sparse stabilization and control of alignment models. Math. Models Methods Appl. Sci. **25**(3), 521–564 (2015)

[CR13] Chen, Y., Georgiou, T.T., Tannenbaum, A.: Vector-valued optimal mass transport. SIAM J. Appl. Math. **78**(3), 1682–1696 (2018)

[CR14] Chiba, H., Medvedev, G.S.: The mean field analysis of the Kuramoto model on graphs, I: the mean field equation and transition point formulas. Discrete Contin. Dyn. Syst. **39**(1), 131–155 (2019a)

[CR15] Chiba, H., Medvedev, G.S.: The mean field analysis of the Kuramoto model on graphs, II: asymptotic stability of the incoherent state, center manifold reduction, and bifurcations. Discrete Contin. Dyn. Syst. **39**(7), 3897–3921 (2019b)

[CR16] Chow, S.-N., Huang, W., Li, Y., Zhou, H.: Fokker-Planck equations for a free energy functional or Markov process on a graph. Arch. Ration. Mech. Anal. **203**(3), 969–1008 (2012)

[CR17] Clopath, C., Büsing, L., Vasilaki, E., Gerstner, W.: Connectivity reflects coding: a model of voltage-based stdp with homeostasis. Nat. Neurosci. **13**(3), 344–352 (2010) 20098420 10.1038/nn.2479

[CR18] Craig, K., García Trillos, N., Slepčev, D.: Clustering dynamics on graphs: from spectral clustering to mean shift through Fokker–Planck interpolation. In: Active Particles, Volume 3: Advances in Theory, Models, and Applications, Springer, pp. 105–151 (2021)

[CR19] Dalmao, F., Mordecki, E.: Cucker–Smale flocking under hierarchical leadership and random interactions. SIAM J. Appl. Math. **71**(4), 1307–1316 (2011)

[CR20] Danskin, J.M.: The theory of max–min and its application to weapons allocation problems. In: Econometrics and Operations Research, Volume V. Springer, New York (1967)

[CR21] Demirel, G., Barter, E., Gross, T.: Dynamics of epidemic diseases on a growing adaptive network. Sci. Rep. **7**(1), 42352 (2017) 28186146 10.1038/srep42352 PMC5301221

[CR22] Disser, K., Liero, M.: On gradient structures for Markov chains and the passage to Wasserstein gradient flows. Netw. Heterog. Media **10**(2), 233–253 (2015)

[CR23] El Bouchairi, I., Fadili, J.M., Elmoataz, A.: Continuum limit of -Laplacian evolution problems on graphs: graphons and sparse graphs. ESAIM Math. Model. Numer. Anal. **57**(3), 1795–1838 (2023)

[CR24] Esposito, A., Heinze, G., Pietschmann, J.-F., Schlichting, A.: Graph-to-local limit for a multi-species nonlocal cross-interaction system. PAMM **23**(4), e202300094 (2023)

[CR25] Esposito, A., Heinze, G., Schlichting, A.: Graph-to-local limit for the nonlocal interaction equation. J. Math. Pures Appl. **194**, 103663 (2025)

[CR26] Esposito, A., Mikolás, L.: On evolution PDEs on co-evolving graphs. Discrete Contin. Dyn. Syst. **44**(9), 2660–2683 (2024)

[CR27] Esposito, A., Patacchini, F.S., Schlichting, A.: On a class of nonlocal continuity equations on graphs. Eur. J. Appl. Math. **35**(1), 109–126 (2023)

[CR28] Esposito, A., Patacchini, F.S., Schlichting, A., Slepčev, D.: Nonlocal-interaction equation on graphs: gradient flow structure and continuum limit. Arch. Ration. Mech. Anal. **240**(2), 699–760 (2021)

[CR29] Evans, L.C.: Partial differential Equations, Volume 19 of Graduate Studies in Mathematics, 2nd edn. American Mathematical Society, Providence (2010)

[CR30] Favre, G., Jankowiak, G., Merino-Aceituno, S., Trussardi, L.: An application-oriented framework for continuum modeling of opinion dynamics on a network. arXiv:2406.18076 (2024)

[CR31] Folland, G B.: Real analysis. In: Pure and Applied Mathematics (New York), Modern Techniques and Their Applications, 2nd edn. Wiley, New York (1999)

[CR32] Forkert, D., Maas, J., Portinale, L.: Evolutionary -convergence of entropic gradient flow structures for Fokker-Planck equations in multiple dimensions. SIAM J. Math. Anal. **54**(4), 4297–4333 (2022)

[CR33] García Trillos, N., Gerlach, M., Hein, M., Slepčev, D.: Error estimates for spectral convergence of the graph Laplacian on random geometric graphs toward the Laplace-Beltrami operator. Found. Comput. Math. **20**(4), 827–887 (2020)

[CR34] García Trillos, N., Slepčev, D.: Continuum limit of total variation on point clouds. Arch. Ration. Mech. Anal. **220**(1), 193–241 (2016)

[CR35] García Trillos, N., Slepčev, D.: A variational approach to the consistency of spectral clustering. Appl. Comput. Harmon. Anal. **45**(2), 239–281 (2018)

[CR36] Gkogkas, M.A., Kuehn, C.: Graphop mean-field limits for Kuramoto-type models. SIAM J. Appl. Dyn. Syst. **21**(1), 248–283 (2022)

[CR37] Gkogkas, M.A., Kuehn, C., Xu, C.: Continuum limits for adaptive network dynamics. Commun. Math. Sci. **21**(1), 83–106 (2023)

[CR38] Gkogkas, M.A., Kuehn, C., Xu, C.: Mean field limits of co-evolutionary signed heterogeneous networks. In: European Journal of Applied Mathematics, pp. 1–44 (2025)

[CR39] Gladbach, P., Kopfer, E., Maas, J., Portinale, L.: Homogenisation of one-dimensional discrete optimal transport. J. Math. Pures Appl. **9**(139), 204–234 (2020)

[CR40] Gladbach, P., Kopfer, E., Maas, J., Portinale, L.: Homogenisation of dynamical optimal transport on periodic graphs. Calc. Var. Partial. Differ. Equ. **62**(5), 1 (2023) 10.1007/s00526-023-02472-z PMC10147821 37131846

[CR41] Haskovec, J.: Graph limit of the consensus model with self-delay. J. Phys. A: Math. Theor. **57**(34), 345203 (2024)

[CR42] Heinze, G., Pietschmann, J.-F., Schmidtchen, M.: Nonlocal cross-interaction systems on graphs: energy landscape and dynamics. Kinet. Relat. Models **16**(6), 795–827 (2023a)

[CR43] Heinze, G., Pietschmann, J.-F., Schmidtchen, M.: Nonlocal cross-interaction systems on graphs: nonquadratic Finslerian structure and nonlinear mobilities. SIAM J. Math. Anal. **55**(6), 7039–7076 (2023b)

[CR44] Hraivoronska, A., Schlichting, A., Tse, O.: Variational convergence of the Scharfetter–Gummel scheme to the aggregation-diffusion equation and vanishing diffusion limit. Numer. Math. **156**(6), 2221–2292 (2024)

[CR45] Hraivoronska, A., Tse, O.: Diffusive limit of random walks on tessellations via generalized gradient flows. SIAM J. Math. Anal. **55**(4), 2948–2995 (2023)

[CR46] Kaliuzhnyi-Verbovetskyi, D., Medvedev, G.S.: The mean field equation for the Kuramoto model on graph sequences with non-Lipschitz limit. SIAM J. Math. Anal. **50**(3), 2441–2465 (2018)

[CR47] Kuehn, C.: Network dynamics on graphops. New J. Phys. **22**, 11 (2020)

[CR48] Kuehn, C., Xu, C.: Vlasov equations on digraph measures. J. Differ. Equ. **339**, 261–349 (2022)

[CR49] Lancellotti, C.: On the Vlasov limit for systems of nonlinearly coupled oscillators without noise. Transp. Theory Stat. Phys. **34**(7), 523–535 (2005)

[CR50] Lovász, L.: Large networks and graph limits. In: American Mathematical Society Colloquium Publications, vol. 60. American Mathematical Society, Providence (2012)

[CR51] Maas, J.: Gradient flows of the entropy for finite Markov chains. J. Funct. Anal. **261**(8), 2250–2292 (2011)

[CR52] Medvedev, G.S.: The nonlinear heat equation on dense graphs and graph limits. SIAM J. Math. Anal. **46**(4), 2743–2766 (2014a)

[CR53] Medvedev, G.S.: The nonlinear heat equation on -random graphs. Arch. Ration. Mech. Anal. **212**(3), 781–803 (2014b)

[CR54] Mielke, A.: A gradient structure for reaction–diffusion systems and for energy–drift–diffusion systems. Nonlinearity **24**(4), 1329–1346 (2011)

[CR55] Mikolas, L.: Evolution PDEs on graphs and inverse problems in aggregation–diffusion equations. PhD Thesis, University of Oxford (2024)

[CR56] Moreau, L.: Stability of multiagent systems with time-dependent communication links. IEEE Trans. Automat. Control **50**(2), 169–182 (2005)

[CR57] Neunzert, H.: An introduction to the nonlinear Boltzmann–Vlasov equation. In: Kinetic Theories and the Boltzmann Equation (Montecatini, 1981), Volume 1048 of Lecture Notes in Mathemtics, Springer, Berlin, pp. 60–110 (1984)

[CR58] Olfati-Saber, R., Murray, R.M.: Consensus problems in networks of agents with switching topology and time-delays. IEEE Trans. Automat. Control **49**(9), 1520–1533 (2004)

[CR59] Paul, T., Trélat, E.: From microscopic to macroscopic scale equations: mean field, hydrodynamic and graph limits. arXiv:2209.08832 (2024)

[CR60] Santambrogio, F.: Optimal transport for applied mathematicians. In: Progress in Nonlinear Differential Equations and their Applications, vol. 87. Springer, Cham (2015)

[CR61] Sawicki, J., Berner, R., Löser, T., Schöll, E.: Modeling tumor disease and sepsis by networks of adaptively coupled phase oscillators. Front. Netw. Physiol. **1**, 730385 (2022) 36925568 10.3389/fnetp.2021.730385 PMC10013027

[CR62] Seneta, E.: Coefficients of ergodicity: structure and applications. Adv. Appl. Probab. **11**(3), 576–590 (1979)

[CR63] Slepčev, D., Warren, A.: Nonlocal Wasserstein distance: metric and asymptotic properties. Calc. Var. Partial. Differ. Equ. **62**(9), 238 (2023)

[CR64] Triesch, J.: A gradient rule for the plasticity of a neuron’s intrinsic excitability. In: International Conference on Artificial Neural Networks, Springer, pp. 65–70 (2005)

[CR65] Warren, A.: Gradient flow structure for some nonlocal diffusion equations. arXiv:2412.20969 (2024)

